# Clustering malignant cell states using universally variable genes

**DOI:** 10.1093/bib/bbad460

**Published:** 2023-12-11

**Authors:** Sang-Ho Yoon, Jin-Wu Nam

**Affiliations:** Department of Life Science, College of Natural Sciences, Hanyang University, Seoul 04763, Republic of Korea; Hanyang Institute of Advanced BioConvergence, Hanyang University, Seoul 04763, Republic of Korea; Hanyang Institute of Bioscience and Biotechnology, Bio-BigData Research Center, Hanyang University, Seoul 04763, Republic of Korea; Department of Life Science, College of Natural Sciences, Hanyang University, Seoul 04763, Republic of Korea; Hanyang Institute of Advanced BioConvergence, Hanyang University, Seoul 04763, Republic of Korea; Research Institute for Convergence of Basic Sciences, Hanyang University, Seoul 04763, Republic of Korea; Hanyang Institute of Bioscience and Biotechnology, Bio-BigData Research Center, Hanyang University, Seoul 04763, Republic of Korea

**Keywords:** scRNA-seq, feature selection, clustering, tumor microenvironment

## Abstract

Single-cell RNA sequencing (scRNA-seq) has revealed important insights into the heterogeneity of malignant cells. However, sample-specific genomic alterations often confound such analysis, resulting in patient-specific clusters that are difficult to interpret. Here, we present a novel approach to address the issue. By normalizing gene expression variances to identify universally variable genes (UVGs), we were able to reduce the formation of sample-specific clusters and identify underlying molecular hallmarks in malignant cells. In contrast to highly variable genes vulnerable to a specific sample bias, UVGs led to better detection of clusters corresponding to distinct malignant cell states. Our results demonstrate the utility of this approach for analyzing scRNA-seq data and suggest avenues for further exploration of malignant cell heterogeneity.

## BACKGROUND

Cancer is a complex and heterogeneous disease, but different types of cancer share common signatures and molecular hallmarks, evident by similar transcriptomic features in malignant cells [1, 2]. Such shared features point to the existence of common mechanisms underlying the development and progression of tumor malignancy. Conventional methods of bulk tissue analysis including RNA sequencing (RNA-seq) have emerged as powerful tools for characterizing molecular signatures across cancer tissues [1]. However, bulk samples have limitations for capturing tumor-intrinsic features due to the presence of non-tumor components, such as infiltrating immune or stromal cells in the tumor microenvironment and tumor heterogeneity [3, 4]. Single-cell transcriptome analysis, which would directly address this limitation, is a potential alternative method. It is crucial to understand cell type-specific gene expression because bulk samples sometimes mislead transcriptomic features in cancer due to low tumor purity and heterogeneity [1, 5].

Another important application of scRNA-seq would be stratification of heterogeneous malignant cells; however, meta-analysis of malignant cells across patients exhibits a high degree of patient-specific clustering, which is likely attributable to the extensive genomic heterogeneity and background variability within and across individual tumors [3, 4, 6–9]. Germline and somatic mutations contribute to the genomic background and heterogeneity, respectively, in the meta-analysis of malignant cells across tumor samples. Due to such technical and biological variability, malignant cells have not been fully investigated at the single-cell level in previous studies [3, 4, 6–9]. To identify major drivers of tumor development and progression, it is crucial to understand shared tumor-intrinsic gene expression programs beyond the genomic heterogeneity in tumor cells.

In the meta-analysis of single-cell data, principal component analysis (PCA) utilizing highly variable genes (HVGs) often captures non-biological features due to the high level of noise, such as technical biases as well as outlier samples [10]. Moreover, detection of biological markers from such PCA results suffers from inter- and intra-tumor genomic heterogeneity. Although genomic heterogeneity greatly impacts transcriptomes [2, 11], most transcriptomic changes are caused by passenger mutations rather than drivers, often missing biomarkers defining malignant cell states [12]. Hence, the meta-analysis of malignant cells from multiple batches requires appropriate integration of malignant cells by reducing noise and transcriptomic heterogeneity related to passenger mutations. Current batch correction methods can also adjust for such a batch effect in meta-analysis, but most methods focus on technical variability across batches or groups and single-cell data with multiple non-identical cell types [13–16].

Recently, consensus transcriptional programs expressed in malignant cells were reported using cancer cell lines and patients [17–19]. These studies described comprehensive malignant states ranging from well-known cancer hallmarks, such as proliferation and immune responses, to cancer-specific programs using non-negative matrix factorization (NMF) clustering analysis. Although these studies define malignant states at high resolution, NMF analysis carries out repetitive analyses and parameter tuning steps, which are time consuming. A much simpler way to define malignant cell programs is needed that can easily be integrated into existing pipelines and downstream analyses, and would accelerate studies on malignant cells using scRNA-seq.

**Figure 1:**
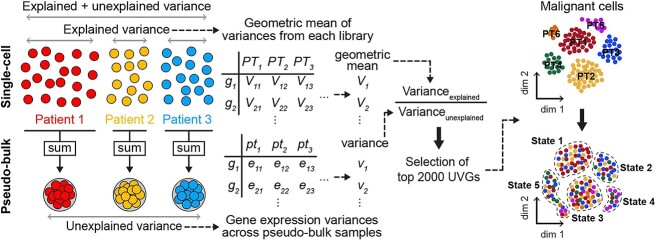
Definition of UVGs. Schematic workflow demonstrating normalization of gene expression variances to select top UVGs.

Here, we introduce a new feature selection method to extract universally variable genes (UVGs) by normalizing gene expression variances within and between individual tumors, which allows us to reduce patient-specific clusters and to discover shared transcriptomic features underlying individual tumors with different genomic backgrounds (Fig. 1). By analyzing malignant cells using UVGs, we identified several well-known cancer hallmarks, such as proliferative or inflammatory signatures, along with cancer-specific programs. We applied UVGs to various cancer types and scRNA-seq platforms and successfully minimized the formation or patient-specific clusters, demonstrating its versatile usability. Our study provides a framework to explore consensus malignant signatures found in patients with different genomic backgrounds.

## RESULTS

### UVGs describe common features across malignant cells from patients with different genomic background

We noted that the top ranked HVGs selected in the original single-cell analysis mostly originated from a single sample (or patient), which led to the formation of patient-specific clusters (Supplementary Fig. 1A). To understand the origin of these HVGs, we inferred copy number alterations (CNAs) in malignant cells and performed differentially expressed gene (DEG) analysis between patients (Supplementary Fig. 1B). Notably, about 50.70% of 2000 HVGs were also found to be DEGs (${X}^2$ test, log *P* = −1288.60), and the number of DEGs across chromosomes was well reflected by patient-specific CNAs, suggesting that inter-tumoral heterogeneity at the genomic level would be a main attribute of bias in HVGs and patient-specific clusters (Supplementary Fig. 1C, D). For example, tumor cells from patient SMC21 showed arm-level copy number gains of chromosomes 7, 8 and 20, and the majority of DEGs in these chromosomes were associated with these passenger alterations. The proportion of patient-specific DEGs that were CNA-associated ranged from 22.62 to 39.24% (Supplementary Fig. 1E, 30.16% on average).

To systematically exclude patient-specific marker genes, we devised a strategy extracting commonly variable features across multiple patients with different genomic features. In contrast to HVGs, these universal features, UVGs, can reflect transcriptomic states shared between individuals yet previously hidden by genetic differences. To determine UVGs, we normalized gene expression variance between individual tumors using within-sample variance for a given feature. UVGs were identified through the following procedure (Fig. 1): (i) for each gene, the variance was calculated in each library, separately, and summarized as a geometric mean; (ii) for each library, pseudo-bulk data were produced by aggregating gene counts; (iii) the variance was calculated for the pseudo-bulk data for each gene; and (iv) genes were re-evaluated based on the ratio of these variances to select the top-ranked 2000 UVGs. To validate whether UVGs can normalize inter-sample variability, we first simulated five scRNA-seq samples (patients), each with 1000 cells and 20000 features using SymSim (Supplementary Fig. 2A) [20]. When HVGs were used for clustering, cells were clustered by sample; however, all samples were clustered together when UVGs were used for clustering analysis (Supplementary Fig. 2B). Clustering using UVGs dramatically reduced the number of sample-specific clusters, compared with that obtained with default analysis using HVGs in simulated data with different numbers of samples, cells and features, demonstrating potential applicability to normalize inter-sample variability (Supplementary Fig. 2C–E).

### Malignant cells clustered by UVGs identify signatures of aggressive cancer cells

**Figure 2:**
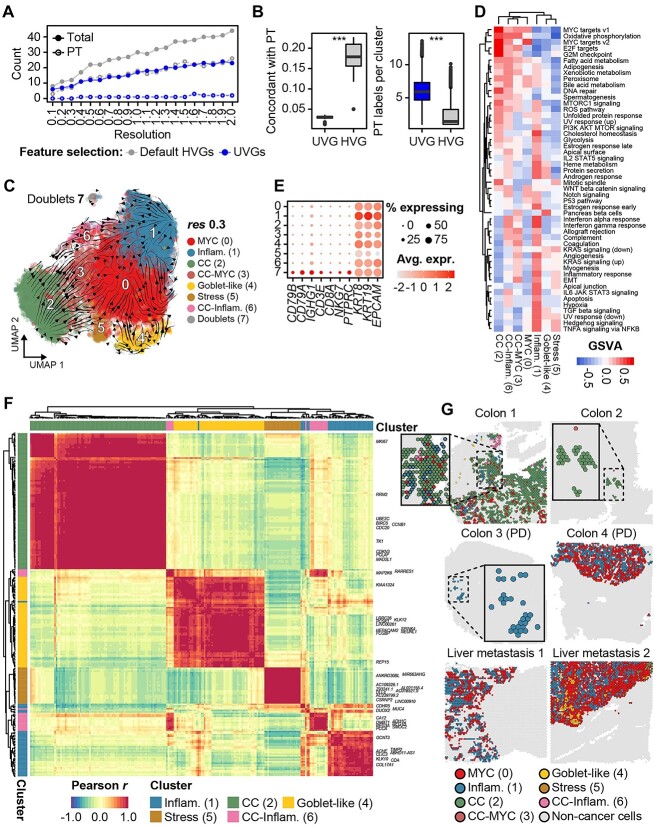
Normalized HVGs capture malignant cell states across patients. (**A**) Counts of total and patient-specific (PT) clusters. (**B**) Co-occurrence of patient and cluster labels determined using the adjusted Rand index (left), and mixing of patient labels per cluster using the local inverse Simpson’s index (right). (**C**) RNA velocity on a UMAP projection of malignant clusters. (**D**) Clustering of malignant cells using GSVA scores of hallmark gene sets. (**E**) Identification of a minor cancer-immune doublet cluster. (**F**) Correlation of expression of significant marker genes. The top 10 markers for each malignant cell state are labeled. (**G**) Annotation of malignant cell states in primary and metastatic colon cancers.

Next, we applied UVGs to 17334 malignant cells from Korean colon cancer scRNA-seq data and retrieved eight malignant cell clusters identified at resolution 0.3, where the number of patient-specific clusters was minimized (Fig. 2A, B and Supplementary Fig. 3). The expression level of UVGs was less specific to a certain sample, and all clusters consisted of cells from different patients, except that cluster 5 exhibited enrichment of cells from patient SMC23 (Supplementary Fig. 4A, B). The malignant cells from SMC23 were clustered by themselves at higher clustering resolutions (Supplementary Fig. 3) Uniform manifold approximation and projection (UMAP) and RNA velocity analyses predicted transitions in malignant cell states (Fig. 2C). Malignant states were annotated based on gene set variation analysis (GSVA) [21] of hallmark gene sets, expression of marker genes, transcription factors [22] and the literature [17, 19] (Fig. 2D–F, Supplementary Fig. 4C–E and Supplementary Table 1). Cluster 7 was excluded from the analysis because it consisted of immune-malignant doublets and displayed significantly higher numbers of UMIs and genes than other clusters (Fig. 2E and Supplementary Fig. 4F). The top 3 prevalent clusters were specifically enriched with genes related to MYC signaling (cluster 0), the inflammatory response (cluster 1) and the cell cycle (cluster 2), and the remaining cells represented intermediate states between the cell cycle and MYC signaling (cluster 3) or inflammation (cluster 6) (Fig. 2C and Supplementary Fig. 4G). Cell cycle cluster [2] transitioned to inflammatory cluster [1] through intermediate cluster [6], whereas inflammatory cluster [1] was interconnected to MYC cluster (0) (Fig. 2C). Similar transitions in malignant cell states were recapitulated when only UVGs were used to infer RNA velocity, demonstrating that UVGs represent transcriptional changes in malignant cells (Supplementary Fig. 4H). Cluster 4 was predicted to be a goblet cell-like cluster because it was enriched with tumor suppressors and developmental transcription factors for intestinal cells, such as SPDEF [23] or POU2F3 [24], and goblet cell markers (Supplementary Fig. 4C, D). Inflammatory and cell cycle states (clusters 1 and 2) have been well described in previous studies; however, other key transcriptomic hallmarks, such as MYC signaling (cluster 0) or goblet-like (cluster 4), were only identified using our UVG method (Supplementary Fig. 4E). Lastly, we clustered the CNA profile based on the malignant cell clusters; however, we did not find a strong association between the clusters and CNA profiles, indicating that the genomic heterogeneity is likely to be passengers that poorly reflect malignant cell states and their signatures, as reported in previous studies (Supplementary Fig. 4I) [12, 17].

To explore how the resulting malignant states are spatially distributed in a tissue space, we analyzed spatial transcriptomics data from primary and metastatic colon cancers [25] (Fig. 2G and Supplementary Fig. 5A, B). Malignant cells with MYC, inflammation, and cell cycle signatures were predominantly identified in spatial data from both primary and metastatic tumors. Moreover, we found huge differences between malignant cell signatures according to disease and treatment states. Tumor cells with a cell cycle signature were prevalent in treatment-naive patients, whereas those with MYC/inflammatory signatures were dominant in progressive disease (PD) states after neoadjuvant chemotherapy or liver metastasis (Fig. 2G). Goblet-like cells [4] were localized with epithelial cells (gray) in treatment-naive cancer (Colon 1) but also co-localized with malignant cells with the MYC signature in metastatic cancer (Liver metastasis 2). We then wondered about the association of certain malignant states with clinical outcomes. Kaplan–Meier survival analysis was performed with two patient groups separated by the weighted expression of signature genes. Colon cancer patients with enriched inflammation [1], cell cycle [2] or goblet-like [4] malignant signatures showed poor clinical outcomes (Supplementary Fig. 5C; *P* < 3.48 × 10^−5^, *P* < 2.67 × 10^−6^ and *P* < 3.47 × 10^−4^, respectively). Altogether, UVGs describe shared transcriptomic features expressed in malignant cells and identify aggressive signatures which are clinically implicated in colon cancer.

### Comparative analysis with batch correction methods

Although patient-specific clustering is likely originated from inter-tumor heterogeneity, such as CNA or other genetic features, single-cell data integration methods, such as Seurat integration [16], Harmony [14], scanorama [13] and scVI [15], could also provide a similar function that reduces certain batch effects and sample-specific biases. We thus compared our malignant cell signatures with those defined by other benchmarking methods. Although these methods could successfully integrate malignant cells from different samples, we noticed that utilizing UVGs showed advantages in some respects (Supplementary Fig. 6A, B). First, in contrast to UVGs (Fig. 2E), none of these methods discriminated cancer-immune doublets from genuine malignant cells (Supplementary Fig. 6C). Second, transcriptomic features of clusters were mixed between clusters. For example, clusters integrated by Harmony showed more admixture of inflammatory and proliferative states (Supplementary Fig. 6E, F). Marker genes for each cluster were much less specific in Seurat integration and were comparable between Harmony and UVG clusters (Supplementary Fig. 6D, top). We determined confident markers for a cluster as DEGs with background expression in less than 25% of cells and found that UVG clusters showed a higher proportion of expressing cells (Supplementary Fig. 6D, bottom). When we further compared clusters defined by Harmony and UVGs, the number of significant DEGs were depleted in large clusters, such as clusters 2, 3 or 4, and biased to a few clusters located in the extremities of the UMAP plot (clusters 1, 5 or 8), whereas markers of UVG clusters showed higher log_2_ fold-change and comparable *Q*-values (Supplementary Fig. 6g, h). Proliferative and inflammatory states were consistently found using Harmony and highly correlated with corresponding states defined by UVGs, suggesting that these programs are fundamental features of colon cancer cells; however, clinical associations were less significant than UVG-defined states (Supplementary Figs 5C and 6I, J). In addition, only UVG clustering detected a clinically relevant goblet-like cluster (Supplementary Fig. 5C). In summary, our novel variable gene selection method using normalized variance minimized the formation of patient-specific clusters as well as identified clinically relevant transcriptome signatures in malignant cells.

### Pan-cancer analysis reveals basal transcriptomic signatures of malignant cells

**Figure 3:**
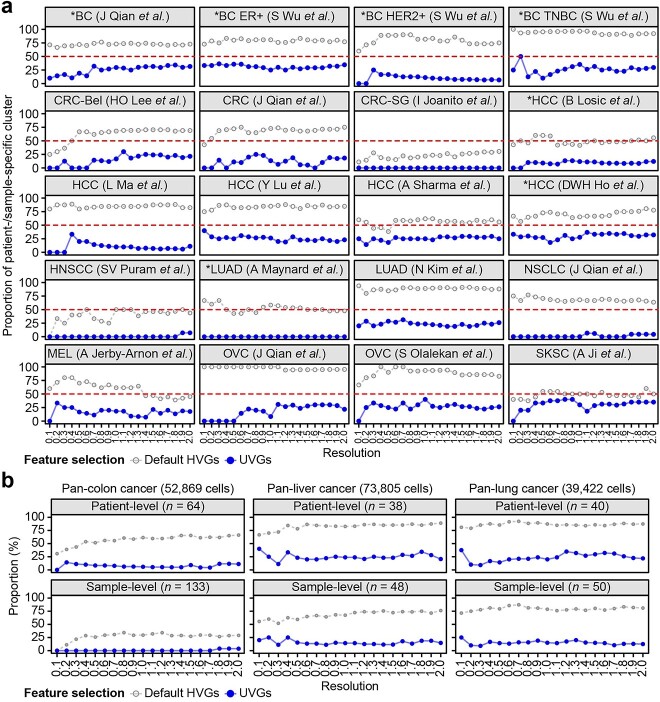
Clustering of malignant cells in public cancer scRNA-seq datasets. (**A**) Proportions of patient-specific clusters in 20 scRNA-seq datasets (eight cancer types) from 15 studies. The references for the datasets are summarized in Supplementary Table 2. (**B**) Proportions of patient/library-specific clusters in colon, liver and lung cancers. Empty dots: default HVGs, filled dots: UVGs. *PT clusters were defined as clusters in which ≥75% of cells were derived from a single sample.

**Figure 4:**
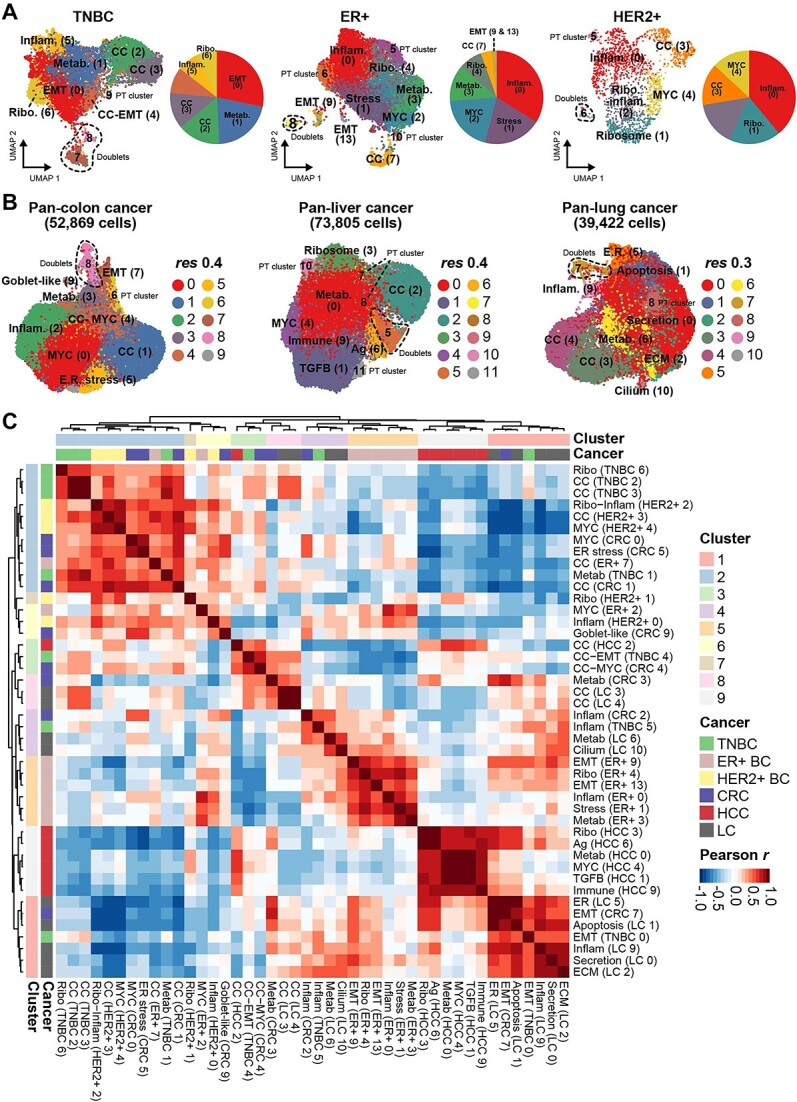
Malignant cell states shared in pan-cancer datasets. (**A**) UMAP plots of malignant states in triple-negative (TNBC), ER+ and HER2+ breast cancer subtypes. (**B**) UMAP plots of malignant states in pan-colon (left), pan-liver (middle) and pan-lung (right) cancers. (**C**) Clustering of Pearson correlation coefficients of three pan-cancer clusters using GSVA scores.

We then evaluated whether our method could be applied to other cancer types and sequencing platforms (Supplementary Table 2). Twenty single-cell datasets from eight cancer types were analyzed and the proportion of patient-specific clusters was found to be 26.55% on average using UVGs, much lower than those determined using default HVGs (76.62% on average; Fig. 3A). The reduction in patient-specific clusters allowed us to compare common transcriptomic signatures across cancer subtypes. For example, three breast cancer subtypes were clustered by shared transcriptomic features, but characteristics of different subtypes, triple-negative breast cancer (TNBC), ER+ and HER2+, were preserved (Fig. 4A and Supplementary Figs 7–9) [26]. In general, MYC or proliferative states were anti-correlated with inflammatory or invasive states [27]; however, an invasive program was highly enriched in the TNBC subtype but depleted in ER+ breast cancer along with proliferative states (Fig. 4A, EMT and CC) [28]. To compare overall malignant states in breast cancer, we correlated enrichment scores for hallmark and an additional 125 gene sets defined in breast cancer and found a high correlation between similar transcriptional states across subtypes (Supplementary Figs 7–9F and 9G) [29].

Finally, we expanded our evaluation to large-scale pan-cancer datasets from independent studies. Briefly, 52869 colon cancer cells, 73805 liver cancer cells and 39422 lung cancer cells from multiple data sources were integrated and variable genes were re-evaluated with UVGs. Proportions of patient−/sample-specific clusters were greatly reduced in all three datasets (Fig. 3B, 7.54 ~ 24.88% on average using UVGs compared with 56.87 ~ 86.26% using HVGs at the patient level). Transcriptomic features were compared after excluding patient-specific clusters and *PTPRC*-expressing doublets (Fig. 4B and Supplementary Fig. 10A–C). Signatures in colon cancer were recapitulated in pan-colon clusters (Supplementary Fig. 10G), whereas similar yet distinct combinations of signatures were found in other cancers (Supplementary Fig. 10D–F). For example, an antigen processing signature [6] in pan-liver cancer included expression of cell cycle along with inflammatory-related genes, whereas a cilium signature [10] was only identified in pan-lung cancer. Lastly, we performed hierarchical clustering and PCA of signature enrichment scores to define pan-malignant states (Fig. 4C; Supplementary Fig. 11A, B). Although clusters from ER+ breast and liver cancers were largely correlated by cancer type (Fig. 4C, cluster 5 and 9) due to specific expression of ER-responsive genes and metabolic pathways, respectively (Supplementary Fig. 11A), we found several recurring clusters across four pan-cancers including invasion [1], proliferation/MYC [2] and inflammation [4] (Fig. 4C). We gathered all UVGs found in pan-cancer datasets and summarized them using the number of identified datasets. Approximately 31.12% of total UVGs (2563 of 8237 unique genes) were found in at least two different datasets (Supplementary Fig. 11C and Supplementary Table 3). To identify molecular features of shared UVGs in different cancers, we searched for GO terms using 863 genes found in at least three different datasets (Supplementary Fig. 11C). Consistent with the results of clustering analysis, we found proliferation-related GO terms, such as ‘Mitotic nuclear division’ or ‘Positive regulation of cell cycle process’, along with several gene sets associated with inflammatory states (Supplementary Fig. 11D). Taken together, we defined underlying molecular signatures shared in tumorigenesis using UVGs and our approach can detect these key features without patient bias.

## DISCUSSION

Recent studies used iterative NMF and correlation analyses to cluster transcriptomic programs in cancer cell lines and patients [17–19]. These pioneering works defined recurrent transcriptomic signatures expressed by malignant cells; however, such iterative analysis is computationally intensive with atlas-level datasets and requires expertise in bioinformatics. Compared with NMF-based clustering, feature selection with UVGs would be useful because it can be seamlessly integrated with existing analytic pipelines and is easily subjected to downstream analyses, such as DEG or gene regulatory network analyses. Using UVGs, we defined well-known cancer hallmarks including inflammation and cell cycle signatures, along with MYC signaling and goblet-like malignant states that were not reported in previous transcriptomic programs. Our results also highlight a transcriptomic trajectory from proliferative to inflammatory/MYC transcriptomic states in resistant cancers.

Understanding these malignant states will be crucial for controlling aggressive diseases, such as drug-resistant or metastatic cancers. Detection of genetic features that can be used as druggable molecular targets, such as mutations or gene fusions, is vital in cancer therapy. We recognize the importance of patient-specific genetic features in precision medicine; however, tumor-intrinsic transcriptomic programs will also provide novel insight into how malignant cells transform and interact with the tumor microenvironment.

A weak association between malignant states and genomic heterogeneity suggested that the transcriptomic heterogeneity of malignant cells mostly resulted from passenger mutations rather than drivers (Supplementary Fig. 4I) [17]. Such inter-tumor heterogeneity can produce patient-specific signatures and hinder the detection of malignant signatures at the single-cell level. Patient-specific signatures were systemically normalized using UVGs due to a lack of true genomic information in scRNA-seq data. Although UVGs allowed us to detect transcriptional programs in cancers related to tumor progression and hallmarks, the clinical relevance of which were validated using independent TCGA bulk RNA-seq datasets as did in the previous analysis [18].

We also noted that increasing the number of replicates for each patient is significant for minimizing patient-specific clusters, possibly by the incorporation of intra-tumoral heterogeneity (Supplementary Fig. 12). However, increasing the number of biological replicates can come at a cost to the size of the population, which is also an important factor to be considered in a study. Given these limitations, UVG analysis would be a smart choice for unveiling basal transcriptional programs in malignant cells. We anticipate that high-throughput multi-omics single-cell platforms will provide valuable insight regarding associations between transcriptomes and genetic features in future research. Further improvements in variable gene evaluation, obtained by employing sophisticated statistical models integrating CNAs and patient DEGs or multi-omics analysis with genomics or epigenomics data, would, in turn, improve the resolution of malignant cell states.

## CONCLUSION

In this study, we presented UVGs as a novel feature selection strategy for clustering malignant cells from different patients by normalizing both within- and between-sample heterogeneity. The within-sample variability of malignant cells was mostly attributed to tumor heterogeneity and technical variability, while the between-sample variability resulted from genetic background variation at the single-cell level, as implicated in HVGs. In contrast to HVGs, application of UVGs to meta-analysis of scRNA-seq datasets defined basic transcriptomic programs expressed across different patients and cancer types. This method provides a convenient solution for the meta-analysis of large-scale single-cell datasets from multiple sources, patients and cancer types.

## METHODS

### Analysis of colon cancer scRNA-seq data

Datasets from 23 Korean colon cancer patients were analyzed with cell annotation labels from the original publication by Lee *et al.* [3] A total of 63689 barcodes with cell type information were extracted from the raw UMI count matrix. After ‘Unknown’ cell types were removed, 61508 cells were analyzed using Seurat v3.0 [16] in R (version 3.5.1). Briefly, UMI counts were normalized, log-transformed and scaled, and 99 principal components (PCs) were used to explain the variability of the scaled UMI counts across cells. Sequencing libraries were integrated by normalizing for batch effect using Harmony [14]. Marker genes were identified using *FindAllMarkers()* with *test.use = ‘MAST’* [31], *min.pct = 0.25*, and *only.pos = TRUE* parameters. To examine patient/library-specific clustering patterns, malignant cells were extracted and *PTPRC* (CD45)-expressing immune cells (122 cells) and a minor library from SMC5 (13 cells) were removed (*n* = 17334).

RNA velocity was inferred using scVelo [32] in Python (version 3.7.12). First, loom files containing spliced and unspliced counts were produced for each library using the velocyto package [33]. Next, loom files from tumor samples were aggregated and only malignant cells were extracted. RNA velocity was then inferred on malignant cells in dynamical mode with the following parameters in the preprocess steps:

*scvelo.pp.filter_and_normalize(loom_object, min_shared_counts = 20, n_top_genes = 2000)*

*scvelo.pp.moments(loom_object, n_pcs = 50, n_neighbors = 30)*

*scvelo.tl.recover_dynamics(loom_object)*

*scvelo.tl.velocity(loom_object, mode = ‘dynamical’)*

*scvelo.tl.velocity_graph(loom_object)*

### Normalization of gene expression variance

We hypothesized that gene expression variances in scRNA-seq data could be defined at three different levels: (i) all cells from different libraries or data sources could be used, and with malignant cells, this strategy would measure combined inter/intra-tumoral heterogeneity (explained + unexplained variance); (ii) sequencing libraries could be measured for gene expression variance separately to identify intra-tumoral heterogeneity (explained variance); 3) sequencing libraries could be transformed into pseudo-bulk data by aggregating all UMI counts of cells and gene expression variance could be assessed across pseudo-bulk data to identify inter-tumoral heterogeneity (unexplained variance) (Fig. 1A). We normalized the gene expression variance by dividing the geometric mean of the variance of each library by the variance of the pseudo-bulk data and defined the top 2000 ranked genes as a new set of variable genes across malignant cells, which we named UVGs. All gene expression variances were calculated on standardized expression using variance-stabilizing transformation without clipping to a maximum value using *FindVariableFeatures()* in Seurat. Briefly, the log-transformed variance and mean of gene expression levels were fitted using loess regression and the expression values were standardized using the observed mean and expected variance. Gene expression variance was calculated on the standardized expression values. These variance values are stored in the Seurat object and can be extracted using the following command:

*<object > @assays$RNA@meta.features$vst.variance.standardized*

### Simulation of scRNA-seq data using SymSim

To benchmark variable gene selection under various conditions, five virtual samples were simulated using default parameters or the top 10 scRNA-seq libraries with the highest number of malignant cells were selected to simulate scRNA-seq using SymSim [20] in R (version 3.5.1). The following conditions with different complexities were simulated using these samples and repeated five times (Supplementary Fig. 2): (i) 2, 4, 6, 8 and 10 virtual libraries each with 500 cells and 20000 features; (ii) 5 virtual libraries of 20000 features each with different numbers of cells (50 ~ 2000 cells); (iii) 5 virtual libraries each of 500 cells each with different numbers of features (2500 ~ 20000 features). Gene lengths for the simulation were randomly selected from the GENCODE v34 transcriptome annotation.

### InferCNV analysis

CNAs in malignant cells were analyzed using inferCNV (inferCNV of the Trinity CTAT Project. https://github.com/broadinstitute/inferCNV, version 1.2.1) in R (version 3.6.3). UMI counts of malignant cells and reference non-malignant cells were extracted from a Seurat object. CD8 T cells and NK cells were used as reference cell types. The gene order was determined with GENCODE v34 by summarizing the gene symbol, chromosome, and start and end positions of gene annotations. The following command lines were used to perform InferCNV analysis:

*infercnv_obj < − CreateInfercnvObject (raw_counts_matrix = <count_file>, \*

*annotations_file = <cell_annotation_file>, \*

*delim = ‘\t’, \*

*gene_order_file = <gene_order_file>, \*

*ref_group_names = c(‘CD8’, ‘NK’))*

*infercnv_obj < − infercnv::run(infercnv, \*

*cutoff = 0.1, out_dir = ‘./’, cluster_by_groups = TRUE, \*

*denoise = TRUE, HMM = TRUE)*

CNA states were extracted from the *expr.data* slot in the *17_HMM_predHMMi6.hmm_mode-samples.infercnv_obj* file in the result directory of the InferCNV analysis. The predicted CNV states were summarized into six categorical values that indicate the following: 1 = 0x, complete loss; 2 = 0.5x, loss of one copy; 3 = 1x, neutral; 4 = 1.5x, gain of one copy; 5 = 2x, gain of two copies; 6 = >2x, gain of more than two copies. To predict the overall CNA states of patient-specific DEGs, the CNA states of individual cells were summarized for each DEG. In summary, the CNA states of 4519 out of 4945 patient DEGs were predicted and the proportion of copy number gain-associated DEGs ranged from 22.62% to 39.24% across patients. An average of 30.16% (median of 30.31%) of the DEGs were related to genomic gain.

### Gene set variation analysis

GSVA [21] was performed using 50 hallmark gene sets (*h.all.v7.2.symbols.gmt* was downloaded from the MSigDB website) using R (version 3.5.1). An expression matrix and an annotation file for samples in the expression matrix were used to build an *ExpressionSet* object using the following command:

*exprSet < − ExpressionSet(as.matrix(normalized_expression_matrix), \*

*phenoData = AnnotatedDataFrame(sample_annotation_file), annotation = ‘Symbol’)*

GSVA analysis was performed using the *ExpressionSet* object with the following command and the resulting GSVA scores were visualized using pheatmap with clustering:

*es.gsva < − gsva(expr = exprSet, \*

*gset.idx.list = GeneSetCollection(object = gene_set_list), method = ‘gsva’, kcdf = ‘Gaussian’,*

*parallel.sz = 3)*

### Gene regulatory network analysis using pySCENIC

Malignant cells were transformed to pseudo-cells by aggregating UMI counts of 10 randomly selected barcodes without replacement for each cluster (1724 pseudo-cells, approximately one tenth of the original data). The resulting count matrix was transposed and used to build a loom object with cluster labels. Transcription factor activities were inferred using pySCENIC [34] (Python 3.6.13) with the following transcription factor motif enrichment databases from cisTarget: *hg19-500 bp-upstream-7species.mc8nr.feather* and *hg19-tss-centered-10 kb-7species.mc8nr.feather*. In total, 74 transcription factors were identified and AUCell scores were scaled and clustered using pheatmap with *clustering_distance_rows = ‘euclidean’*, *clustering_distance_cols = ‘euclidean’*, and *clustering method = ‘complete’* parameters in R (version 3.5.1).

### Visium spatial gene expression analysis

Raw count data and images for colon cancer and liver metastatic colon cancer samples were downloaded from http://www.cancerdiversity.asia/scCRLM^25^. The data were first normalized and analyzed using SCTransform and Seurat v4.0, respectively, in R (version 4.1.0) [35, 36]. Spots were annotated using label transfer with colon cancer scRNA-seq data produced as described in the Methods section, Analysis of colon cancer scRNA-seq data. Briefly, 45 087 cells from 23 tumor samples and 1070 epithelial cells from 10 normal samples were extracted and normalized using SCTransform, and broad cell types (epithelial, B, plasma, T, Tregs, cytotoxic T, NK, stromal, endothelial cells, macrophages, cDC, fibroblasts and seven malignant cell states) were used as a reference in the label transfer analysis. For liver metastatic colon samples, spots were first annotated at a clustering resolution of 0.6 with expression levels of well-known protein coding marker genes, whereas metastatic colon cancer cells were identified with the expression of the following lncRNAs: *SPINT1-AS1*, *LINC01133*, *AC103702*.2, *SNHG17* and *CASC19*. Malignant cell states were then predicted with the reference dataset using label transfer analysis. Metastatic liver sample 3 was excluded from the analysis in that we were unable to predict cancer cell states.

### Weighted survival analysis

Cox proportional hazard multivariate analysis was performed on TCGA colon adenocarcinoma data, and the pathologic tumor stages were significantly associated with survival rates (stage III/IV, hazard ratio = 5.3, *P* < 0.001). Clinical associations of signature genes with each malignant cluster were tested after adjusting for pathologic stages and coefficient values were extracted. Then, the sum of the log_2_-transformed gene expression level weighted by the coefficients was used as the cluster signature score. In summary, for *n* number of signature genes *g* of a malignant cluster, the following cluster score was calculated and used to stratify patient groups by quartile values:

$Cluster\kern0.17em score={\sum}_{\left(i=1\right)}^n gi\;{coefficient}^{\ast}\mathit{\log}2\left( gi\; expression+1\right)$

### Integration of scRNA-seq libraries using batch correction methods

To compare clusters determined by batch correction to malignant cell states defined using UVGs, the following four widely used batch correction tools were used: integration using Seurat [16], Harmony [14], scanorama [13] and scVI [15]. Seurat integration was performed using the default parameters. Specifically, a Seurat object was split by a patient attribute and variable features were determined using *SelectIntegrationFeatures*. These features were used to find anchors between scRNA-seq libraries using *FindIntegrationAnchors*, which were used to integrate scRNA-seq libraries using *IntegrateData*. The integrated data were analyzed using the first 30 PCs. For Harmony, *RunHarmony* was used with the *group.by.vars = ‘Library’* parameter and the following clustering analysis was performed with the first 30 PCS and *reduction = ‘harmony’*. For scanorama, mtx files were first generated for each patient in R separately, and then processed in Python. Count matrices were transformed to anndata using scanpy’s *read_10x_mtx* and stored in a list variable. The list of anndata were integrated using *scanorama.correct_scanpy* with *return_dimred = True*. The low dimension matrix integrated by scanorama was retrieved from *scanpy_object.obsm[‘X_scanorama’]* and specified for the *reduction* parameter in clustering analysis using Seurat. Similarly, the count matrix was retrieved from *scanpy_object.X.toarray()*. For scVI, the Seurat object was transformed to anndata using *convertFormat.* scvi and scanpy libraries were imported to R using the reticulate package. Anndata was subjected to *scvi$model$SCVI()* to create a generative model for scVI analysis. The model was then trained and latent representation was retrieved using *scVI_model$get_latent_representation().* The latent matrix was used for the *reduction* parameter in clustering analysis using Seurat.

### Collection of pan-cancer scRNA-seq data

The following raw or processed scRNA-seq data were downloaded from public databases: colon cancer = KUL (raw data from E-MTAB-8410) [3], SG1 and SG2 (processed data from syn26844071) [30], and Blueprint (http://blueprint.lambrechtslab.org) [37]; liver cancer = Losic *et al.* (raw data from GSE112271) [38], Ma *et al.* (GSE125449) [8], Lu *et al.* (GSE149614) [39], Sharma *et al.* (GSE156625) [40] and Ho *et al.* (SRP318499) [41]; lung cancer = Maynard *et al.* (raw data from PRJNA591860) [42], Kim *et al.* (GSE131907) [43], and Blueprint; breast cancer = Wu *et al.* (GSE176078) [26] and Blueprint. References for other cancers are available in Supplementary Table 2. Libraries having too few cells (0.1% of the total number of cells in each dataset) were excluded. The resulting datasets consisted of 52869 cells from 133 libraries or 64 patients for pan-colon cancer, 73805 cells from 48 libraries or 38 patients for pan-liver cancer, and 39422 cells from 50 libraries or 40 patients for pan-lung cancer.

### PCA and clustering of pan-cancer GSVA scores

PCA was performed on GSVA scores of 50 hallmark gene sets in R (version 3.5.1) using the *FactoMineR* package with *scale.unit = False*. From a total of 27 PCs, the top two were visualized (PC 1 = 32.19% and PC 2 = 26.80% of variance). Clusters were defined by Euclidean distance using the pheatmap function with *clustering_distance_rows = ‘euclidean’*, *clustering_distance_cols = ‘euclidean’* and *clustering method = ‘complete’* parameters in R. Trees in the heatmap analysis were extracted and eight clusters were identified using the *cutree()* function with *h = 5*. Clusters were named after representative gene sets.

**Key Points**

- Patient clusters in scRNA-seq analysis are generated by CNA-related highly variable genes.
- UVGs describe diverse transcriptomic characteristics of malignant cells, including cancer hallmarks.
- An inflammatory state defined by UVGs was clinically implicated and often localized at the tumor margin.
- UVGs identified not only common transcriptomic features across cancer types, but also cancer-specific clusters.

## Supplementary Material

Supplementary_Figure_1_bbad460

Supplementary_Figure_2_bbad460

Supplementary_Figure_3_bbad460

Supplementary_Figure_4_bbad460

Supplementary_Figure_5_bbad460

Supplementary_Figure_6_bbad460

Supplementary_Figure_7_bbad460

Supplementary_Figure_8_bbad460

Supplementary_Figure_9_bbad460

Supplementary_Figure_10_bbad460

Supplementary_Figure_11_bbad460

Supplementary_Figure_12_bbad460

Suppmentary_Table_1_bbad460

Suppmentary_Table_2_bbad460

Suppmentary_Table_3_bbad460

## Data Availability

The Visium primary and liver metastatic colon cancer [25] data and scRNA-seq data from colon [3, 30, 37] and other cancers are publicly available. References for cancer datasets are provided in Supplementary Table 2.

## CODE AVAILABILITY

In-house R and Python codes used in this study are available on GitHub (https://github.com/sangho1130/UVG). Detailed parameter settings and thresholds used in the analyses are described in the Methods. All analyses were performed using Python and R in a conda virtual environment. Detailed software versions are also described in the Methods.

## Author Biographies

**Sang-Ho Yoon** is a Post-doc fellow at Department of Life Science, College of Natural Sciences, Hanyang University, Seoul 04763, Republic of Korea. His research interests are single-cell RNA-sequencing and spatial transcriptomics data analyses.

**Jin-Wu Nam** is a professor at Hanyang University. He received a PhD in bioinformatics from Seoul National University and studied computational biology at the Whitehead Institute for Biomedical Research.
