# Supplementary figures and images for: Clustering malignant cell states using universally variable genes
Source: Brief Bioinform. 2023 Dec 11;25(1):bbad460. doi: 10.1093/bib/bbad460 (PMC10783859; doi:10.1093/bib/bbad460)

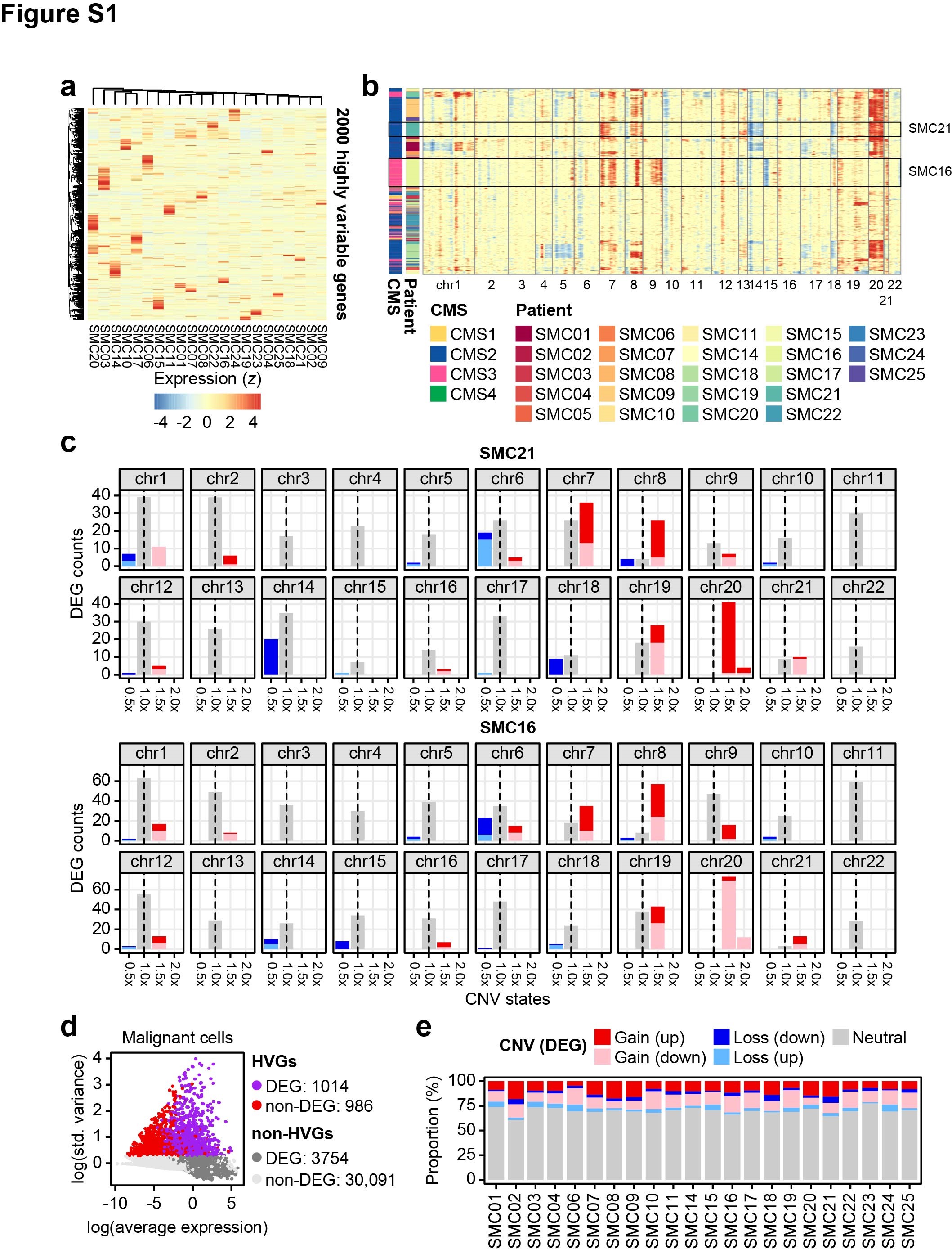

Supplement: Supplementary_Figure_1_bbad460 [file supplementary_figure_1_bbad460.jpeg]

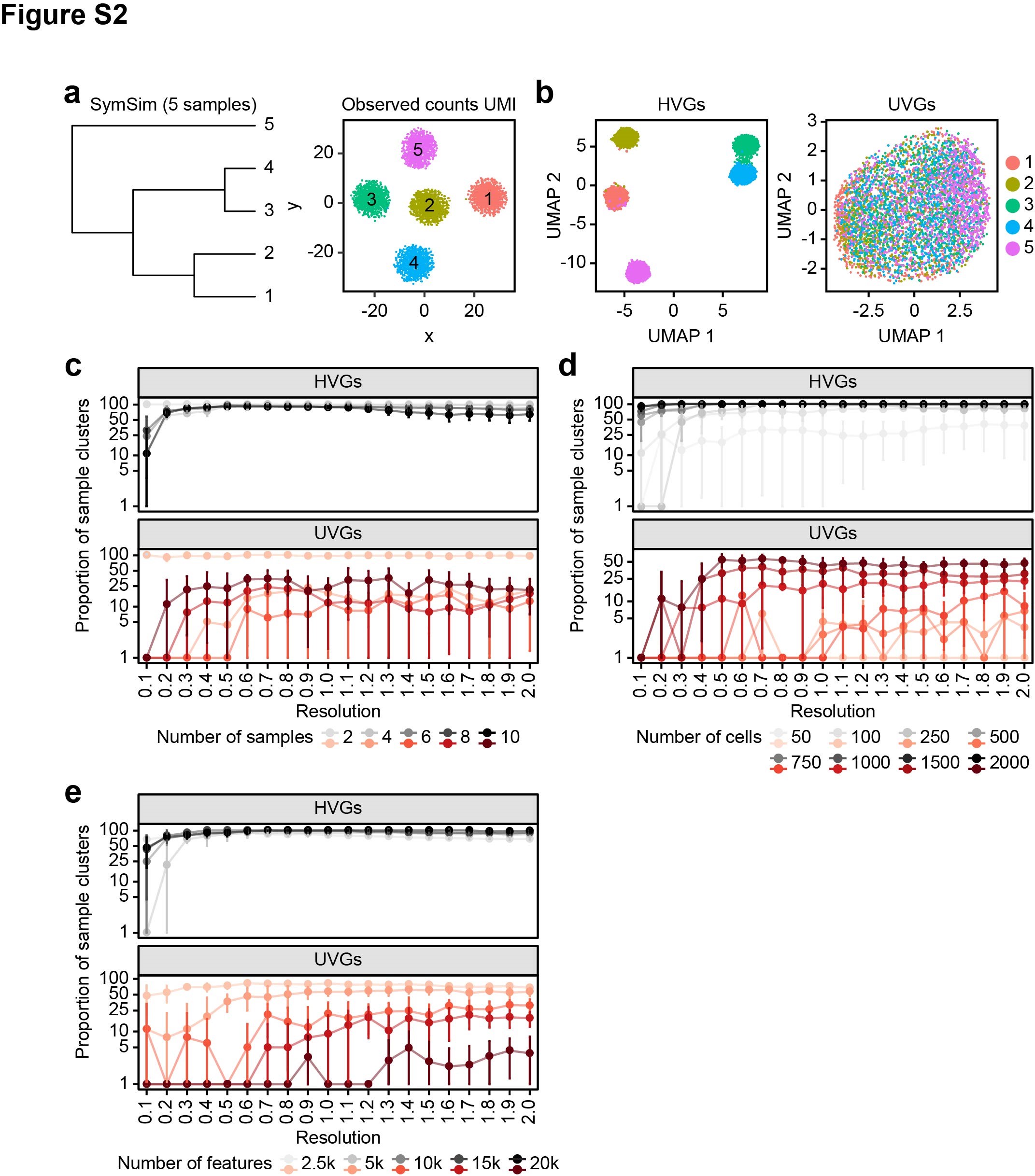

Supplement: Supplementary_Figure_2_bbad460 [file supplementary_figure_2_bbad460.jpeg]

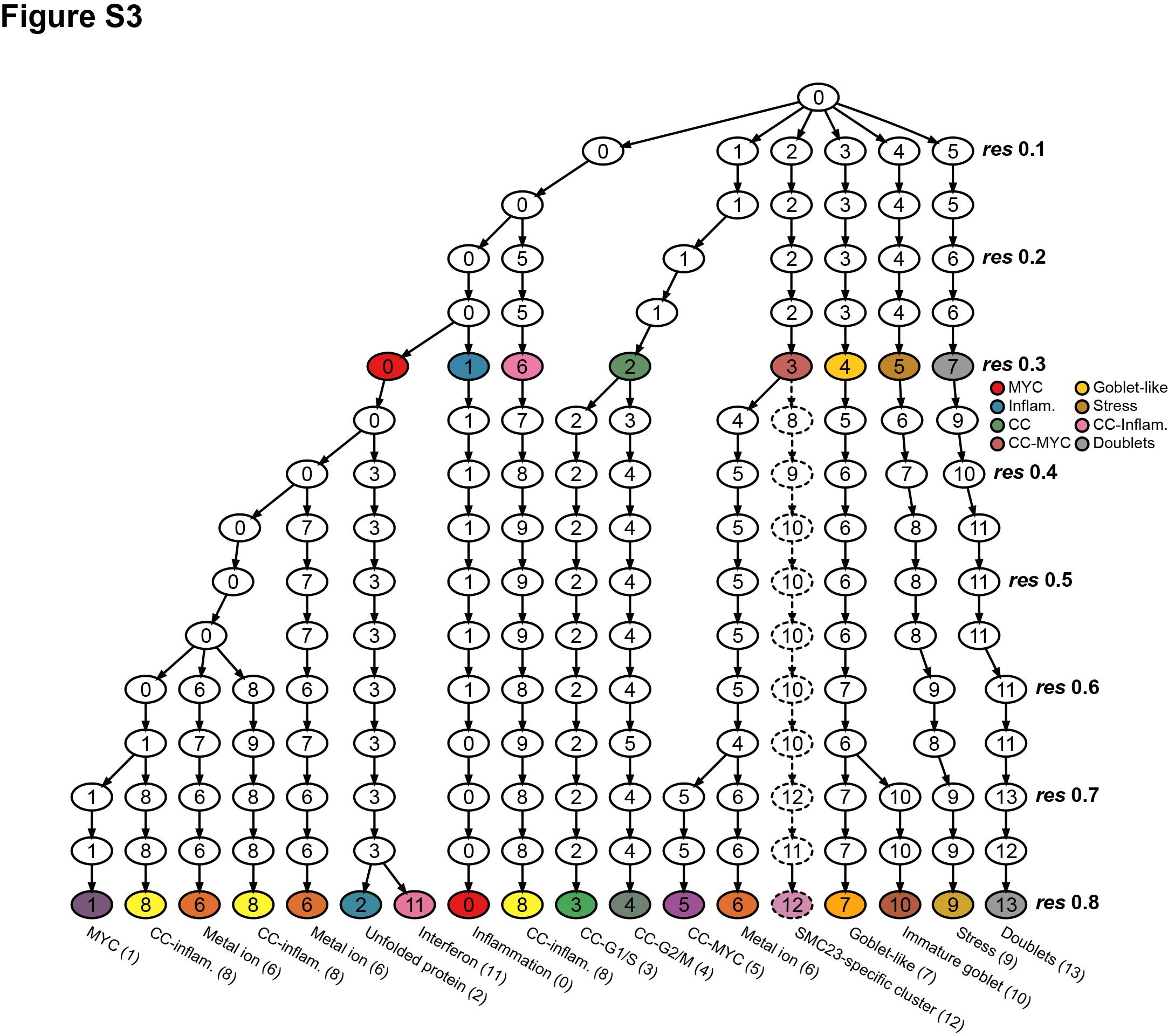

Supplement: Supplementary_Figure_3_bbad460 [file supplementary_figure_3_bbad460.jpeg]

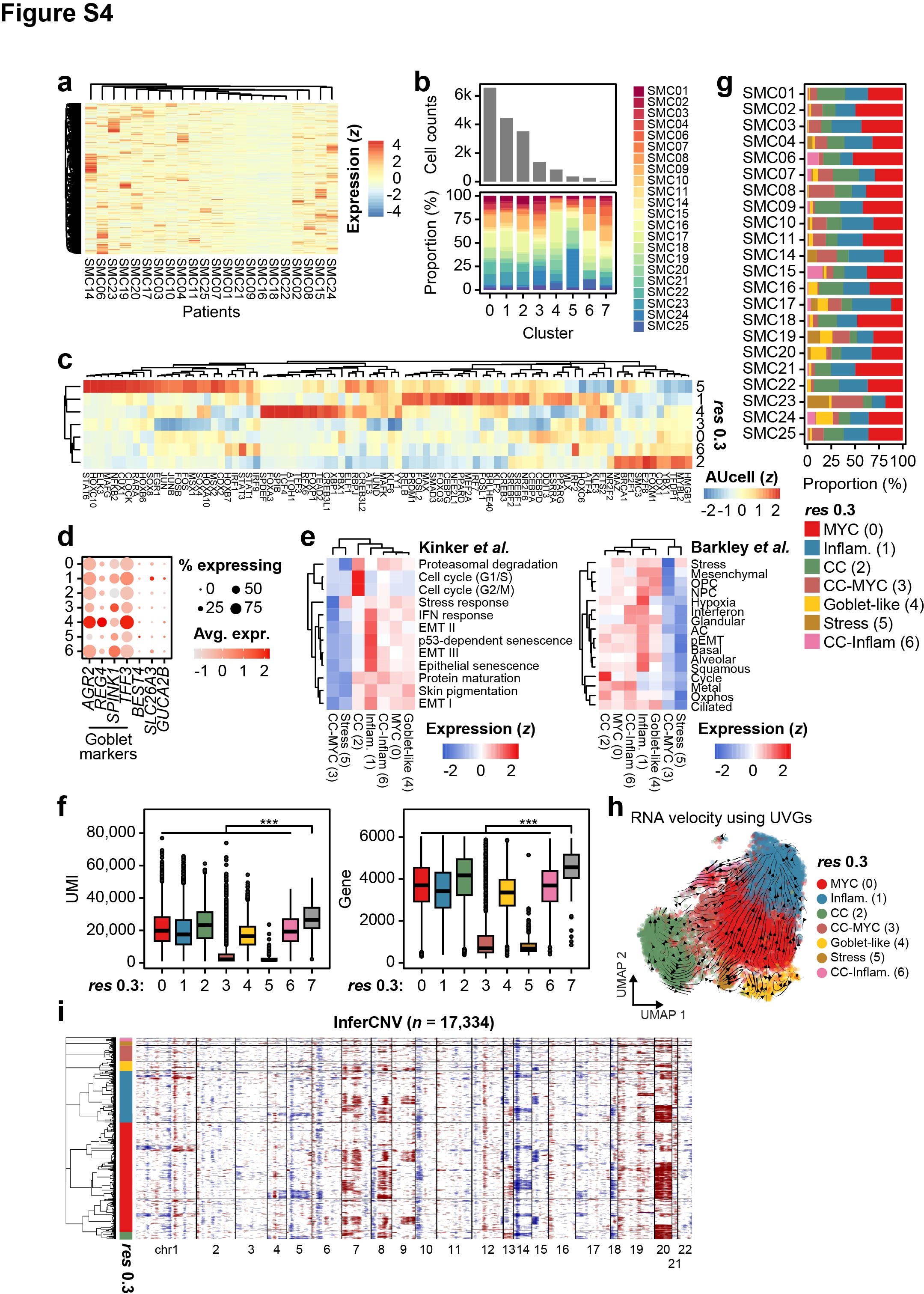

Supplement: Supplementary_Figure_4_bbad460 [file supplementary_figure_4_bbad460.jpeg]

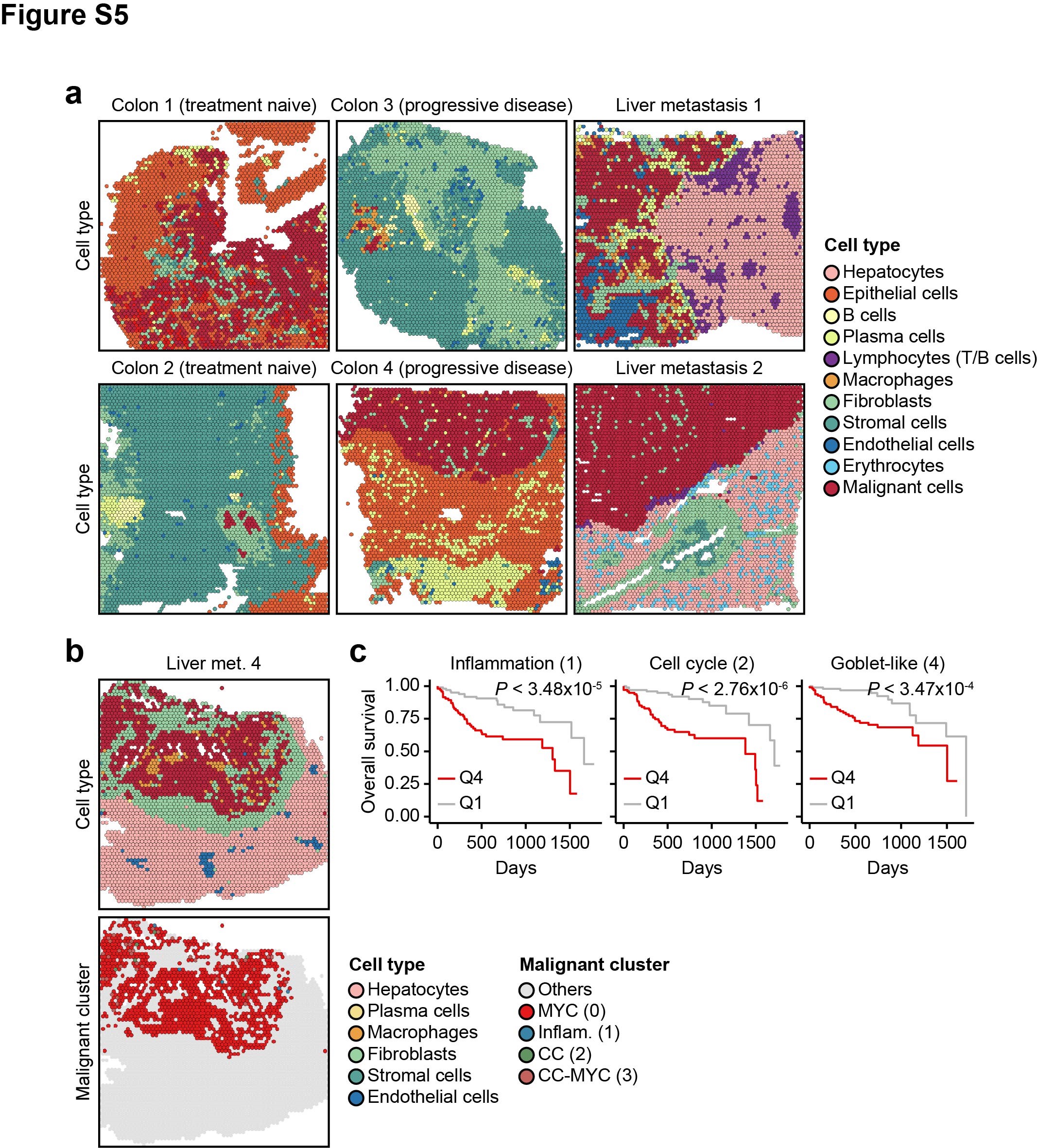

Supplement: Supplementary_Figure_5_bbad460 [file supplementary_figure_5_bbad460.jpeg]

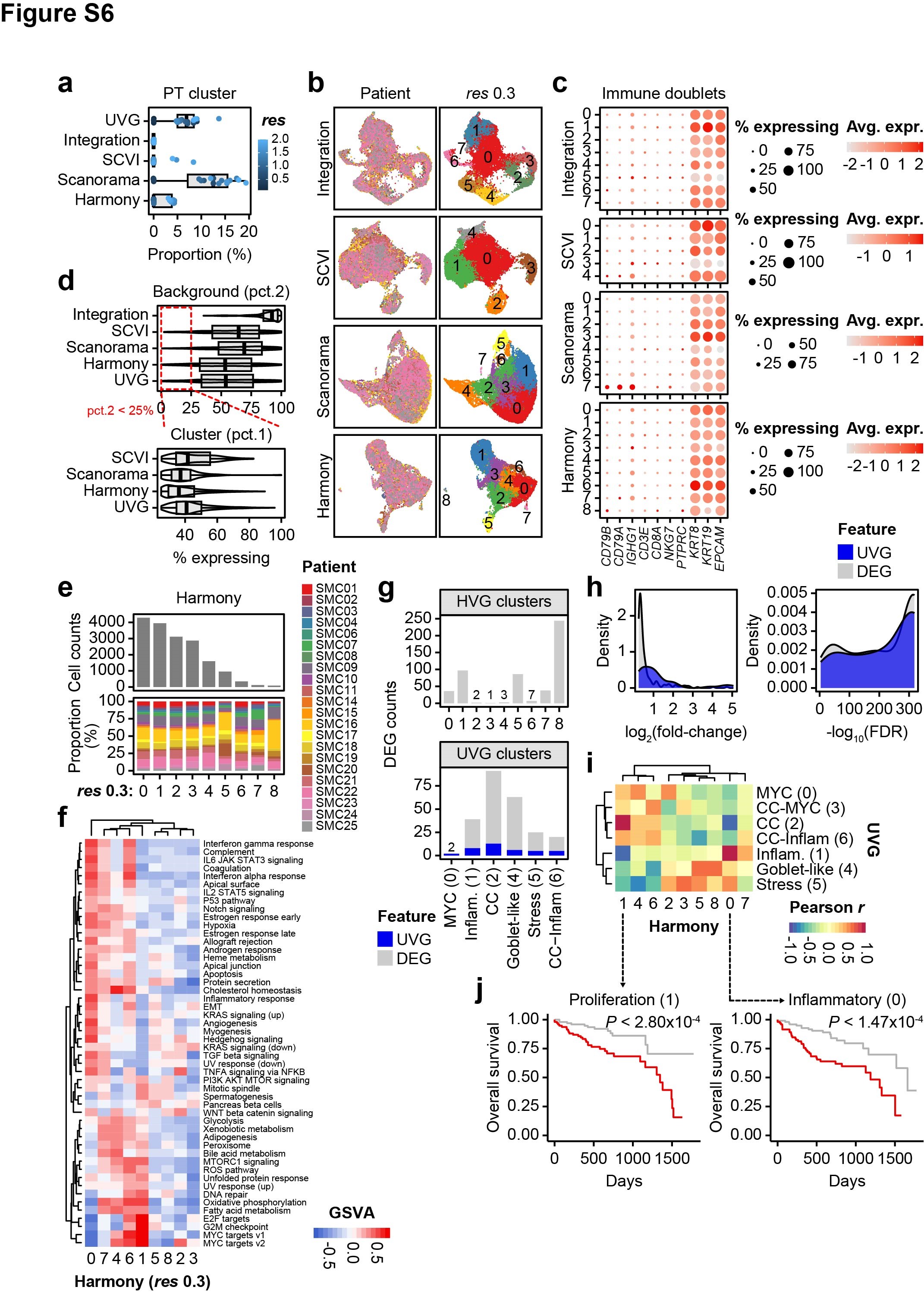

Supplement: Supplementary_Figure_6_bbad460 [file supplementary_figure_6_bbad460.jpeg]

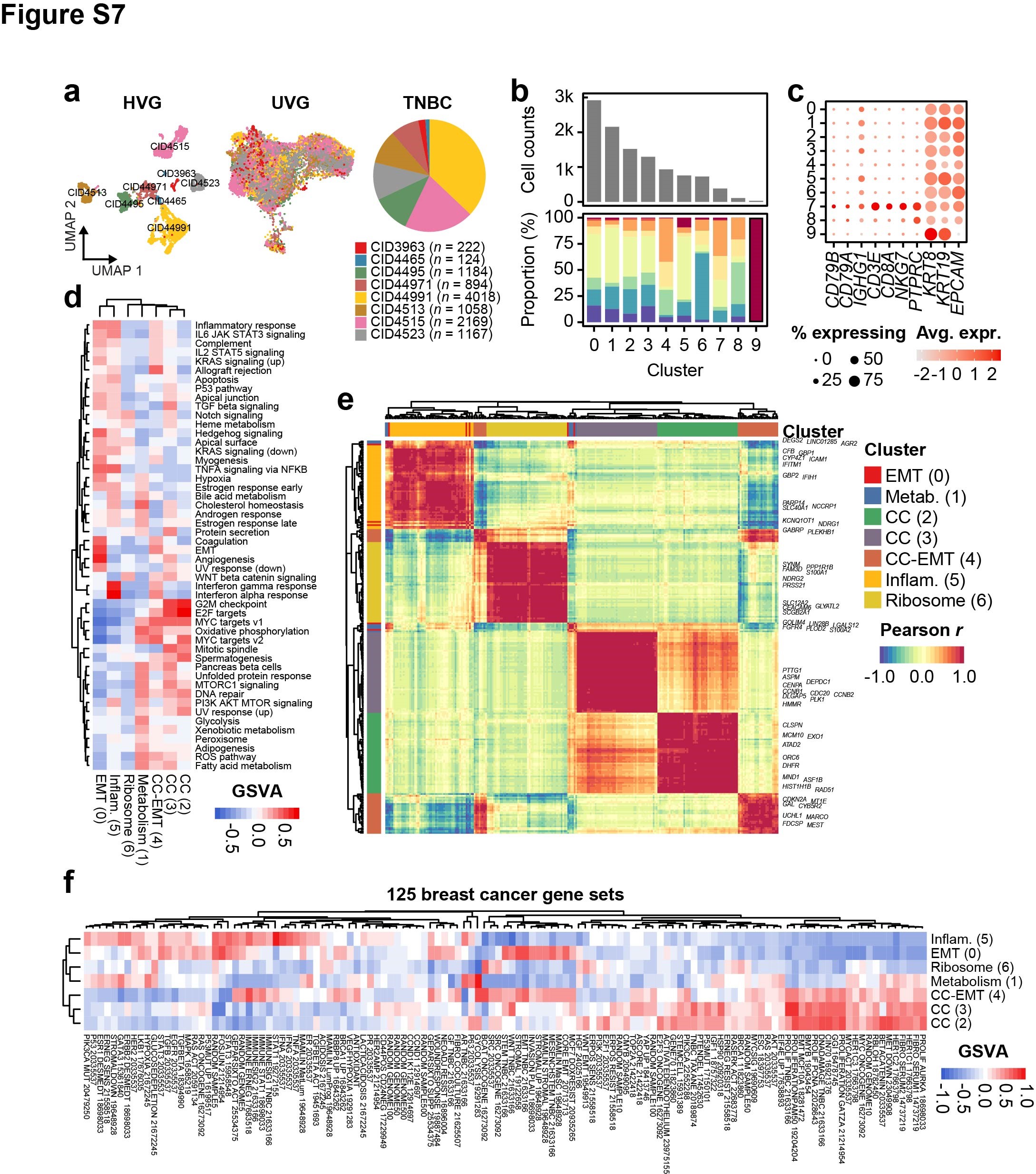

Supplement: Supplementary_Figure_7_bbad460 [file supplementary_figure_7_bbad460.jpeg]

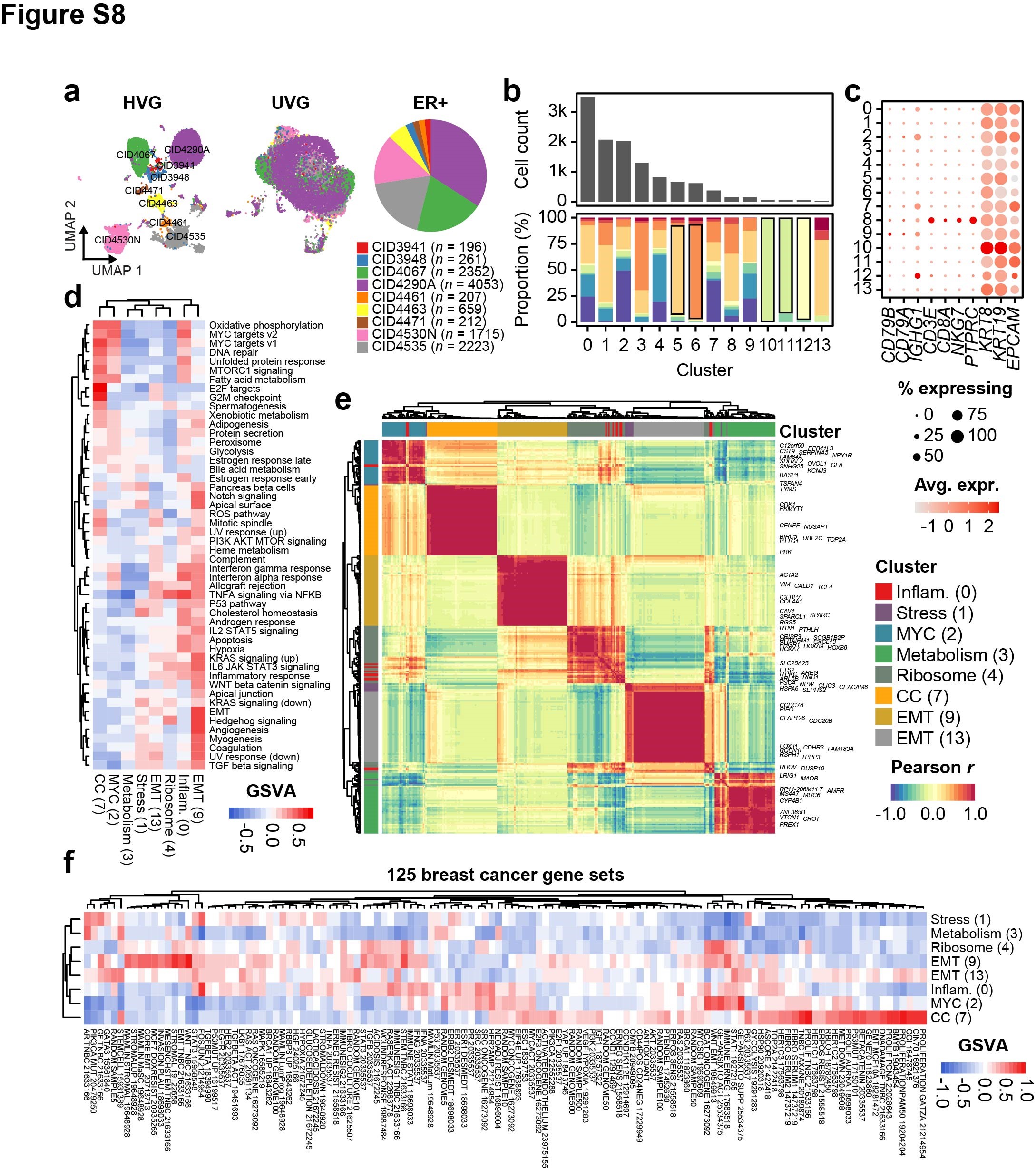

Supplement: Supplementary_Figure_8_bbad460 [file supplementary_figure_8_bbad460.jpeg]

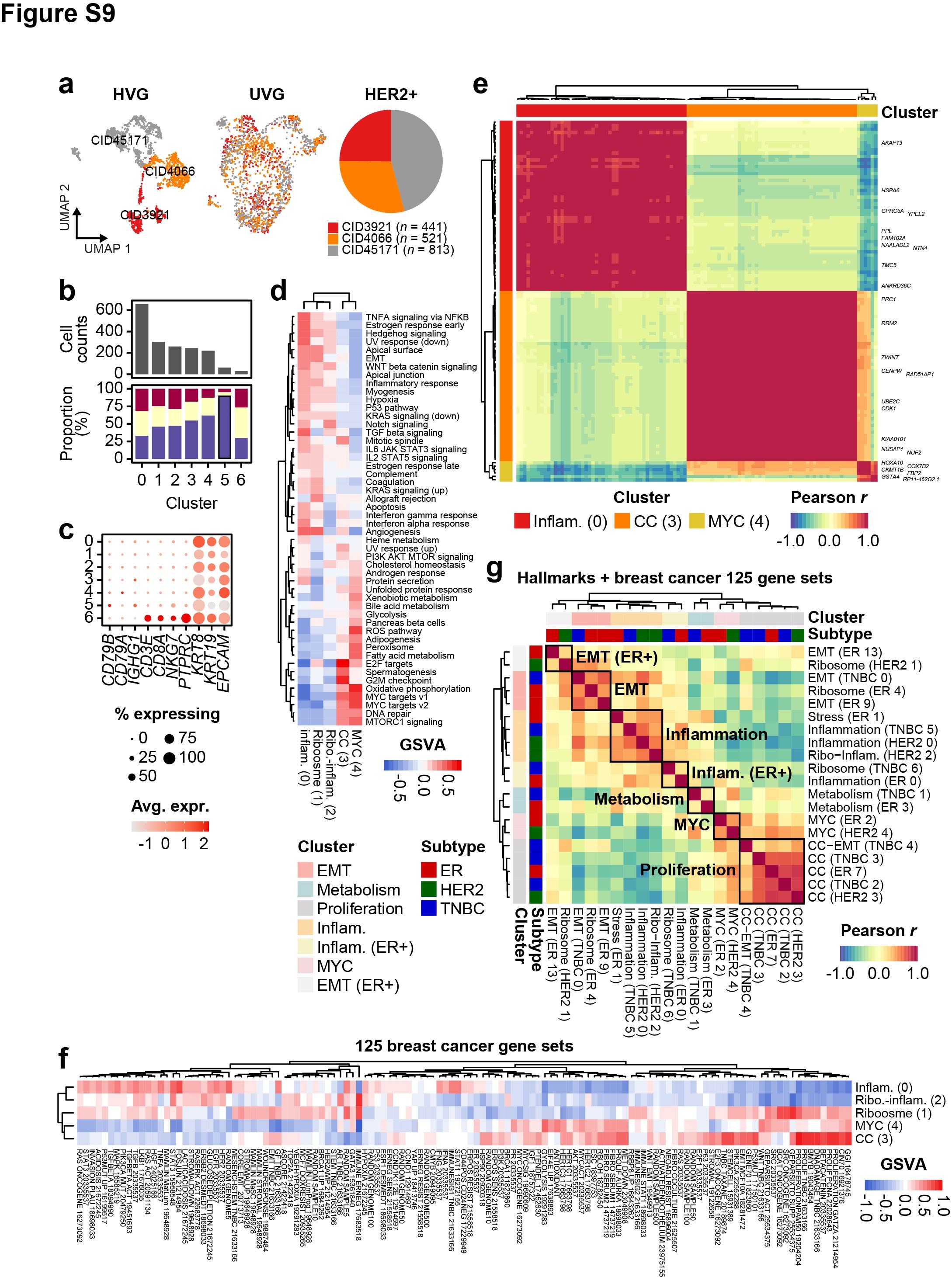

Supplement: Supplementary_Figure_9_bbad460 [file supplementary_figure_9_bbad460.jpeg]

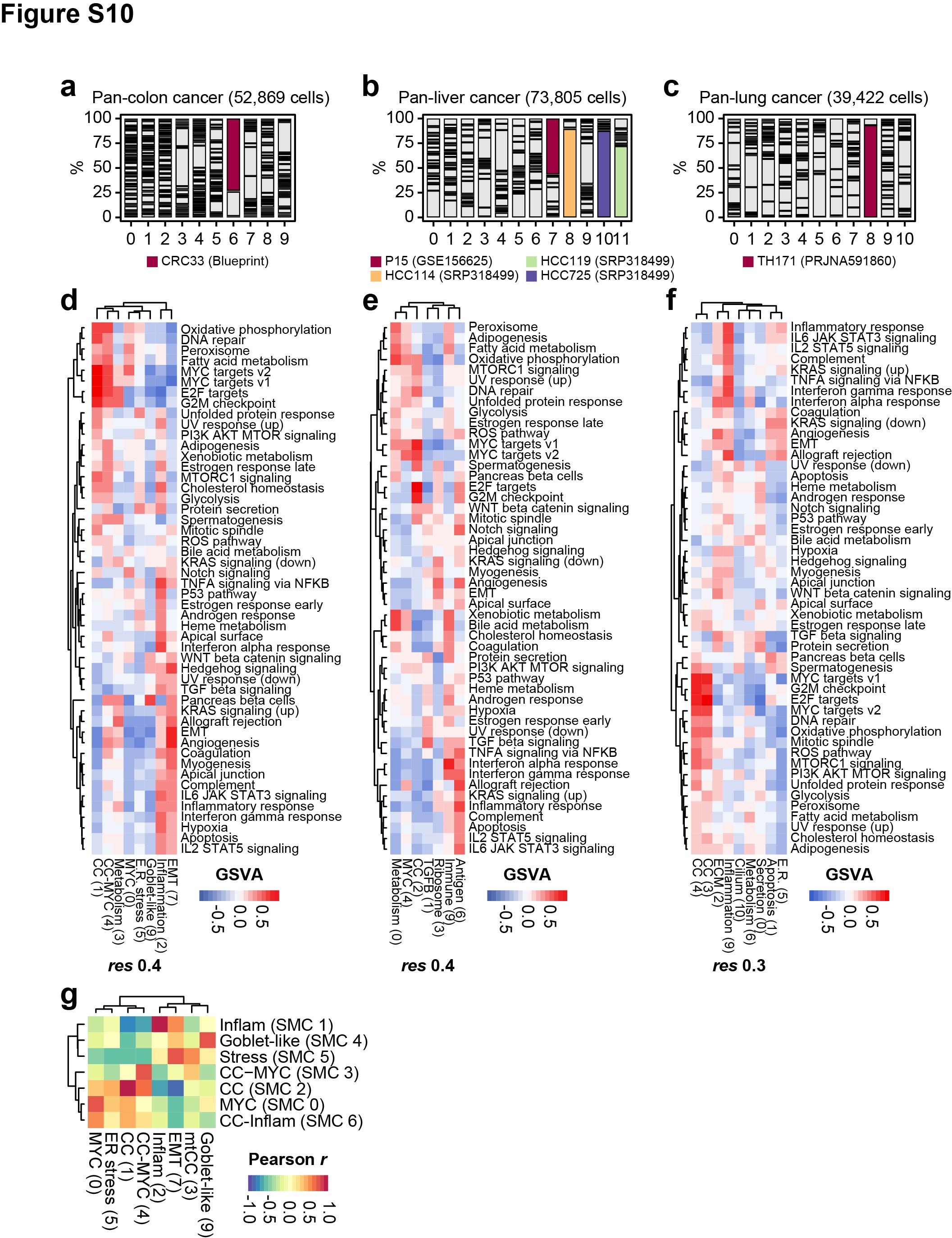

Supplement: Supplementary_Figure_10_bbad460 [file supplementary_figure_10_bbad460.jpeg]

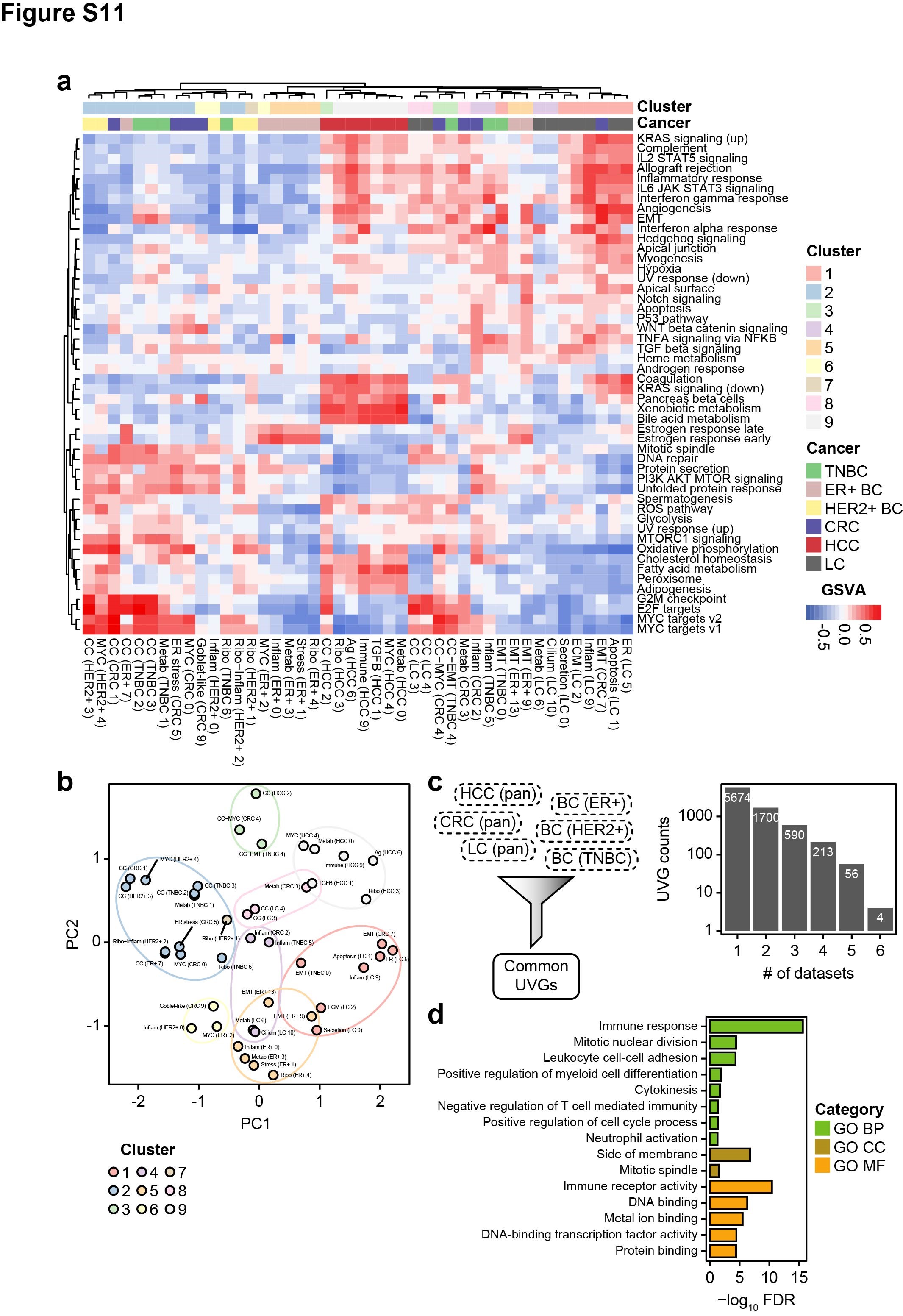

Supplement: Supplementary_Figure_11_bbad460 [file supplementary_figure_11_bbad460.jpeg]

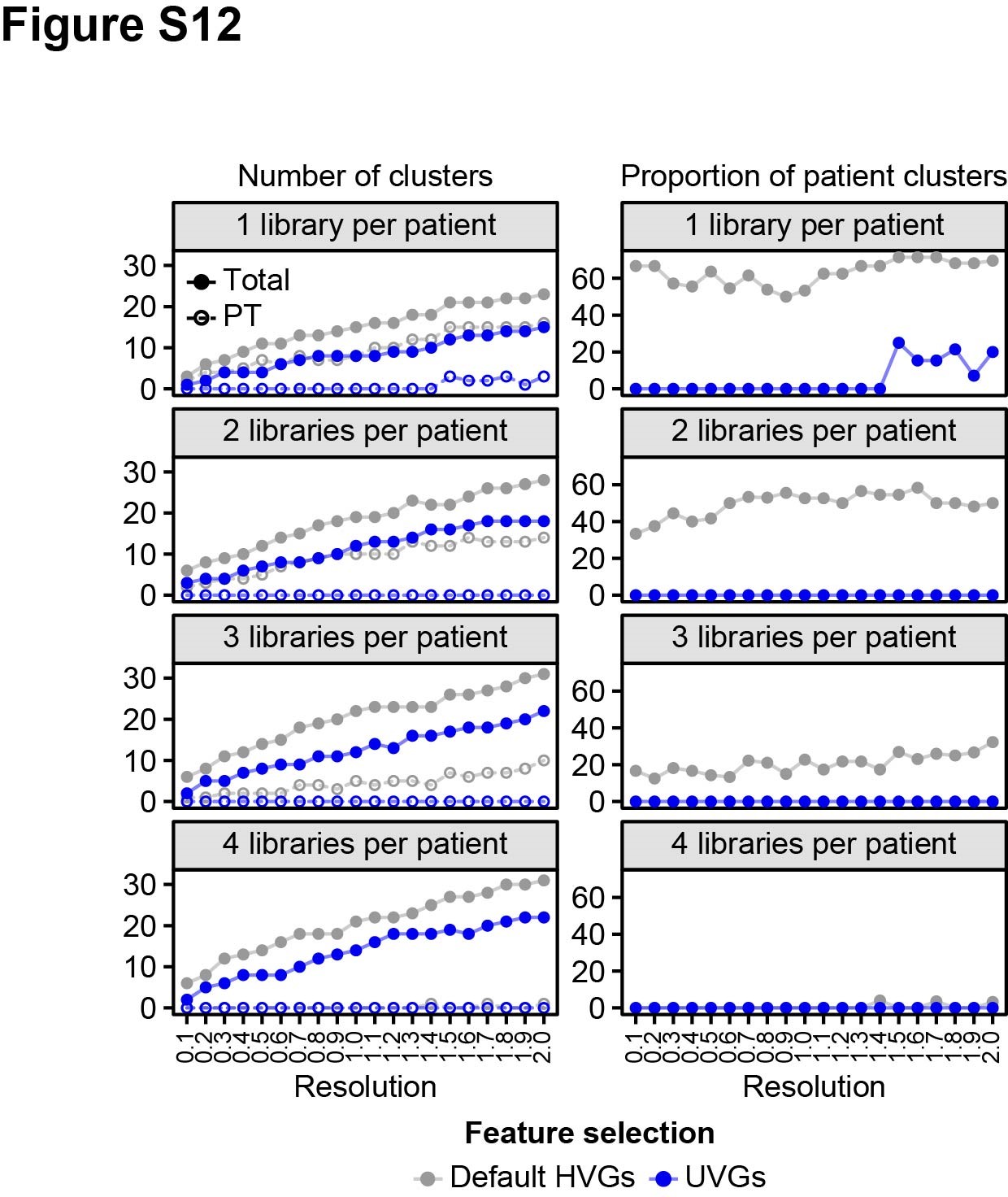

Supplement: Supplementary_Figure_12_bbad460 [file supplementary_figure_12_bbad460.jpeg]
