# Supplementary material for: Clustering malignant cell states using universally variable genes
Source: Brief Bioinform. 2023 Dec 11;25(1):bbad460. doi: 10.1093/bib/bbad460 (PMC10783859; doi:10.1093/bib/bbad460)
Supplement: Suppmentary_Table_1_bbad460 [file suppmentary_table_1_bbad460.pdf]

logFC	pct.1	pct.2	FDR	Cluster	Symbol	Confident marker
0.47	0.774	0.604	5.97E-159	0	*MYC*	X
0.27	0.834	0.665	4.83E-156	0	*EBPL*	X
0.27	0.685	0.495	1.71E-130	0	*ASCL2*	X
0.27	0.859	0.728	1.06E-129	0	*EIF3L*	X
0.6	0.841	0.721	1.02E-102	0	*CXCL3*	X
0.27	0.821	0.685	6.66E-97	0	*ZFP36L1*	X
0.27	0.764	0.624	9.43E-94	0	*FERMT1*	X
0.27	0.802	0.676	1.47E-88	0	*FCGRT*	X
0.55	0.442	0.302	2.14E-81	0	*MT1G*	X
0.41	0.661	0.524	6.05E-79	0	*EDN1*	X
0.4	0.422	0.304	3.70E-60	0	*FGGY*	X
0.26	0.528	0.399	1.57E-56	0	*ID3*	X
0.29	0.656	0.541	5.01E-47	0	*DDIT4*	X
0.29	0.523	0.414	2.16E-39	0	*MT1X*	X
0.35	0.728	0.635	2.32E-39	0	*CXCL8*	X
0.3	0.753	0.663	1.34E-36	0	*CXCL1*	X
0.27	0.489	0.405	8.56E-33	0	*SERPINH1*	X
0.39	0.559	0.484	5.44E-25	0	*ZFAND2A*	X
0.31	0.524	0.483	0.0075	0	*MT2A*	X
1.32	0.853	0.506	0	1	*TSPAN1*	X
1.2	0.619	0.359	0	1	*PLAC8*	X
1.19	0.853	0.663	0	1	*CEACAM6*	X
1.11	0.743	0.413	0	1	*CEACAM1*	X
1.08	0.778	0.484	0	1	*ERO1A*	X
1.06	0.79	0.437	0	1	*EMP1*	X
1.02	0.89	0.685	0	1	*MUC13*	X
1.02	0.761	0.314	0	1	*SDCBP2*	X
1.02	0.868	0.603	0	1	*CD55*	X
0.95	0.882	0.702	0	1	*FXYD3*	X
0.88	0.408	0.153	0	1	*KLK10*	O
0.84	0.794	0.522	0	1	*LAMB3*	X
0.82	0.821	0.628	0	1	*ASS1*	X
0.81	0.593	0.265	0	1	*ISG20*	X
0.77	0.786	0.584	0	1	*LMO7*	X
0.76	0.561	0.294	0	1	*SLC6A8*	X
0.75	0.813	0.535	0	1	*TMBIM1*	X
0.74	0.753	0.506	0	1	*SLC2A1*	X
0.71	0.685	0.399	0	1	*MALL*	X
0.69	0.888	0.701	0	1	*CAMK2N1*	X
0.69	0.713	0.485	0	1	*QSOX1*	X
0.67	0.795	0.607	0	1	*CD59*	X
0.64	0.837	0.655	0	1	*SERINC2*	X
0.63	0.531	0.182	0	1	*ABHD11-AS1*	O
0.62	0.446	0.152	0	1	*CDA*	O
0.62	0.409	0.121	0	1	*CLIC3*	O
0.61	0.85	0.669	0	1	*TSPAN3*	X
0.59	0.57	0.262	0	1	*RHOF*	X
0.57	0.696	0.395	0	1	*SLC16A3*	X

0.56	0.597	0.327	0	1	*OAS1*	X
0.56	0.444	0.168	0	1	*GCNT3*	O
0.54	0.769	0.552	0	1	*MYH14*	X
0.52	0.781	0.536	0	1	*SLC44A4*	X
0.68	0.901	0.741	2.57E-308	1	*FHL2*	X
0.6	0.75	0.554	3.12E-301	1	*PRSS8*	X
0.46	0.644	0.384	5.73E-301	1	*OPTN*	X
0.64	0.555	0.321	2.85E-295	1	*SLC40A1*	X
0.4	0.288	0.074	6.43E-291	1	*TIMP2*	O
0.53	0.658	0.461	3.01E-283	1	*TMPRSS2*	X
0.51	0.727	0.539	5.84E-274	1	*PTTG1IP*	X
0.42	0.377	0.145	9.23E-269	1	*COL17A1*	O
0.43	0.748	0.559	5.91E-265	1	*STK24*	X
0.53	0.892	0.746	4.10E-263	1	*CTSD*	X
0.45	0.703	0.506	3.45E-260	1	*BCL2L1*	X
0.47	0.73	0.519	2.21E-259	1	*ITGB4*	X
0.49	0.736	0.533	4.79E-258	1	*TMEM45B*	X
0.49	0.722	0.523	2.14E-254	1	*CXCL16*	X
0.52	0.84	0.687	6.46E-242	1	*DSG2*	X
0.49	0.774	0.591	4.70E-236	1	*CAPG*	X
0.46	0.808	0.666	3.67E-234	1	*SPINT1*	X
0.4	0.324	0.116	2.52E-228	1	*ACHE*	O
0.53	0.809	0.658	9.76E-228	1	*TINAGL1*	X
0.42	0.712	0.531	3.03E-222	1	*LRP10*	X
0.36	0.407	0.171	5.55E-221	1	*CDHR5*	O
0.42	0.7	0.508	7.26E-220	1	*FLNB*	X
0.45	0.82	0.676	9.05E-220	1	*ITGB1*	X
0.76	0.261	0.082	1.01E-219	1	*DUOX2*	O
0.32	0.505	0.277	1.81E-218	1	*RRAS*	X
0.37	0.708	0.532	2.09E-216	1	*CRB3*	X
0.38	0.425	0.229	2.27E-216	1	*HK2*	O
0.27	0.309	0.114	3.75E-216	1	*CLIC5*	O
0.38	0.39	0.192	1.22E-210	1	*LMTK3*	O
0.43	0.846	0.709	1.03E-209	1	*ST14*	X
0.27	0.294	0.099	2.64E-209	1	*LINC01559*	O
0.37	0.611	0.429	1.30E-208	1	*ADAM9*	X
0.36	0.605	0.407	2.37E-208	1	*PLS1*	X
0.43	0.363	0.154	2.21E-200	1	*MSLN*	O
0.38	0.725	0.553	9.17E-197	1	*SH3GLB1*	X
1.49	0.291	0.116	9.57E-196	1	*TFF2*	O
0.27	0.35	0.148	1.08E-194	1	*GBP3*	O
0.41	0.829	0.694	3.40E-193	1	*CDH1*	X
0.39	0.589	0.382	6.09E-190	1	*USP53*	X
0.41	0.775	0.624	7.67E-190	1	*TAX1BP3*	X
0.32	0.404	0.204	3.11E-187	1	*KRT80*	O
0.41	0.274	0.106	5.34E-186	1	*APOL1*	O
0.35	0.613	0.425	1.54E-185	1	*HEPH*	X
0.41	0.769	0.602	3.62E-184	1	*SPATS2L*	X
0.27	0.345	0.157	9.36E-184	1	*PPARD*	O

0.34	0.459	0.289	9.52E-183	1	*RHOD*	X
0.47	0.869	0.735	2.96E-182	1	*MISP*	X
0.3	0.46	0.253	4.54E-181	1	*SULT2B1*	X
0.33	0.492	0.297	2.65E-177	1	*P4HA1*	X
0.41	0.846	0.69	9.41E-177	1	*NBL1*	X
0.35	0.589	0.411	9.91E-176	1	*OCLN*	X
0.33	0.294	0.112	5.50E-175	1	*CDKN2B*	O
0.43	0.606	0.42	2.82E-174	1	*DSC2*	X
0.46	0.862	0.726	4.03E-174	1	*FXYD5*	X
0.38	0.627	0.444	2.04E-172	1	*PPIC*	X
0.37	0.645	0.481	3.05E-172	1	*SLC22A18*	X
0.31	0.582	0.398	2.22E-169	1	*SNX9*	X
0.41	0.792	0.647	8.99E-169	1	*CXADR*	X
0.53	0.625	0.433	6.21E-168	1	*TXNIP*	X
0.32	0.567	0.382	2.17E-167	1	*RIOK3*	X
0.33	0.404	0.219	2.50E-167	1	*PHLDA3*	O
0.34	0.601	0.413	8.31E-166	1	*INAVA*	X
0.43	0.831	0.699	1.76E-165	1	*GRN*	X
0.32	0.663	0.478	2.56E-163	1	*TMPRSS4*	X
0.74	0.669	0.55	3.57E-161	1	*SCD*	X
0.28	0.48	0.293	1.30E-160	1	*RNF103*	X
0.38	0.84	0.702	9.30E-160	1	*UBE2B*	X
0.43	0.475	0.296	2.26E-158	1	*DUSP5*	X
0.34	0.644	0.462	4.24E-158	1	*TNFRSF21*	X
0.34	0.838	0.704	2.81E-157	1	*CAST*	X
0.26	0.438	0.265	7.82E-156	1	*PCDH1*	X
0.32	0.46	0.297	1.93E-155	1	*ITGA2*	X
0.38	0.538	0.374	2.72E-155	1	*PPP1R14D*	X
0.37	0.737	0.581	3.59E-155	1	*CAPN2*	X
0.78	0.815	0.656	7.35E-155	1	*C15orf48*	X
0.35	0.805	0.655	2.07E-154	1	*MMP24OS*	X
0.31	0.341	0.183	8.60E-154	1	*PLOD2*	O
0.41	0.297	0.161	2.01E-153	1	*AL121761.1*	O
0.67	0.469	0.292	9.59E-153	1	*KRT7*	X
0.69	0.593	0.389	7.42E-152	1	*ANXA1*	X
0.38	0.552	0.388	9.65E-152	1	*KCNK1*	X
0.26	0.438	0.263	2.20E-151	1	*FA2H*	X
0.71	0.692	0.557	5.67E-150	1	*INSIG1*	X
0.97	0.77	0.617	2.38E-147	1	*CKB*	X
0.29	0.328	0.153	1.31E-146	1	*HPGD*	O
0.37	0.762	0.579	5.27E-146	1	*ANXA3*	X
0.28	0.291	0.124	7.99E-145	1	*C4BPB*	O
0.34	0.746	0.6	1.34E-144	1	*EFHD2*	X
0.28	0.631	0.458	1.41E-144	1	*SCARB2*	X
0.34	0.621	0.469	1.66E-144	1	*B4GALT5*	X
0.31	0.59	0.426	3.17E-144	1	*EFNB1*	X
0.37	0.518	0.331	3.68E-144	1	*B4GALT1*	X
0.31	0.522	0.342	9.86E-144	1	*BNIP3L*	X
0.3	0.666	0.49	2.04E-143	1	*BAIAP2L1*	X

0.3	0.656	0.502	4.36E-141	1	*TNIP1*	X
0.4	0.657	0.498	2.59E-140	1	*BHLHE40*	X
0.26	0.493	0.325	7.46E-140	1	*CDCP1*	X
0.36	0.801	0.685	9.79E-139	1	*AP3S1*	X
0.32	0.589	0.443	2.21E-137	1	*PLIN3*	X
0.3	0.694	0.547	2.85E-137	1	*DDR1*	X
0.37	0.53	0.347	4.25E-135	1	*LAMA3*	X
0.33	0.703	0.554	5.30E-135	1	*CHP1*	X
0.3	0.494	0.342	9.95E-135	1	*SORL1*	X
0.29	0.41	0.239	1.41E-134	1	*AC008397.1*	O
0.33	0.736	0.59	2.00E-134	1	*PLSCR1*	X
0.35	0.824	0.701	2.47E-134	1	*CAP1*	X
0.36	0.469	0.316	6.71E-134	1	*UPP1*	X
0.26	0.483	0.313	8.03E-134	1	*FNDC3B*	X
0.31	0.702	0.549	4.48E-131	1	*IFNGR2*	X
0.37	0.71	0.561	1.39E-130	1	*CTSS*	X
0.25	0.416	0.241	7.62E-130	1	*P4HA2*	O
0.45	0.329	0.167	1.06E-129	1	*UCA1*	O
0.27	0.458	0.278	6.60E-129	1	*BCL2L15*	X
0.32	0.866	0.735	9.51E-129	1	*TAX1BP1*	X
0.27	0.54	0.398	7.62E-128	1	*NIBAN2*	X
0.41	0.761	0.616	1.38E-127	1	*EPHA2*	X
0.26	0.389	0.236	2.07E-127	1	*GALNT5*	O
0.25	0.551	0.381	1.26E-126	1	*GNG12*	X
1.34	0.572	0.511	6.64E-126	1	*FABP1*	X
0.35	0.626	0.494	1.85E-125	1	*ABHD2*	X
0.26	0.452	0.299	2.40E-123	1	*UGCG*	X
0.28	0.616	0.466	1.09E-121	1	*PDLIM5*	X
0.33	0.736	0.59	1.60E-121	1	*PDXK*	X
0.31	0.836	0.727	3.32E-119	1	*SELENOS*	X
0.3	0.71	0.581	8.76E-119	1	*CTSZ*	X
0.25	0.329	0.171	2.34E-117	1	*H2BC5*	O
0.26	0.576	0.434	3.03E-117	1	*NPEPPS*	X
0.31	0.81	0.689	6.94E-117	1	*ARF6*	X
0.42	0.765	0.638	1.54E-116	1	*CDC42EP5*	X
0.36	0.761	0.639	2.04E-116	1	*MIDN*	X
0.3	0.761	0.637	1.10E-115	1	*TAPBP*	X
0.28	0.758	0.639	1.33E-115	1	*CTNNA1*	X
0.3	0.522	0.35	5.35E-115	1	*GJB3*	X
0.27	0.587	0.436	2.71E-114	1	*F2RL1*	X
0.26	0.584	0.414	1.91E-113	1	*FOSL2*	X
0.31	0.792	0.678	5.29E-113	1	*PPP1CB*	X
0.27	0.618	0.473	1.27E-112	1	*ITPRID2*	X
0.29	0.298	0.143	1.70E-112	1	*RNF186*	O
0.31	0.725	0.595	2.67E-112	1	*KLF3*	X
0.31	0.815	0.699	3.86E-112	1	*APLP2*	X
0.32	0.642	0.49	3.33E-111	1	*EFNB2*	X
0.29	0.812	0.686	6.21E-110	1	*SPTBN1*	X
0.32	0.72	0.605	4.95E-108	1	*NECTIN2*	X

0.3	0.368	0.269	9.54E-108	1	*TRIM29*	X
0.27	0.779	0.656	1.31E-107	1	*UFM1*	X
0.26	0.599	0.454	2.13E-107	1	*MYO5B*	X
0.28	0.82	0.706	3.25E-107	1	*VAPA*	X
0.3	0.508	0.368	3.43E-106	1	*PIM1*	X
0.32	0.717	0.6	9.24E-106	1	*NT5C3A*	X
0.26	0.519	0.35	2.91E-105	1	*IL18*	X
0.74	0.445	0.31	1.42E-104	1	*LGALS1*	X
0.3	0.72	0.599	1.01E-102	1	*C6orf132*	X
0.26	0.63	0.487	1.52E-102	1	*PCSK7*	X
0.43	0.678	0.575	4.53E-101	1	*GLRX*	X
0.25	0.554	0.417	4.85E-101	1	*FTH1P10*	X
0.28	0.826	0.717	3.88E-100	1	*LRRFIP1*	X
0.35	0.807	0.693	8.83E-99	1	*TOB1*	X
0.28	0.62	0.509	2.60E-98	1	*CFLAR*	X
0.42	0.674	0.507	2.75E-98	1	*PLAAT3*	X
0.3	0.571	0.455	1.85E-97	1	*GALNT1*	X
1.05	0.674	0.563	1.26E-96	1	*S100A4*	X
0.3	0.837	0.731	4.31E-96	1	*MAP1LC3B*	X
0.39	0.72	0.594	4.01E-95	1	*PMEPA1*	X
0.27	0.379	0.238	5.63E-95	1	*SERPINE2*	O
0.26	0.606	0.469	9.27E-95	1	*UBE2H*	X
0.43	0.554	0.423	9.41E-95	1	*CES2*	X
0.27	0.795	0.673	1.78E-94	1	*DSP*	X
0.28	0.798	0.678	1.68E-92	1	*MYH9*	X
0.29	0.735	0.627	5.39E-92	1	*HIGD1A*	X
0.27	0.55	0.453	4.82E-90	1	*TXNRD1*	X
0.26	0.595	0.453	1.19E-88	1	*IFT172*	X
0.26	0.711	0.597	5.66E-87	1	*NDFIP1*	X
0.45	0.784	0.678	1.28E-84	1	*SDC4*	X
0.26	0.723	0.624	6.81E-84	1	*TRAM1*	X
0.26	0.817	0.713	1.47E-82	1	*ARPC1B*	X
0.25	0.826	0.706	6.25E-81	1	*ZFAND5*	X
0.28	0.665	0.543	1.62E-80	1	*WSB1*	X
0.31	0.774	0.684	4.52E-80	1	*CD47*	X
0.48	0.359	0.242	5.72E-78	1	*S100A2*	O
0.36	0.611	0.469	5.45E-77	1	*H1-0*	X
0.3	0.386	0.255	9.73E-77	1	*KLF2*	X
0.26	0.535	0.414	2.29E-74	1	*MACC1*	X
0.26	0.772	0.671	1.26E-73	1	*CTSB*	X
0.26	0.556	0.451	7.92E-72	1	*INO80C*	X
0.27	0.819	0.719	2.80E-68	1	*SDCBP*	X
0.28	0.806	0.7	2.70E-67	1	*CARHSP1*	X
0.42	0.418	0.299	6.83E-66	1	*BIRC3*	X
0.35	0.427	0.299	1.78E-65	1	*FGFBP1*	X
0.3	0.695	0.583	2.61E-65	1	*TMEM176A*	X
0.44	0.327	0.257	1.04E-63	1	*RAMP1*	X
0.29	0.462	0.353	4.81E-63	1	*SERPINB5*	X
0.31	0.531	0.435	8.93E-62	1	*MSMO1*	X

0.34	0.501	0.412	2.76E-60	1	*TRIB3*	X
0.28	0.557	0.434	5.50E-60	1	*HES4*	X
0.25	0.694	0.604	2.63E-57	1	*LRATD1*	X
1.14	0.299	0.209	4.85E-56	1	*KRT17*	O
0.26	0.306	0.203	5.30E-55	1	*PLAAT4*	O
0.29	0.567	0.446	8.68E-55	1	*PRSS23*	X
0.33	0.576	0.472	6.87E-53	1	*HMGCS1*	X
0.25	0.62	0.527	3.78E-48	1	*EFNA1*	X
0.26	0.342	0.251	8.81E-47	1	*RCAN1*	X
0.29	0.73	0.68	1.88E-31	1	*CCND1*	X
0.3	0.3	0.263	1.94E-24	1	*RGCC*	X
0.25	0.591	0.522	3.54E-20	1	*SOD3*	X
1.98	0.765	0.142	0	2	*UBE2C*	O
1.86	0.933	0.448	0	2	*HMGB2*	X
1.65	0.846	0.26	0	2	*PTTG1*	X
1.25	0.809	0.232	0	2	*PCLAF*	O
1.23	0.893	0.533	0	2	*CKS1B*	X
1.17	0.82	0.12	0	2	*BIRC5*	O
1.16	0.869	0.259	0	2	*CENPW*	X
1.15	0.78	0.185	0	2	*CDKN3*	O
1.14	0.93	0.684	0	2	*TUBB*	X
1.04	0.616	0.099	0	2	*CCNB1*	O
1.02	0.827	0.529	0	2	*UBE2S*	X
0.99	0.678	0.098	0	2	*CDC20*	O
0.99	0.691	0.09	0	2	*RRM2*	O
0.97	0.773	0.18	0	2	*MAD2L1*	O
0.96	0.928	0.715	0	2	*CKS2*	X
0.94	0.762	0.398	0	2	*PCNA*	X
0.93	0.922	0.71	0	2	*NUCKS1*	X
0.92	0.755	0.168	0	2	*TK1*	O
0.92	0.733	0.105	0	2	*MKI67*	O
0.88	0.635	0.093	0	2	*CENPF*	O
0.87	0.829	0.365	0	2	*DTYMK*	X
0.86	0.601	0.047	0	2	*TOP2A*	O
0.83	0.665	0.072	0	2	*TPX2*	O
0.81	0.678	0.177	0	2	*UBE2T*	O
0.79	0.517	0.044	0	2	*CDK1*	O
0.77	0.737	0.244	0	2	*SMC4*	O
0.73	0.872	0.568	0	2	*RPA3*	X
0.72	0.577	0.118	0	2	*CCNB2*	O
0.71	0.814	0.469	0	2	*HMGB3*	X
0.69	0.653	0.125	0	2	*ZWINT*	O
0.68	0.827	0.548	0	2	*RAD21*	X
0.68	0.909	0.702	0	2	*GGCT*	X
0.67	0.818	0.57	0	2	*DUT*	X
0.66	0.676	0.329	0	2	*KPNA2*	X
0.61	0.902	0.712	0	2	*PA2G4*	X
0.61	0.618	0.158	0	2	*TYMS*	O
0.6	0.885	0.703	0	2	*SIVA1*	X

0.6	0.729	0.251	0	2	*CENPN*	X
0.58	0.47	0.037	0	2	*PLK1*	O
0.58	0.78	0.387	0	2	*CCDC34*	X
0.58	0.57	0.071	0	2	*MYBL2*	O
0.57	0.607	0.148	0	2	*CENPM*	O
0.56	0.758	0.441	0	2	*WDR34*	X
0.55	0.501	0.029	0	2	*CCNA2*	O
0.52	0.723	0.366	0	2	*MZT1*	X
0.52	0.583	0.155	0	2	*GINS2*	O
0.52	0.634	0.171	0	2	*SMC2*	O
0.51	0.667	0.211	0	2	*SKA2*	O
0.5	0.728	0.367	0	2	*SNRNP25*	X
0.5	0.457	0.03	0	2	*AURKB*	O
0.5	0.452	0.112	0	2	*AURKA*	O
0.49	0.43	0.027	0	2	*CENPA*	O
0.48	0.49	0.035	0	2	*PBK*	O
0.47	0.699	0.326	0	2	*TMPO*	X
0.47	0.522	0.077	0	2	*TROAP*	O
0.46	0.687	0.347	0	2	*CSE1L*	X
0.46	0.646	0.271	0	2	*ANP32E*	X
0.46	0.4	0.026	0	2	*HMMR*	O
0.45	0.585	0.173	0	2	*DHFR*	O
0.45	0.628	0.257	0	2	*GMNN*	X
0.45	0.631	0.252	0	2	*DNMT1*	X
0.44	0.456	0.046	0	2	*CDCA3*	O
0.44	0.501	0.085	0	2	*KIF20B*	O
0.43	0.504	0.076	0	2	*MND1*	O
0.42	0.503	0.085	0	2	*CENPU*	O
0.42	0.533	0.14	0	2	*CDT1*	O
0.42	0.571	0.189	0	2	*ECT2*	O
0.41	0.417	0.096	0	2	*ATAD2*	O
0.41	0.541	0.199	0	2	*SAPCD2*	O
0.4	0.452	0.06	0	2	*ANLN*	O
0.4	0.585	0.185	0	2	*RRM1*	O
0.4	0.42	0.023	0	2	*NUF2*	O
0.4	0.449	0.099	0	2	*CKAP2*	O
0.4	0.661	0.312	0	2	*DNAJC9*	X
0.39	0.406	0.018	0	2	*SPC25*	O
0.39	0.581	0.208	0	2	*PHF19*	O
0.38	0.623	0.257	0	2	*LMNB2*	X
0.38	0.562	0.18	0	2	*CENPH*	O
0.38	0.591	0.252	0	2	*PSIP1*	X
0.37	0.438	0.065	0	2	*PRR11*	O
0.37	0.444	0.039	0	2	*SGO1*	O
0.37	0.464	0.11	0	2	*FEN1*	O
0.36	0.371	0.024	0	2	*NEK2*	O
0.36	0.474	0.053	0	2	*FOXM1*	O
0.36	0.432	0.033	0	2	*CDCA5*	O
0.35	0.416	0.03	0	2	*CEP55*	O

0.35	0.409	0.047	0	2	*RAD51AP1*	O
0.34	0.5	0.158	0	2	*KIF22*	O
0.34	0.42	0.054	0	2	*ASF1B*	O
0.33	0.449	0.076	0	2	*CENPK*	O
0.32	0.463	0.107	0	2	*LMNB1*	O
0.32	0.407	0.055	0	2	*RACGAP1*	O
0.31	0.427	0.076	0	2	*ORC6*	O
0.31	0.382	0.04	0	2	*PRC1*	O
0.31	0.376	0.045	0	2	*TACC3*	O
0.31	0.496	0.158	0	2	*MIS18A*	O
0.3	0.419	0.112	0	2	*HELLS*	O
0.29	0.327	0.028	0	2	*CENPE*	O
0.29	0.333	0.02	0	2	*GTSE1*	O
0.28	0.391	0.089	0	2	*RFC3*	O
0.28	0.383	0.047	0	2	*SKA3*	O
0.28	0.355	0.035	0	2	*MELK*	O
0.27	0.388	0.06	0	2	*POC1A*	O
0.27	0.331	0.067	0	2	*KNSTRN*	O
0.27	0.35	0.033	0	2	*PKMYT1*	O
0.27	0.324	0.05	0	2	*CLSPN*	O
0.26	0.293	0.015	0	2	*ASPM*	O
0.26	0.381	0.07	0	2	*TRIP13*	O
0.26	0.39	0.062	0	2	*FANCI*	O
0.26	0.318	0.02	0	2	*KIF4A*	O
0.26	0.344	0.043	0	2	*CDCA8*	O
0.25	0.409	0.11	0	2	*CHEK1*	O
0.63	0.909	0.729	1.32e-315	2	*NME1*	X
0.3	0.485	0.164	2.75e-314	2	*MCM4*	O
0.27	0.399	0.11	8.32e-312	2	*VRK1*	O
0.38	0.682	0.355	5.77E-305	2	*BCL2L12*	X
0.53	0.862	0.659	2.71E-303	2	*TMEM106C*	X
0.45	0.717	0.398	7.49E-300	2	*NASP*	X
0.73	0.928	0.745	3.26E-299	2	*LDHB*	X
0.28	0.336	0.079	7.87E-292	2	*FAM83D*	O
0.46	0.708	0.42	2.08E-285	2	*LBR*	X
0.34	0.538	0.224	4.47E-284	2	*MCM3*	O
0.35	0.575	0.264	2.35E-269	2	*CTNNAL1*	X
0.43	0.82	0.569	9.41E-269	2	*SSRP1*	X
0.41	0.71	0.424	3.37E-265	2	*TFDP1*	X
0.49	0.859	0.655	7.03E-265	2	*ILF2*	X
0.7	0.891	0.7	7.47E-264	2	*FABP5*	X
0.47	0.827	0.594	1.31E-260	2	*TOMM40*	X
0.62	0.867	0.716	2.54E-260	2	*ARL6IP1*	X
0.46	0.778	0.516	3.80E-260	2	*RBBP7*	X
0.3	0.541	0.236	1.21E-258	2	*ITGB3BP*	O
0.39	0.689	0.395	2.16E-258	2	*PPIH*	X
0.41	0.662	0.374	6.11E-255	2	*EXOSC8*	X
0.41	0.773	0.497	1.59E-254	2	*HADH*	X
0.5	0.822	0.567	1.60E-253	2	*PAICS*	X

0.45	0.823	0.603	6.66E-248	2	*POLD2*	X
0.43	0.874	0.677	2.68E-247	2	*LSM2*	X
0.29	0.501	0.215	6.86E-246	2	*TUBG1*	O
0.37	0.661	0.371	2.83E-245	2	*POLR3K*	X
0.43	0.835	0.639	7.63E-243	2	*HNRNPD*	X
0.27	0.449	0.172	8.07E-242	2	*RFC2*	O
0.37	0.679	0.396	3.54E-238	2	*COMMD4*	X
0.29	0.563	0.267	2.71E-235	2	*SAC3D1*	X
0.43	0.853	0.657	4.84E-234	2	*PPM1G*	X
0.4	0.676	0.4	7.16E-229	2	*LYAR*	X
0.33	0.569	0.288	9.57E-226	2	*NUP37*	X
0.4	0.733	0.462	2.99E-225	2	*HPRT1*	X
0.51	0.877	0.684	5.55E-224	2	*CACYBP*	X
0.41	0.781	0.537	1.45E-223	2	*CKLF*	X
0.49	0.857	0.67	4.77E-221	2	*CCT5*	X
0.39	0.851	0.652	2.87E-219	2	*MRPL11*	X
0.32	0.613	0.331	5.84E-219	2	*SMC1A*	X
0.32	0.591	0.313	1.32E-218	2	*USP1*	X
0.5	0.869	0.675	2.26E-216	2	*EBP*	X
0.39	0.822	0.594	4.95E-212	2	*HNRNPR*	X
0.33	0.642	0.362	1.22E-209	2	*CMSS1*	X
0.31	0.667	0.382	8.22E-209	2	*PRPS2*	X
0.39	0.787	0.55	2.27E-207	2	*TPRKB*	X
0.33	0.652	0.373	2.31E-206	2	*TMEM97*	X
0.28	0.636	0.353	3.81E-203	2	*SAE1*	X
0.3	0.559	0.284	4.83E-203	2	*HAUS1*	X
0.29	0.612	0.335	7.52E-201	2	*BRI3BP*	X
0.43	0.789	0.573	9.38E-198	2	*CDK4*	X
0.38	0.792	0.563	3.79E-196	2	*POP7*	X
0.33	0.659	0.394	1.31E-194	2	*AC106795.1*	X
0.28	0.599	0.33	5.05E-194	2	*ALYREF*	X
0.33	0.689	0.427	2.23E-192	2	*PARP1*	X
0.33	0.742	0.498	2.15E-188	2	*MAZ*	X
0.41	0.857	0.684	2.92E-185	2	*MTCH2*	X
0.34	0.746	0.506	4.45E-183	2	*BCL7C*	X
0.43	0.85	0.656	7.02E-183	2	*GLO1*	X
0.4	0.851	0.616	7.24E-183	2	*GGH*	X
0.37	0.866	0.674	2.89E-182	2	*LSM8*	X
0.28	0.611	0.348	1.07E-181	2	*MAGOHB*	X
0.41	0.771	0.548	1.15E-181	2	*SNRPA1*	X
0.37	0.77	0.546	2.09E-181	2	*LSM6*	X
0.5	0.889	0.747	9.25E-181	2	*PSMA4*	X
0.33	0.666	0.421	2.54E-180	2	*RNASEH2B*	X
0.28	0.671	0.417	1.45E-179	2	*COPRS*	X
0.42	0.869	0.7	3.13E-179	2	*CCT2*	X
0.4	0.863	0.686	2.10E-175	2	*CCT7*	X
0.34	0.748	0.511	6.06E-175	2	*EEF1E1*	X
0.37	0.872	0.693	9.37E-174	2	*SNRPC*	X
0.39	0.783	0.554	1.14E-173	2	*C20orf27*	X

0.4	0.846	0.65	2.49E-173	2	*GSTO1*	X
0.33	0.778	0.572	4.62E-173	2	*MRPL37*	X
0.35	0.824	0.604	2.81E-172	2	*BOLA3*	X
0.35	0.751	0.522	2.37E-171	2	*NUDT5*	X
0.38	0.884	0.731	1.93E-170	2	*SLC25A39*	X
0.34	0.809	0.603	2.77E-169	2	*KPNB1*	X
0.32	0.63	0.369	9.11E-168	2	*ACAT2*	X
0.39	0.843	0.66	4.63E-165	2	*XRCC6*	X
0.28	0.641	0.387	5.92E-165	2	*PRKDC*	X
0.38	0.846	0.666	1.73E-164	2	*DCTPP1*	X
0.4	0.884	0.716	2.51E-164	2	*AHCY*	X
0.32	0.719	0.492	1.56E-162	2	*RUVBL2*	X
0.28	0.752	0.519	1.66E-161	2	*NUDT21*	X
0.34	0.785	0.592	4.76E-161	2	*ZDHHC12*	X
0.28	0.711	0.468	8.21E-161	2	*ANAPC15*	X
0.35	0.83	0.634	1.59E-159	2	*SRPK1*	X
0.34	0.754	0.526	4.30E-158	2	*GCSH*	X
0.39	0.864	0.696	2.05E-157	2	*TCP1*	X
0.25	0.605	0.356	2.22E-157	2	*PXMP2*	X
0.29	0.612	0.373	2.55E-157	2	*FH*	X
0.3	0.804	0.589	1.38E-155	2	*SNX5*	X
0.32	0.832	0.626	5.18E-154	2	*UQCC3*	X
0.37	0.792	0.6	1.41E-152	2	*VDAC3*	X
0.35	0.85	0.688	1.55E-152	2	*HDGF*	X
0.28	0.624	0.385	4.72E-152	2	*SMC3*	X
0.34	0.726	0.506	1.12E-151	2	*HACD3*	X
0.32	0.725	0.504	6.11E-150	2	*VBP1*	X
0.3	0.729	0.505	8.30E-148	2	*TIMM10*	X
0.3	0.749	0.53	5.86E-147	2	*PSMC3*	X
0.29	0.614	0.384	1.09E-146	2	*ACTL6A*	X
0.29	0.658	0.437	1.51E-146	2	*SIGMAR1*	X
0.28	0.787	0.574	2.74E-146	2	*DAZAP1*	X
0.31	0.735	0.524	1.58E-145	2	*GLRX5*	X
0.26	0.67	0.435	2.10E-145	2	*CMC2*	X
0.35	0.753	0.55	1.64E-144	2	*PSMD14*	X
0.34	0.853	0.676	4.49E-144	2	*HNRNPM*	X
0.31	0.831	0.651	2.28E-143	2	*PAFAH1B3*	X
0.32	0.713	0.484	2.69E-143	2	*PSMG1*	X
0.34	0.862	0.708	4.47E-143	2	*TECR*	X
0.31	0.746	0.529	1.16E-141	2	*TRAP1*	X
0.27	0.61	0.377	1.31E-140	2	*TPGS2*	X
0.3	0.762	0.56	2.56E-140	2	*CHCHD3*	X
0.3	0.279	0.1	5.76E-140	2	*NMU*	O
0.29	0.662	0.443	1.30E-139	2	*MRPL16*	X
0.32	0.814	0.626	2.06E-139	2	*SYNCRIP*	X
0.31	0.782	0.57	1.11E-138	2	*NPM3*	X
0.27	0.683	0.456	3.34E-138	2	*MMAB*	X
0.35	0.804	0.628	6.27E-137	2	*PRMT1*	X
0.32	0.742	0.531	6.62E-136	2	*DKC1*	X

0.33	0.84	0.66	1.05E-135	2	*PGAM1*	X
0.33	0.827	0.646	3.37E-135	2	*MRPL4*	X
0.29	0.752	0.54	7.48E-135	2	*NUDCD2*	X
0.29	0.673	0.454	4.89E-134	2	*SLBP*	X
0.32	0.824	0.653	2.44E-133	2	*NDUFV1*	X
0.36	0.816	0.644	2.95E-132	2	*MRPL13*	X
0.37	0.757	0.573	4.29E-132	2	*PDHA1*	X
0.38	0.812	0.646	1.47E-131	2	*PSMA3*	X
0.33	0.827	0.667	4.53E-131	2	*CNIH4*	X
0.27	0.81	0.614	4.74E-131	2	*RBM17*	X
0.34	0.856	0.704	4.13E-130	2	*VPS29*	X
0.27	0.655	0.427	1.87E-129	2	*GCHFR*	X
0.26	0.728	0.521	1.34E-128	2	*ITGB1BP1*	X
0.35	0.889	0.727	1.44E-128	2	*IMPDH2*	X
0.3	0.834	0.665	1.31E-127	2	*POLR2E*	X
0.34	0.857	0.714	5.64E-127	2	*PSMB2*	X
0.29	0.747	0.547	8.38E-127	2	*PCMT1*	X
0.3	0.839	0.663	2.66E-125	2	*HDAC2*	X
0.29	0.86	0.701	2.97E-125	2	*MRPS15*	X
0.27	0.539	0.317	4.82E-125	2	*CDC25B*	X
0.3	0.777	0.581	1.75E-123	2	*EMP2*	X
0.31	0.814	0.634	2.44E-123	2	*CD320*	X
0.33	0.851	0.696	3.55E-123	2	*UBE2N*	X
0.25	0.726	0.515	1.12E-122	2	*UBA2*	X
0.32	0.871	0.724	4.45E-122	2	*CCT4*	X
0.26	0.741	0.534	3.31E-121	2	*CENPV*	X
0.27	0.854	0.676	4.74E-121	2	*MRPL18*	X
0.27	0.651	0.432	1.34E-120	2	*TUBA4A*	X
0.29	0.857	0.708	1.86E-120	2	*PDAP1*	X
0.35	0.808	0.643	8.89E-118	2	*MRPS26*	X
0.33	0.742	0.553	1.66E-117	2	*MDH1*	X
0.26	0.705	0.504	6.77E-117	2	*MALSU1*	X
0.3	0.867	0.725	3.01E-116	2	*PHB2*	X
0.27	0.477	0.276	1.32E-115	2	*CRNDE*	X
0.29	0.874	0.729	1.66E-115	2	*YWHAH*	X
0.26	0.851	0.681	1.87E-115	2	*CALM3*	X
0.28	0.843	0.675	1.27E-114	2	*NDUFAF8*	X
0.27	0.716	0.519	1.41E-113	2	*MRPL22*	X
0.28	0.867	0.709	2.03E-113	2	*RBMX*	X
0.34	0.851	0.699	3.16E-113	2	*SSB*	X
0.34	0.859	0.737	3.27E-113	2	*MRPL12*	X
0.29	0.798	0.627	1.07E-112	2	*POLR2H*	X
0.27	0.711	0.509	1.54E-112	2	*NOP58*	X
0.32	0.827	0.665	3.12E-111	2	*SUCLG1*	X
0.28	0.852	0.699	9.18E-111	2	*ARPC5L*	X
0.26	0.657	0.458	3.17E-110	2	*NAE1*	X
0.3	0.86	0.696	1.07E-109	2	*FDPS*	X
0.3	0.823	0.656	2.46E-109	2	*SEPTIN7*	X
0.26	0.703	0.5	1.36E-107	2	*MRPL17*	X

0.28	0.787	0.611	2.23E-107	2	*EBNA1BP2*	X
0.31	0.837	0.686	7.95E-107	2	*PSMA5*	X
0.26	0.847	0.682	1.42E-106	2	*BAX*	X
0.31	0.819	0.659	8.42E-106	2	*PSMD7*	X
0.25	0.717	0.523	9.97E-106	2	*MRPS25*	X
0.28	0.849	0.702	1.61E-105	2	*PLEKHJ1*	X
0.26	0.789	0.613	9.80E-105	2	*PSMC5*	X
0.25	0.797	0.621	1.76E-104	2	*RCN2*	X
0.28	0.877	0.741	1.79E-104	2	*ENSA*	X
0.27	0.867	0.708	1.11E-102	2	*MRPS12*	X
0.28	0.841	0.688	4.51E-102	2	*HSD17B10*	X
0.27	0.776	0.6	5.60E-102	2	*C7orf50*	X
0.27	0.666	0.475	1.07E-101	2	*CDC123*	X
0.28	0.871	0.726	2.56E-101	2	*PPP1CC*	X
0.3	0.86	0.729	5.78E-100	2	*ECHS1*	X
0.26	0.878	0.742	2.11E-99	2	*RAD23A*	X
0.29	0.832	0.671	9.43E-99	2	*CCT8*	X
0.26	0.84	0.674	4.47E-98	2	*NAA20*	X
0.28	0.728	0.55	7.16E-97	2	*SRM*	X
0.27	0.859	0.723	4.32E-96	2	*NOL7*	X
0.3	0.837	0.683	1.36E-94	2	*EIF4EBP1*	X
0.25	0.745	0.567	1.56E-94	2	*RABL6*	X
0.46	0.869	0.742	4.42E-94	2	*STRAP*	X
0.28	0.866	0.731	6.49E-92	2	*PSMA2*	X
0.27	0.864	0.733	3.91E-89	2	*PSMB6*	X
0.26	0.825	0.676	2.73E-86	2	*ADI1*	X
0.26	0.834	0.691	3.04E-86	2	*THOC7*	X
0.25	0.813	0.66	3.40E-85	2	*NDUFB6*	X
0.26	0.747	0.583	6.02E-84	2	*PPA2*	X
0.27	0.778	0.608	2.78E-83	2	*FDFT1*	X
0.26	0.82	0.677	8.38E-81	2	*NUDC*	X
0.25	0.865	0.732	2.94E-79	2	*GTF3A*	X
0.26	0.863	0.742	5.28E-79	2	*NAA10*	X
0.29	0.766	0.622	3.64E-75	2	*DPM1*	X
0.28	0.862	0.745	5.87E-72	2	*RPN2*	X
0.26	0.848	0.733	3.15E-58	2	*SNRPB2*	X
0.3	0.65	0.509	1.16E-57	2	*PSMB9*	X
0.3	0.752	0.631	6.95E-54	2	*SNHG3*	X
0.28	0.276	0.173	1.44E-36	2	*HULC*	O
0.42	0.256	0.601	0	3	*RAB13*	X
0.37	0.358	0.678	0	3	*BLVRB*	X
0.35	0.379	0.722	0	3	*ATOX1*	X
0.33	0.294	0.6	0	3	*PIN4*	X
0.29	0.29	0.607	0	3	*AC073861.1*	X
0.26	0.367	0.713	0	3	*ESD*	X
0.27	0.237	0.464	2.34E-274	3	*RPL22P1*	X
0.28	0.236	0.505	3.84E-221	3	*ANXA5*	X
0.31	0.302	0.55	8.94E-201	3	*IFITM1*	X
0.26	0.169	0.337	1.11E-110	3	*EPSTI1*	X

3.84	0.733	0.142	0	4	*SPINK4*	O
2.31	0.787	0.279	0	4	*WFDC2*	X
2.26	0.635	0.105	0	4	*FCGBP*	O
1.76	0.554	0.033	0	4	*KLK12*	O
1.53	0.529	0.029	0	4	*HEPACAM2*	O
1.11	0.658	0.336	5.22E-292	4	*ST6GALNAC1*	X
1.09	0.354	0.024	9.20E-251	4	*PCSK1*	O
0.85	0.538	0.084	2.35E-249	4	*LINC00261*	O
0.89	0.529	0.085	1.74E-246	4	*LRRC26*	O
0.78	0.623	0.181	7.60E-245	4	*KIAA1324*	O
0.8	0.391	0.026	6.44E-240	4	*REP15*	O
0.46	0.395	0.034	3.93E-236	4	*NEURL1*	O
1.1	0.677	0.208	2.32E-222	4	*ANXA13*	O
0.43	0.356	0.028	2.40E-206	4	*RAB26*	O
0.75	0.595	0.221	1.69E-198	4	*PLEKHB1*	O
1.09	0.729	0.359	1.80E-192	4	*SELENOM*	X
0.95	0.839	0.598	5.02E-191	4	*TSPAN13*	X
0.61	0.504	0.12	2.79E-189	4	*RAP1GAP*	O
0.79	0.672	0.426	5.79E-186	4	*IL13RA1*	X
0.78	0.729	0.393	1.76E-183	4	*SMIM14*	X
1.75	0.34	0.074	1.02E-179	4	*GUCA2A*	O
1.14	0.565	0.17	5.40E-178	4	*AQP3*	O
0.72	0.702	0.469	4.11E-169	4	*HPCAL1*	X
0.55	0.429	0.109	1.65E-160	4	*GAU1*	O
0.91	0.813	0.634	1.91E-156	4	*SEC11C*	X
2.15	0.473	0.122	2.55E-156	4	*MUC2*	O
1.26	0.656	0.396	3.30E-155	4	*KLK1*	X
0.55	0.408	0.08	8.54E-155	4	*SPDEF*	O
0.35	0.316	0.036	1.49E-152	4	*TMEM61*	O
0.64	0.39	0.066	8.42E-149	4	*TNFRSF11B*	O
1.35	0.428	0.201	9.07E-146	4	*TUBA1A*	O
2.5	0.632	0.261	2.55E-137	4	*REG4*	X
0.5	0.504	0.248	2.76E-128	4	*DYRK4*	O
0.35	0.298	0.047	2.95E-115	4	*COLCA1*	O
0.59	0.522	0.341	6.39E-112	4	*CMIP*	X
0.64	0.871	0.737	2.17E-109	4	*TPD52*	X
0.37	0.322	0.059	1.17E-106	4	*ENTPD8*	O
0.67	0.716	0.46	1.16E-105	4	*MYADM*	X
0.56	0.428	0.116	1.50E-104	4	*KLK11*	O
0.54	0.491	0.197	4.24E-99	4	*CREB3L1*	O
0.65	0.343	0.103	1.84E-93	4	*FABP2*	O
0.45	0.317	0.105	2.17E-93	4	*CDKAL1*	O
0.42	0.573	0.362	1.96E-91	4	*RANBP2*	X
0.38	0.406	0.137	8.00E-90	4	*PTPRN2*	O
0.87	0.847	0.631	9.60E-90	4	*PHLDA1*	X
0.36	0.452	0.167	1.80E-87	4	*HID1*	O
0.49	0.57	0.352	9.06E-83	4	*FOXA3*	X
0.53	0.805	0.675	5.32E-80	4	*TSTA3*	X
0.68	0.364	0.193	1.09E-79	4	*RASSF6*	O

0.33	0.333	0.093	2.22E-79	4	*PRUNE2*	O
0.48	0.743	0.515	1.39E-78	4	*FOXP1*	X
0.34	0.496	0.25	2.01E-76	4	*ARFGEF3*	X
0.35	0.322	0.101	1.71E-75	4	*ATP2A3*	O
0.35	0.352	0.114	3.19E-74	4	*DNAJC12*	O
0.77	0.347	0.323	1.08E-71	4	*AZGP1*	X
0.47	0.888	0.733	5.63E-71	4	*SCP2*	X
0.42	0.431	0.262	2.09E-69	4	*RBM38*	X
0.59	0.813	0.635	9.42E-67	4	*NPDC1*	X
0.36	0.333	0.139	1.22E-65	4	*ITPR2*	O
0.28	0.328	0.124	1.55E-63	4	*MAN1A1*	O
0.39	0.372	0.14	2.14E-63	4	*BCAS1*	O
0.37	0.487	0.272	5.00E-63	4	*WIPI1*	X
0.51	0.392	0.163	5.18E-62	4	*ALDH1A1*	O
0.36	0.608	0.426	2.52E-58	4	*PLPP5*	X
0.29	0.43	0.196	3.69E-58	4	*TMEM263*	O
0.39	0.62	0.426	5.21E-58	4	*CRACR2B*	X
0.7	0.31	0.15	1.05E-57	4	*NPW*	O
0.93	0.719	0.49	1.16E-57	4	*LYZ*	X
0.4	0.564	0.387	7.64E-57	4	*MYL6B*	X
0.43	0.739	0.607	1.36E-56	4	*PRXL2A*	X
0.48	0.824	0.685	7.23E-56	4	*TLE5*	X
0.36	0.526	0.325	2.95E-54	4	*CLRN3*	X
0.33	0.477	0.261	2.98E-54	4	*C3orf52*	X
0.28	0.418	0.229	2.43E-53	4	*HIPK2*	O
0.46	0.306	0.117	1.43E-52	4	*B3GNT7*	O
0.27	0.391	0.192	2.00E-51	4	*MAPRE2*	O
0.55	0.795	0.649	1.40E-50	4	*TGFBI*	X
0.46	0.594	0.37	1.07E-49	4	*RHOBTB3*	X
0.25	0.395	0.202	2.01E-48	4	*RAB15*	O
0.29	0.417	0.23	3.35E-47	4	*CCDC174*	O
0.3	0.549	0.394	3.46E-47	4	*DR1*	X
0.31	0.399	0.206	2.17E-46	4	*MAST4*	O
0.41	0.501	0.329	2.60E-46	4	*NUCB2*	X
0.28	0.28	0.136	9.36E-46	4	*PBXIP1*	O
0.36	0.609	0.467	1.49E-45	4	*ZNF428*	X
0.34	0.317	0.125	5.05E-45	4	*COL16A1*	O
0.41	0.509	0.345	1.43E-44	4	*TCEA3*	X
0.46	0.335	0.128	1.56E-44	4	*AQP5*	O
0.38	0.851	0.744	1.50E-43	4	*TSTD1*	X
0.53	0.509	0.292	2.46E-43	4	*DUSP4*	X
0.43	0.845	0.729	3.64E-43	4	*PPP1R14B*	X
0.37	0.598	0.419	2.66E-42	4	*KDM5B*	X
0.32	0.521	0.339	2.93E-42	4	*SLC9A3R2*	X
0.28	0.437	0.265	6.92E-42	4	*MIA3*	X
0.33	0.61	0.465	1.33E-41	4	*MAP7*	X
0.46	0.827	0.715	3.09E-41	4	*ETS2*	X
0.3	0.534	0.332	1.99E-40	4	*ABHD3*	X
0.38	0.837	0.727	2.00E-40	4	*SARAF*	X

0.32	0.353	0.212	8.13E-40	4	*UCP2*	O
0.3	0.592	0.446	2.03E-39	4	*AGO2*	X
0.31	0.634	0.505	5.40E-39	4	*UAP1*	X
0.25	0.445	0.241	5.55E-39	4	*MARCHF3*	O
0.29	0.605	0.461	6.95E-39	4	*SLC39A7*	X
0.26	0.455	0.301	1.60E-38	4	*GSE1*	X
0.29	0.539	0.396	3.06E-38	4	*IMPA2*	X
0.3	0.434	0.308	4.12E-38	4	*ODF2L*	X
0.26	0.466	0.258	6.60E-38	4	*MBOAT2*	X
0.31	0.631	0.501	8.29E-38	4	*PPP2R5C*	X
0.47	0.806	0.672	1.29E-37	4	*GADD45A*	X
0.28	0.577	0.421	2.28E-37	4	*ABLIM1*	X
0.26	0.505	0.368	2.41E-37	4	*DNAJC10*	X
0.3	0.572	0.432	9.87E-37	4	*CTNNBIP1*	X
0.36	0.754	0.659	1.51E-36	4	*ASPH*	X
0.28	0.492	0.341	1.71E-36	4	*TMCO3*	X
0.37	0.54	0.448	3.97E-36	4	*FKBP11*	X
0.29	0.64	0.504	1.31E-35	4	*ERGIC1*	X
0.3	0.456	0.259	3.03E-35	4	*DUSP10*	X
0.73	0.752	0.668	7.77E-35	4	*IGFBP2*	X
0.28	0.58	0.428	9.38E-35	4	*RHBDD2*	X
0.29	0.532	0.427	1.31E-34	4	*CKAP4*	X
0.32	0.484	0.339	2.07E-34	4	*HSD11B2*	X
0.34	0.574	0.451	2.59E-34	4	*PRKAR1A*	X
0.31	0.677	0.538	2.87E-34	4	*MSI2*	X
0.34	0.369	0.2	4.51E-34	4	*RND1*	O
0.33	0.834	0.728	6.45E-34	4	*TMED10*	X
0.28	0.712	0.582	7.07E-34	4	*TMEM134*	X
0.29	0.66	0.502	1.58E-33	4	*HMG20B*	X
0.28	0.639	0.503	1.63E-33	4	*ETNK1*	X
0.26	0.554	0.416	2.98E-33	4	*CLPTM1L*	X
0.26	0.427	0.314	4.79E-33	4	*MAD2L2*	X
0.31	0.615	0.482	4.85E-33	4	*ZKSCAN1*	X
0.29	0.584	0.456	6.14E-33	4	*MARCHF6*	X
0.29	0.589	0.401	6.54E-33	4	*KDM6B*	X
0.26	0.592	0.479	2.51E-32	4	*CDC34*	X
0.33	0.762	0.669	9.83E-32	4	*PIGT*	X
0.29	0.567	0.378	2.00E-31	4	*GPRC5C*	X
0.3	0.652	0.511	2.69E-31	4	*TRAPPC6A*	X
0.36	0.429	0.236	3.15E-31	4	*BEX3*	O
0.32	0.51	0.358	1.17E-30	4	*SH3BGRL*	X
0.29	0.588	0.465	1.22E-30	4	*AKIRIN1*	X
0.31	0.775	0.698	1.70E-30	4	*HDLBP*	X
0.29	0.697	0.543	1.90E-30	4	*ICA1*	X
0.27	0.294	0.177	2.71E-30	4	*PROX1*	O
0.25	0.503	0.362	2.82E-30	4	*SMCO4*	X
0.31	0.793	0.684	4.05E-30	4	*MPG*	X
0.28	0.434	0.266	4.56E-30	4	*ITGB8*	X
0.28	0.458	0.392	2.31E-29	4	*CRELD2*	X

0.32	0.621	0.472	4.84E-29	4	*YPEL5*	X
0.28	0.356	0.174	6.00E-29	4	*CRIP2*	O
0.28	0.802	0.727	1.20E-28	4	*TM9SF3*	X
0.25	0.302	0.241	2.33E-28	4	*ZFHX3*	O
0.28	0.497	0.341	1.43E-27	4	*ANG*	X
0.29	0.622	0.491	2.91E-27	4	*TMEM98*	X
0.3	0.538	0.441	9.73E-27	4	*AC027290.3*	X
0.37	0.732	0.607	3.06E-26	4	*MARCKS*	X
0.29	0.473	0.334	4.37E-26	4	*FRMD4B*	X
0.28	0.416	0.325	8.83E-25	4	*TMX4*	X
0.29	0.762	0.673	1.32E-24	4	*SEC62*	X
0.27	0.356	0.195	1.49E-24	4	*EGR3*	O
0.25	0.362	0.197	6.17E-24	4	*ST3GAL4*	O
0.34	0.636	0.566	2.30E-23	4	*SRP19*	X
0.35	0.769	0.728	2.53E-23	4	*PDIA4*	X
0.27	0.777	0.69	5.89E-23	4	*METTL9*	X
0.33	0.777	0.697	1.77E-22	4	*ITGA6*	X
0.26	0.552	0.44	3.75E-22	4	*TCEAL8*	X
0.33	0.838	0.706	6.54E-22	4	*TRIB1*	X
0.26	0.539	0.451	4.82E-21	4	*NEDD9*	X
0.27	0.67	0.571	1.29E-20	4	*SLC44A1*	X
0.25	0.633	0.545	1.46E-20	4	*RPAIN*	X
0.25	0.631	0.513	1.94E-20	4	*CYTH2*	X
0.26	0.561	0.446	4.82E-20	4	*WEE1*	X
0.28	0.551	0.449	8.34E-20	4	*VAMP2*	X
0.26	0.678	0.576	1.62E-18	4	*CMTM6*	X
0.25	0.731	0.637	3.23E-18	4	*TTC3*	X
0.31	0.763	0.671	5.61E-16	4	*TGIF1*	X
0.27	0.491	0.394	5.18E-14	4	*TUBB2A*	X
0.26	0.77	0.699	4.40E-12	4	*MANF*	X
0.28	0.728	0.634	2.73E-07	4	*HBEGF*	X
0.26	0.725	0.669	2.84E-07	4	*PNRC1*	X
0.26	0.714	0.657	0.000170758	4	*REEP5*	X
4.73	0.874	0.168	0	5	*MIR663AHG*	O
2.96	0.561	0.386	0	5	*TCIM*	X
2.75	0.824	0.61	0	5	*HES1*	X
2.66	0.662	0.403	0	5	*HEXIM1*	X
2.21	0.679	0.709	0	5	*RND3*	X
2.19	0.494	0.133	0	5	*LINC00910*	O
2.06	0.464	0.312	0	5	*CSKMT*	X
2.05	0.469	0.305	0	5	*PPP1R10*	X
2.04	0.603	0.611	0	5	*AC020916.1*	X
2	0.436	0.128	0	5	*CSRNP2*	O
1.91	0.598	0.726	0	5	*SON*	X
1.89	0.606	0.677	0	5	*TCF25*	X
1.72	0.609	0.748	0	5	*ARGLU1*	X
1.65	0.466	0.514	0	5	*AC103702.2*	X
1.59	0.436	0.558	0	5	*YME1L1*	X
1.56	0.374	0.296	0	5	*AHI1*	X

1.56	0.427	0.447	0	5	*RNF213*	X
1.55	0.346	0.283	0	5	*MED29*	X
1.52	0.369	0.375	0	5	*INTS6*	X
1.5	0.466	0.567	0	5	*SLC38A2*	X
1.41	0.304	0.295	0	5	*SNHG12*	X
1.37	0.444	0.513	0	5	*HOOK2*	X
1.32	0.352	0.483	0	5	*RSBN1L*	X
1.3	0.5	0.745	0	5	*BRD2*	X
1.26	0.293	0.293	0	5	*ILF3-DT*	X
1.26	0.455	0.703	0	5	*DDX3X*	X
1.03	0.243	0.33	0	5	*EIF4A1*	X
0.82	0.341	0.669	1.77e-318	5	*ILF3*	X
0.85	0.226	0.43	4.24e-313	5	*TRIM44*	X
1.27	0.33	0.48	6.61e-311	5	*THUMPD3-AS1*	X
0.77	0.223	0.462	2.50E-307	5	*TMEM259*	X
2.37	0.427	0.115	1.43E-305	5	*AC239799.2*	O
1.62	0.324	0.182	4.33E-305	5	*AC018521.5*	O
1.37	0.293	0.398	3.28E-303	5	*PLK2*	X
0.82	0.168	0.331	3.67E-303	5	*POLR2A*	X
0.9	0.196	0.343	2.43E-300	5	*DIAPH2*	X
1.02	0.291	0.425	1.63E-298	5	*C1orf35*	X
0.8	0.218	0.429	3.23E-298	5	*CDK12*	X
1.04	0.33	0.592	2.47E-297	5	*ANKRD11*	X
0.72	0.285	0.621	5.32E-294	5	*PABPC4*	X
0.93	0.249	0.412	7.76E-293	5	*PRPF4B*	X
2.77	0.564	0.065	2.62E-291	5	*AL021155.4*	O
0.83	0.302	0.635	1.35E-289	5	*AMD1*	X
0.9	0.226	0.413	4.44E-288	5	*CCNL2*	X
0.76	0.176	0.307	1.13E-287	5	*SCAMP4*	X
0.93	0.251	0.49	5.25E-286	5	*GOLGB1*	X
0.78	0.19	0.414	6.89E-286	5	*NUFIP2*	X
4.03	0.67	0.129	9.28E-286	5	*ANKRD30BL*	O
0.86	0.268	0.536	3.10E-285	5	*DDX24*	X
0.92	0.257	0.423	5.00E-285	5	*BICDL2*	X
0.82	0.187	0.318	5.13E-285	5	*RSBN1*	X
0.79	0.218	0.361	6.86E-280	5	*CHD7*	X
2.7	0.517	0.075	7.17E-279	5	*Z93241.1*	O
0.91	0.243	0.396	2.53E-278	5	*RHOT2*	X
0.88	0.313	0.598	4.74E-273	5	*PNN*	X
0.86	0.372	0.726	1.04E-268	5	*PRRC2C*	X
0.63	0.117	0.308	6.01E-268	5	*MYO1E*	X
2.13	0.707	0.713	1.34E-267	5	*FOSB*	X
1.17	0.321	0.404	3.65E-267	5	*NFKBIZ*	X
0.97	0.274	0.477	1.07E-266	5	*ABCC3*	X
0.91	0.218	0.321	3.16E-266	5	*CDK10*	X
0.71	0.282	0.589	5.74E-265	5	*RTF1*	X
1.28	0.302	0.289	1.43E-264	5	*WDR60*	X
0.75	0.198	0.43	1.22E-263	5	*INF2*	X
0.63	0.182	0.431	1.91E-263	5	*WDR74*	X

0.66	0.204	0.457	1.95E-263	5	*ASXL1*	X
1.11	0.277	0.373	1.12E-261	5	*ANO9*	X
0.51	0.193	0.561	1.42E-261	5	*RBBP6*	X
0.87	0.288	0.488	1.86E-261	5	*EPS8L2*	X
0.83	0.344	0.66	2.83E-260	5	*PHF14*	X
0.86	0.204	0.292	1.01E-259	5	*NOM1*	X
2.47	0.397	0.108	8.44E-255	5	*AC109326.1*	O
1.05	0.277	0.353	1.83E-254	5	*UPF2*	X
0.61	0.209	0.488	5.25E-253	5	*DHX36*	X
0.62	0.184	0.45	1.20E-252	5	*TRABD*	X
0.84	0.265	0.483	1.63E-252	5	*LINC00511*	X
0.51	0.201	0.579	1.67E-252	5	*TRIM28*	X
2	0.679	0.661	5.29E-252	5	*ID2*	X
0.66	0.237	0.547	8.29E-252	5	*ATRX*	X
0.65	0.296	0.672	2.61E-249	5	*DDX18*	X
0.73	0.162	0.348	3.21E-249	5	*CEMIP2*	X
0.72	0.24	0.533	1.15E-248	5	*STAT3*	X
0.67	0.209	0.457	2.60E-248	5	*IRF3*	X
1.05	0.257	0.379	3.31E-248	5	*AC015912.3*	X
0.88	0.321	0.57	1.98E-247	5	*MPHOSPH8*	X
0.79	0.307	0.676	3.49E-247	5	*ACADVL*	X
0.61	0.226	0.598	1.04E-246	5	*KMT2E*	X
0.65	0.154	0.351	1.54E-245	5	*PNKP*	X
0.38	0.109	0.347	9.25E-245	5	*ZNF598*	X
0.54	0.212	0.547	1.84E-244	5	*AFDN*	X
0.69	0.187	0.34	9.18E-244	5	*BDP1*	X
0.57	0.142	0.348	1.04E-242	5	*SPEN*	X
0.6	0.196	0.526	1.53E-239	5	*DNM2*	X
0.69	0.168	0.318	2.33E-239	5	*NFATC2IP*	X
0.44	0.265	0.708	2.77E-239	5	*ACTR2*	X
0.58	0.221	0.57	7.89E-239	5	*DYNC1H1*	X
1.83	0.497	0.473	1.75E-238	5	*GADD45B*	X
0.71	0.207	0.452	3.08E-238	5	*LUZP1*	X
0.37	0.232	0.689	4.89E-238	5	*METAP2*	X
0.4	0.14	0.475	5.33E-238	5	*EML4*	X
0.49	0.168	0.504	8.77E-237	5	*CSNK1D*	X
0.59	0.277	0.623	1.04E-236	5	*NCOR1*	X
0.68	0.299	0.693	4.46E-236	5	*TRA2B*	X
1	0.341	0.611	6.06E-236	5	*N4BP2L2*	X
0.58	0.251	0.589	4.70E-235	5	*CHCHD7*	X
0.76	0.187	0.372	8.77E-235	5	*RBM6*	X
1.02	0.24	0.336	1.98E-234	5	*AL118516.1*	X
0.46	0.184	0.52	2.81E-234	5	*ABCF1*	X
0.59	0.128	0.281	3.03E-233	5	*DALRD3*	X
0.62	0.265	0.621	7.29E-233	5	*DPP7*	X
0.6	0.148	0.257	8.78E-232	5	*CPSF1*	X
0.69	0.246	0.549	2.51E-231	5	*ASL*	X
0.61	0.24	0.598	2.92E-231	5	*UGP2*	X
0.42	0.112	0.4	3.42E-231	5	*AKT1S1*	X

0.86	0.257	0.398	1.13E-229	5	*KNOP1*	X
0.54	0.17	0.415	1.62E-229	5	*ATF6B*	X
1.87	0.318	0.058	3.83E-229	5	*DLL4*	O
1.84	0.299	0.103	4.83E-229	5	*AC253572.2*	O
0.59	0.198	0.511	1.72E-228	5	*EPS8L3*	X
0.54	0.126	0.301	2.67E-228	5	*HMGXB4*	X
0.48	0.198	0.554	2.82E-228	5	*SPTAN1*	X
0.48	0.249	0.647	5.25E-228	5	*RBM25*	X
0.58	0.282	0.672	5.89E-228	5	*EIF3J*	X
0.49	0.109	0.35	2.65E-226	5	*FRYL*	X
0.57	0.168	0.346	5.50E-226	5	*CSNK1G2*	X
0.4	0.098	0.282	5.73E-226	5	*CAPN15*	X
0.66	0.237	0.527	9.33E-226	5	*NSD3*	X
0.84	0.182	0.283	1.22E-225	5	*SNHG9*	X
0.83	0.268	0.464	1.61E-225	5	*OFD1*	X
1.77	0.349	0.192	2.51E-225	5	*AC023157.2*	O
1.28	0.268	0.2	1.63E-224	5	*TLE4*	O
0.42	0.229	0.626	1.76E-224	5	*CCDC12*	X
0.95	0.246	0.327	2.98E-224	5	*MYLIP*	X
0.5	0.123	0.3	5.46E-224	5	*FGFR4*	X
0.5	0.223	0.586	5.82E-224	5	*THOC2*	X
0.71	0.299	0.656	9.00E-224	5	*TAF7*	X
0.73	0.232	0.502	1.79E-223	5	*RNF145*	X
0.65	0.145	0.275	4.88E-223	5	*PLXNB1*	X
0.36	0.207	0.663	2.28E-222	5	*ERBB3*	X
0.58	0.363	0.734	1.04E-219	5	*ARL6IP4*	X
0.42	0.151	0.481	1.62E-219	5	*TOB2*	X
0.48	0.176	0.535	3.26E-219	5	*TPR*	X
0.82	0.31	0.597	3.57E-219	5	*LUC7L3*	X
0.53	0.14	0.362	7.82E-219	5	*VARS1*	X
0.62	0.156	0.31	3.02E-218	5	*TRMT1*	X
0.46	0.117	0.391	1.86E-217	5	*SPG7*	X
0.39	0.223	0.66	5.96E-217	5	*SNRNP70*	X
2.86	0.458	0.062	2.45E-216	5	*AL355075.4*	O
0.58	0.176	0.456	2.63E-216	5	*PLXNB2*	X
0.98	0.249	0.403	6.80E-216	5	*RSRP1*	X
0.43	0.159	0.49	1.39E-215	5	*NFIA*	X
0.52	0.151	0.449	6.64E-215	5	*SPAG9*	X
2.6	0.416	0.06	3.22E-214	5	*MAFB*	O
0.57	0.165	0.434	3.72E-214	5	*H2AW*	X
0.32	0.229	0.683	6.04E-214	5	*RNH1*	X
0.4	0.187	0.566	1.66E-213	5	*U2SURP*	X
0.41	0.159	0.498	8.08E-213	5	*CSNK1A1*	X
0.44	0.123	0.352	9.34E-213	5	*GSK3B*	X
0.38	0.123	0.43	9.47E-213	5	*INTS11*	X
0.41	0.221	0.615	5.55E-212	5	*MRPS7*	X
0.33	0.176	0.568	2.05E-211	5	*BRD4*	X
0.4	0.151	0.473	2.77E-211	5	*NARF*	X
0.36	0.115	0.397	3.22E-211	5	*FASTK*	X

0.32	0.249	0.717	6.97E-211	5	*SYF2*	X
0.44	0.14	0.409	1.05E-210	5	*TDG*	X
0.63	0.19	0.432	2.61E-210	5	*SUPT5H*	X
0.3	0.254	0.734	5.71E-210	5	*NORAD*	X
0.51	0.137	0.327	5.71E-210	5	*MYCBP2*	X
0.31	0.179	0.563	5.86E-210	5	*AP3D1*	X
0.61	0.296	0.719	7.27E-210	5	*CD44*	X
0.47	0.187	0.508	8.44E-210	5	*BAZ1A*	X
0.63	0.145	0.265	9.04E-210	5	*ARHGEF1*	X
0.43	0.249	0.633	1.04E-209	5	*NT5C*	X
0.53	0.168	0.405	1.01E-208	5	*AFF4*	X
0.72	0.288	0.621	4.44E-208	5	*PNISR*	X
0.3	0.17	0.586	4.68E-208	5	*SF3B1*	X
0.45	0.142	0.386	4.97E-208	5	*MAP3K11*	X
0.36	0.106	0.352	5.13E-208	5	*SLC38A10*	X
0.7	0.17	0.281	5.75E-208	5	*CHD6*	X
0.47	0.165	0.436	1.83E-207	5	*PHIP*	X
0.38	0.165	0.476	2.36E-206	5	*BPTF*	X
0.71	0.288	0.577	2.63E-206	5	*MYO6*	X
0.47	0.128	0.366	3.05E-206	5	*PVT1*	X
0.64	0.243	0.535	3.18E-206	5	*PPP1R16A*	X
0.37	0.123	0.407	4.31E-206	5	*VPS36*	X
0.65	0.249	0.537	7.03E-206	5	*EPB41L2*	X
0.34	0.198	0.601	1.79E-205	5	*ZBTB7A*	X
0.37	0.142	0.438	2.25E-205	5	*HGS*	X
0.27	0.168	0.622	2.28E-205	5	*GOLGA4*	X
0.57	0.288	0.672	2.96E-205	5	*ABHD11*	X
0.27	0.106	0.412	3.26E-205	5	*ARL16*	X
0.63	0.165	0.288	1.32E-204	5	*COQ7*	X
0.36	0.087	0.292	3.51E-204	5	*RBM10*	X
0.39	0.173	0.47	6.14E-204	5	*SNRNP200*	X
0.54	0.218	0.547	1.27E-203	5	*GALE*	X
0.49	0.148	0.407	1.31E-203	5	*PKN2*	X
1.57	0.299	0.081	1.36E-203	5	*H2BC15*	O
0.41	0.274	0.703	1.41E-203	5	*TMEM160*	X
0.44	0.168	0.478	1.54E-203	5	*FNBP4*	X
0.44	0.212	0.564	3.18E-203	5	*DYNC1I2*	X
0.46	0.126	0.342	1.81E-202	5	*KDM5A*	X
0.46	0.187	0.549	3.71E-202	5	*TRA2A*	X
0.77	0.182	0.265	3.72E-202	5	*SPPL2B*	X
0.51	0.128	0.322	4.75E-202	5	*USP34*	X
0.58	0.14	0.292	5.48E-202	5	*ARFGAP1*	X
0.66	0.159	0.258	1.00E-201	5	*GPATCH2*	X
0.62	0.151	0.316	1.09E-201	5	*RUNX1*	X
0.37	0.112	0.399	1.13E-201	5	*DDX56*	X
0.48	0.126	0.333	1.25E-201	5	*LONP1*	X
0.38	0.221	0.608	1.54E-201	5	*DNAJC2*	X
0.63	0.212	0.419	2.15E-201	5	*TPGS1*	X
0.75	0.218	0.454	3.01E-201	5	*PLK3*	X

0.39	0.19	0.561	4.91E-201	5	*ENTPD6*	X
1.2	0.254	0.167	7.45E-201	5	*CUTALP*	O
0.33	0.095	0.324	7.85E-201	5	*PUS1*	X
0.39	0.145	0.422	1.71E-200	5	*GTF2F1*	X
0.4	0.087	0.287	7.77E-200	5	*MECOM*	X
0.53	0.145	0.371	3.94E-199	5	*ZRANB2*	X
0.26	0.131	0.511	1.20E-198	5	*ADGRE5*	X
0.59	0.137	0.299	2.15E-198	5	*PCF11*	X
0.51	0.173	0.475	2.88E-198	5	*TMEM63A*	X
0.32	0.095	0.394	2.06E-197	5	*GAK*	X
0.5	0.156	0.371	2.91E-197	5	*EPC1*	X
0.55	0.279	0.605	3.08E-197	5	*COMTD1*	X
2.09	0.391	0.055	7.37E-197	5	*AP001160.1*	O
0.38	0.162	0.502	8.45E-197	5	*AP2B1*	X
0.52	0.154	0.354	1.80E-196	5	*RERE*	X
0.47	0.115	0.304	2.06E-196	5	*USP36*	X
0.54	0.19	0.478	5.69E-196	5	*ESF1*	X
0.31	0.12	0.455	1.20E-195	5	*PTPN12*	X
0.45	0.165	0.467	6.63E-195	5	*NKTR*	X
0.54	0.126	0.268	5.16E-194	5	*SYMPK*	X
0.34	0.131	0.397	1.23E-193	5	*SIAH2*	X
0.88	0.204	0.323	6.99E-193	5	*CHD9*	X
0.32	0.196	0.581	2.87E-192	5	*FAM133B*	X
0.52	0.207	0.518	1.11E-191	5	*RASSF7*	X
0.41	0.115	0.358	1.12E-191	5	*KMT2C*	X
0.49	0.176	0.419	1.80E-191	5	*FNIP1*	X
0.62	0.154	0.344	5.60E-191	5	*RNMT*	X
0.31	0.095	0.279	9.51E-191	5	*PURA*	X
0.36	0.092	0.317	1.06E-190	5	*SLC4A2*	X
0.36	0.201	0.616	1.67E-190	5	*MVP*	X
0.41	0.098	0.293	1.76E-190	5	*NADSYN1*	X
0.33	0.089	0.335	3.11E-190	5	*TACC2*	X
0.36	0.098	0.362	4.04E-190	5	*RTKN*	X
0.28	0.064	0.286	4.37E-190	5	*SEC16A*	X
0.5	0.128	0.29	4.76E-190	5	*CNOT4*	X
0.44	0.168	0.476	6.93E-190	5	*ARID4B*	X
0.36	0.103	0.304	2.68E-189	5	*REXO4*	X
0.42	0.109	0.296	5.72E-189	5	*LUC7L*	X
0.4	0.089	0.274	6.09E-189	5	*NAT9*	X
0.3	0.106	0.426	2.54E-188	5	*JOSD1*	X
0.49	0.131	0.304	2.69E-188	5	*SLC25A37*	X
3.24	0.441	0.021	4.74E-188	5	*RN7SK*	O
0.36	0.117	0.34	9.33E-188	5	*TGS1*	X
0.39	0.137	0.399	1.04E-187	5	*SAFB2*	X
0.37	0.101	0.291	1.64E-187	5	*DDX23*	X
0.98	0.316	0.449	1.88E-187	5	*IER5*	X
0.46	0.117	0.313	3.58E-187	5	*BAZ2B*	X
0.56	0.126	0.257	4.22E-187	5	*NXF1*	X
0.52	0.151	0.401	8.69E-187	5	*TLE1*	X

0.38	0.151	0.453	4.67E-186	5	*BRD7*	X
0.58	0.131	0.253	5.49E-186	5	*GON4L*	X
0.46	0.168	0.447	5.93E-186	5	*ZNF622*	X
0.28	0.126	0.475	1.15E-185	5	*POF1B*	X
0.67	0.31	0.611	2.54E-185	5	*ARL4A*	X
0.33	0.081	0.347	4.95E-185	5	*TNFRSF10B*	X
0.3	0.123	0.447	5.90E-185	5	*PPP1R15B*	X
0.78	0.335	0.681	8.48E-184	5	*LDLR*	X
0.36	0.098	0.263	9.84E-184	5	*TRIM11*	X
0.33	0.128	0.437	2.02E-183	5	*GLS*	X
0.49	0.327	0.715	4.54E-183	5	*CCDC85B*	X
0.44	0.151	0.453	6.70E-183	5	*KCNN4*	X
0.43	0.128	0.313	3.46E-182	5	*ABR*	X
0.31	0.123	0.419	3.53E-182	5	*SMG1*	X
0.35	0.142	0.45	6.58E-182	5	*DGAT1*	X
0.34	0.089	0.342	1.00E-181	5	*SLC12A7*	X
0.49	0.148	0.379	1.19E-181	5	*RNF43*	X
0.3	0.126	0.477	3.50E-181	5	*SUDS3*	X
0.28	0.179	0.583	4.70E-181	5	*GNL3*	X
0.32	0.117	0.398	4.90E-181	5	*TSC22D2*	X
0.37	0.128	0.424	2.36E-180	5	*BBX*	X
0.25	0.087	0.353	3.90E-180	5	*MAN1A2*	X
0.26	0.117	0.453	4.35E-180	5	*SREK1*	X
0.45	0.098	0.263	4.68E-180	5	*BRAT1*	X
0.33	0.101	0.369	1.28E-179	5	*KANSL1*	X
0.44	0.112	0.313	1.59E-179	5	*C19orf25*	X
0.53	0.14	0.299	1.87E-179	5	*KAT2A*	X
0.35	0.14	0.455	4.86E-179	5	*TRMT10C*	X
0.33	0.123	0.394	7.98E-179	5	*UCKL1*	X
0.28	0.087	0.33	1.35E-178	5	*PDCD7*	X
0.31	0.103	0.349	1.76E-178	5	*NSMF*	X
0.42	0.103	0.257	4.00E-178	5	*HCG18*	X
0.28	0.092	0.35	7.74E-178	5	*KIAA1191*	X
0.32	0.165	0.511	7.87E-178	5	*HP1BP3*	X
0.45	0.106	0.279	1.12E-177	5	*ITSN2*	X
0.26	0.151	0.544	1.90E-177	5	*NME3*	X
0.79	0.19	0.376	4.53E-177	5	*ATP10B*	X
0.29	0.101	0.38	9.39E-177	5	*LARP1B*	X
0.42	0.137	0.4	3.00E-176	5	*NEMF*	X
0.3	0.117	0.414	3.52E-176	5	*ADAP1*	X
0.4	0.106	0.302	1.27E-175	5	*PCM1*	X
0.37	0.14	0.384	1.82E-175	5	*CEBPZ*	X
0.3	0.12	0.423	1.87E-175	5	*SRPK2*	X
0.39	0.145	0.414	2.57E-175	5	*SIRT7*	X
0.39	0.123	0.358	5.70E-175	5	*ASH1L*	X
0.45	0.128	0.355	6.93E-175	5	*KHDC4*	X
0.26	0.123	0.501	1.23E-174	5	*VCL*	X
0.33	0.168	0.552	2.04E-174	5	*EHF*	X
0.63	0.162	0.393	4.58E-174	5	*BNIP5*	X

0.51	0.145	0.334	1.93E-173	5	*CWC25*	X
0.27	0.142	0.49	3.20E-173	5	*TBRG4*	X
0.54	0.142	0.301	1.63E-172	5	*ERICH1*	X
0.28	0.193	0.594	1.75E-172	5	*FAM50A*	X
0.43	0.109	0.266	6.66E-172	5	*SLC26A6*	X
0.46	0.131	0.32	3.13E-171	5	*SMTN*	X
0.34	0.106	0.313	3.60E-171	5	*FAHD1*	X
0.45	0.159	0.428	4.33E-171	5	*SPSB3*	X
0.98	0.229	0.302	1.32E-170	5	*CITED2*	X
0.4	0.128	0.399	2.13E-170	5	*LENG8*	X
0.34	0.106	0.345	2.66E-170	5	*ANKLE2*	X
0.28	0.081	0.283	5.62E-170	5	*CDYL*	X
0.4	0.131	0.413	5.25E-169	5	*MAT2A*	X
0.64	0.198	0.411	5.26E-169	5	*CEBPA*	X
0.45	0.159	0.414	2.62E-168	5	*MIB2*	X
0.36	0.106	0.319	3.59E-168	5	*HNRNPL*	X
0.41	0.092	0.273	3.81E-168	5	*AKAP8L*	X
0.28	0.109	0.41	7.79E-168	5	*TNFRSF14*	X
0.75	0.327	0.62	1.70E-167	5	*CEBPD*	X
0.35	0.131	0.378	4.80E-167	5	*SH2D3A*	X
0.3	0.089	0.301	1.63E-166	5	*ZNF394*	X
0.35	0.101	0.404	1.82E-166	5	*SLC7A5*	X
0.29	0.075	0.269	5.45E-166	5	*MED16*	X
0.47	0.126	0.33	1.11E-165	5	*SYNE2*	X
0.46	0.106	0.258	3.89E-165	5	*ARHGEF16*	X
0.26	0.101	0.426	4.01E-165	5	*DAPK3*	X
0.31	0.095	0.282	5.17E-165	5	*AUTS2*	X
0.26	0.064	0.262	6.95E-165	5	*RREB1*	X
0.3	0.084	0.259	6.98E-165	5	*ZC3H18*	X
0.51	0.12	0.251	7.25E-165	5	*TSC2*	X
0.49	0.123	0.282	8.98E-165	5	*NMT1*	X
0.29	0.098	0.345	1.81E-164	5	*UBR5*	X
0.36	0.101	0.335	2.86E-164	5	*SLC20A2*	X
0.32	0.106	0.329	6.45E-164	5	*LMBRD1*	X
0.42	0.151	0.4	1.67E-163	5	*NSRP1*	X
0.3	0.134	0.479	1.70E-163	5	*AKAP13*	X
0.3	0.128	0.403	2.72E-163	5	*RNPEPL1*	X
0.45	0.123	0.252	3.21E-163	5	*FAM53C*	X
0.37	0.092	0.285	9.34E-163	5	*SPATA13*	X
0.33	0.084	0.288	1.16E-162	5	*INTS1*	X
0.26	0.078	0.315	1.73E-162	5	*EEA1*	X
0.41	0.098	0.251	1.78E-162	5	*CDC42BPA*	X
0.28	0.12	0.392	1.55E-161	5	*YIF1B*	X
0.29	0.12	0.401	4.48E-161	5	*ARHGAP27*	X
0.39	0.101	0.255	4.59E-161	5	*USP15*	X
0.28	0.109	0.365	3.71E-160	5	*TCERG1*	X
0.31	0.123	0.404	1.21E-159	5	*TMC4*	X
0.31	0.087	0.281	4.36E-159	5	*PUS7L*	X
0.34	0.17	0.527	4.38E-159	5	*CLK1*	X

0.36	0.101	0.298	9.86E-159	5	*RBM5*	X
0.37	0.176	0.489	1.08E-158	5	*C8orf33*	X
0.37	0.106	0.338	2.91E-158	5	*PHYH*	X
0.29	0.098	0.356	5.19E-158	5	*UBAP2*	X
0.55	0.24	0.591	3.28E-157	5	*PMAIP1*	X
0.33	0.109	0.334	6.86E-157	5	*UBALD1*	X
0.35	0.106	0.319	2.46E-156	5	*KIF16B*	X
0.37	0.112	0.337	2.55E-156	5	*CBX4*	X
0.29	0.092	0.32	4.58E-156	5	*TBRG1*	X
0.35	0.109	0.321	1.45E-155	5	*CCDC14*	X
0.3	0.092	0.268	3.46E-155	5	*FAAH*	X
0.45	0.115	0.332	1.51E-154	5	*CAPN8*	X
0.3	0.084	0.28	9.40E-154	5	*NUP62*	X
0.36	0.12	0.355	1.67E-153	5	*ASPSCR1*	X
0.25	0.07	0.26	1.81E-153	5	*LRBA*	X
0.28	0.078	0.267	3.18E-153	5	*ATXN7L3B*	X
0.34	0.084	0.262	4.19E-153	5	*SFSWAP*	X
0.34	0.103	0.281	3.62E-152	5	*NUDCD3*	X
0.56	0.212	0.422	8.08E-152	5	*POLR2J3*	X
0.62	0.383	0.727	1.67E-151	5	*CEBPB*	X
0.39	0.087	0.25	6.48E-151	5	*CDKN2AIP*	X
0.28	0.095	0.317	5.05E-150	5	*SRRT*	X
0.37	0.101	0.262	7.73E-150	5	*EHMT1*	X
0.26	0.176	0.512	1.63E-149	5	*JOSD2*	X
0.28	0.089	0.326	2.52E-149	5	*HERC4*	X
0.4	0.115	0.275	5.97E-149	5	*ELMO3*	X
0.34	0.112	0.344	3.55E-148	5	*OGT*	X
0.27	0.123	0.401	3.59E-148	5	*POLE3*	X
0.42	0.128	0.286	5.05E-148	5	*SPIRE2*	X
0.27	0.089	0.29	5.76E-148	5	*TRIM8*	X
0.51	0.123	0.33	6.81E-148	5	*ITPKC*	X
0.28	0.075	0.255	1.02E-146	5	*R3HDM2*	X
2.11	0.36	0.032	1.96E-146	5	*AC241584.1*	O
0.52	0.145	0.296	6.72E-146	5	*FUT2*	X
1.29	0.257	0.144	8.88E-146	5	*GADD45G*	O
0.48	0.168	0.348	2.35E-145	5	*CC2D1A*	X
0.25	0.128	0.439	7.12E-145	5	*OTUD6B-AS1*	X
0.43	0.117	0.256	7.30E-145	5	*RC3H1*	X
0.43	0.123	0.25	9.61E-145	5	*FOXA2*	X
0.26	0.084	0.308	3.06E-144	5	*PHF3*	X
0.28	0.092	0.268	1.95E-143	5	*KIAA2026*	X
0.59	0.168	0.344	5.25E-142	5	*MYO15B*	X
0.4	0.145	0.348	5.94E-142	5	*SNHG19*	X
0.42	0.101	0.263	6.71E-142	5	*NABP1*	X
0.29	0.209	0.577	4.61E-141	5	*NAPRT*	X
0.27	0.092	0.335	1.20E-138	5	*MPHOSPH10*	X
0.3	0.151	0.438	2.61E-137	5	*HOXB7*	X
0.28	0.098	0.33	3.85E-137	5	*MAP4K3-DT*	X
0.27	0.12	0.38	4.02E-135	5	*RSRC1*	X

0.26	0.078	0.271	8.21E-135	5	*CD3EAP*	X
0.29	0.12	0.375	6.07E-134	5	*ANKRD12*	X
0.28	0.154	0.456	1.56E-132	5	*FASN*	X
0.27	0.12	0.34	6.79E-132	5	*TRIM56*	X
0.63	0.159	0.274	1.53E-131	5	*IRF7*	X
0.28	0.078	0.259	2.32E-130	5	*WDR6*	X
0.37	0.117	0.411	2.45E-130	5	*ENC1*	X
0.28	0.089	0.29	3.54E-129	5	*ATG2A*	X
0.28	0.112	0.32	3.79E-129	5	*PPAN*	X
0.66	0.173	0.307	1.25E-128	5	*FOXQ1*	X
2.56	0.279	0.04	7.40E-127	5	*CCDC80*	O
0.26	0.26	0.654	1.18E-126	5	*S100A13*	X
0.3	0.081	0.251	6.56E-126	5	*ZNF638*	X
0.73	0.198	0.324	2.42E-124	5	*TRPM4*	X
0.42	0.134	0.299	2.67E-124	5	*ZNF503*	X
0.37	0.109	0.292	8.17E-124	5	*ARID5B*	X
0.3	0.095	0.275	1.59E-123	5	*PWWP3A*	X
0.3	0.081	0.251	1.28E-122	5	*RAD9A*	X
0.29	0.089	0.307	1.34E-120	5	*TP53I3*	X
0.37	0.137	0.327	8.52E-117	5	*FTX*	X
0.25	0.078	0.3	9.84E-117	5	*RASEF*	X
0.33	0.159	0.426	8.83E-116	5	*PLCB4*	X
0.29	0.112	0.303	1.12E-115	5	*PGM2L1*	X
0.25	0.117	0.37	3.05E-115	5	*SVIL*	X
1.89	0.299	0.065	1.25E-114	5	*AD000090.1*	O
0.43	0.123	0.367	2.57E-114	5	*FLNA*	X
0.31	0.089	0.263	2.36E-110	5	*MTMR11*	X
0.28	0.106	0.284	1.77E-108	5	*TBCC*	X
0.27	0.109	0.326	3.44E-106	5	*HELZ2*	X
1.74	0.251	0.026	2.77E-100	5	*MIR3648*	O
0.58	0.204	0.492	5.19E-99	5	*ADM*	X
0.39	0.14	0.283	3.76E-97	5	*MT-ATP8*	X
0.28	0.106	0.271	1.18E-94	5	*LTBP3*	X
0.31	0.162	0.414	4.64E-94	5	*HLA-F*	X
0.3	0.109	0.272	2.18E-89	5	*EPHB3*	X
0.53	0.193	0.397	6.41E-89	5	*DDIT3*	X
0.3	0.17	0.411	4.42E-80	5	*TCEAL9*	X
0.34	0.24	0.519	3.81E-76	5	*AREG*	X
0.33	0.106	0.264	7.56E-76	5	*HILPDA*	X
0.41	0.436	0.711	5.35E-75	5	*DUSP1*	X
0.61	0.193	0.266	7.85E-65	5	*IGHG3*	X
0.64	0.201	0.288	5.84E-63	5	*IGHG4*	X
1.07	0.268	0.285	9.96E-61	5	*PCK1*	X
0.54	0.486	0.658	1.04E-41	5	*ID1*	X
4.76	0.638	0.022	1.10E-258	6	*REG1B*	O
4.51	0.627	0.033	4.71E-227	6	*REG3A*	O
1.14	0.556	0.11	4.54E-64	6	*DMBT1*	O
0.94	0.937	0.62	2.02E-40	6	*CD74*	X
0.86	0.272	0.065	8.93E-39	6	*ADH1C*	O

0.85	0.776	0.385	7.41E-34	6	*HLA-DRA*	X
0.38	0.429	0.141	5.19E-24	6	*MUC4*	O
0.55	0.362	0.113	1.28E-23	6	*SMOC2*	O
0.39	0.437	0.215	3.75E-19	6	*CA12*	O
0.27	0.455	0.188	5.24E-18	6	*MAP2K6*	O
0.61	0.422	0.241	1.61E-15	6	*PCCA*	O
0.85	0.511	0.382	1.29E-14	6	*BAG3*	X
0.3	0.347	0.128	1.59E-14	6	*RARRES1*	O
0.29	0.47	0.218	3.85E-14	6	*CASP1*	O
0.41	0.899	0.731	2.20E-13	6	*CYB5A*	X
0.46	0.817	0.637	1.06E-12	6	*SOD2*	X
0.42	0.526	0.289	1.06E-11	6	*HLA-DMA*	X
0.47	0.392	0.314	4.24E-11	6	*TOX3*	X
0.69	0.489	0.254	6.68E-11	6	*RPS4Y1*	X
0.34	0.343	0.165	7.95E-10	6	*RGMB*	O
0.33	0.388	0.236	1.53E-09	6	*MAOA*	O
0.25	0.586	0.359	2.49E-08	6	*UBE2L6*	X
0.33	0.888	0.742	2.57E-08	6	*PSMB8*	X
0.31	0.362	0.328	8.25E-08	6	*DNAJA4*	X
0.36	0.433	0.23	1.24E-07	6	*HLA-DPA1*	O
0.27	0.664	0.475	3.02E-07	6	*GAS6*	X
0.32	0.265	0.129	1.19E-06	6	*DNAJB4*	O
0.26	0.381	0.266	2.12E-06	6	*TENT4B*	X
0.28	0.858	0.707	2.97E-06	6	*MGST2*	X
0.27	0.507	0.321	1.48E-05	6	*AKR1C3*	X
0.31	0.616	0.448	3.37E-05	6	*GOLIM4*	X
0.31	0.351	0.229	3.84E-05	6	*PTP4A3*	O
0.29	0.369	0.206	0.0002	6	*HLA-DPB1*	O
0.3	0.396	0.237	0.00021	6	*CCN1*	O
0.27	0.653	0.53	0.00026	6	*SQOR*	X
0.25	0.828	0.697	0.0015	6	*ALDH2*	X
0.25	0.705	0.583	0.01	6	*AHSA1*	X
