# Supplementary material for: Clustering malignant cell states using universally variable genes
Source: Brief Bioinform. 2023 Dec 11;25(1):bbad460. doi: 10.1093/bib/bbad460 (PMC10783859; doi:10.1093/bib/bbad460)
Supplement: Suppmentary_Table_2_bbad460 [file suppmentary_table_2_bbad460.pdf]

Cancer type	Reference	Platform	Public accession #	Patient #
BRCA	*Sunny Wu et al., Nature Genetics (2021)*	10X 3'-seq	GSE176078 (TNBC)	8
		10X 3'-seq	GSE176078 (ER+)	9
		10X 3'-seq	GSE176078 (HER2+)	3
	Junbin Qian et al. Cell Research (2020)	10X 5'-seq	http://blueprint.lambrechtslab.org	14
CRC	*Hae-Ock Lee et al., Nature Genetics (2020)*	10X 3'-seq	EGAD00001005198	22
	Hae-Ock Lee et al., Nature Genetics (2020)	10X 3'-seq	E-MTAB-8410	9
	Junbin Qian et al. Cell Research (2020)	10X 3'-seq	http://blueprint.lambrechtslab.org	7
	Ignasius Joanito et al., Nature Genetics (2022)	10X 3'-seq	syn26844071 (SG1)	14
	Ignasius Joanito et al., Nature Genetics (2022)	10X 3'-seq	syn26844071 (SG2)	12
HCC	*Bojan Losic et al., Nature Communications (2019)*	10X 3'-seq	GSE112271	2
	Lichun Ma et al., Cancer Cell (2019)	10X 3'-seq	GSE125449	15
	Yiming Lu et al., Nature Communications (2022)	10X 3'-seq	GSE149614	10
	Ankur Sharma et al., Cell (2020)	10X 3'-seq	GSE156625	8
	Daniel Wai-Hung Ho et al., Nature Communications (2021)	10X 3'-seq	SRP318499	8
HNSC	*Sidharth V. Puram et al., Cell (2017)*	Smart-seq2	GSE103322	10
LC	*Ashley Maynard et atl., Cell (2020)*	Smart-seq2	PRJNA591860	24
	Nayoung Kim et al., Nature Communications (2020)	10X 3'-seq	GSE131907	21
	Junbin Qian et al. Cell Research (2020)	10X 3'-seq	http://blueprint.lambrechtslab.org	8
MEL	*Livnat Jerby-Arnon et al., Cell (2018)*	Smart-seq2	GSE115978	23
OVC	*Junbin Qian et al. Cell Research (2020)*	10X 3'-seq	http://blueprint.lambrechtslab.org	4
	Susan Olalekan et al., Cell Reports (2021)	Drop-seq	GSE147082	6
SKSC	*Andrew L. Ji et al., Cell (2020)*	10X 3'-seq	GSE144236	7

Sample #	Malignant cell #	UVG clustering		HVG clutsering	
		Avg. % of PT cluster	Avg. % of sample cluster	Avg. % of PT cluster	Avg. % of sample cluster
=	10836	26.06%	=	93.85%	=
=	11878	31.15%	=	79.05%	=
=	1775	10.01%	=	79.56%	=
=	16235	25.18%	=	72.10%	=
=	17334	6.35%	=	57.21%	=
18	6667	36.30%	14.76%	76.61%	61.57%
14	10214	57.40%	11.52%	94.81%	68.28%
54<br>25	18654	6.58%	0.00%	53.72%	22.45%
7	26753	64.15%	9.00%	86.07%	49.33%
=	1942	10.01%	=	84.89%	=
12	15971	30.62%	25.53%	89.40%	83.87%
22	12377	74.52%	25.38%	99.74%	55.77%
=	17455	30.13%	=	69.95%	=
=	1403	0.00%	=	33.42%	=
30	3035	9.61%	9.61%	54.40%	54.40%
=	24783	23.41%	=	88.50%	=
20	11906	21.29%	1.53%	99.31%	68.23%
=	1968	5.63%	=	59.16%	=
5	4052	33.01%	18.07%	100.00%	97.67%
=	3288	26.08%	=	88.95%	=
=	4883	30.10%	=	48.39%	=
	**Average:**	26.55%	16.17%	76.62%	67.46%
