# Supplementary material for: Clustering malignant cell states using universally variable genes
Source: Brief Bioinform. 2023 Dec 11;25(1):bbad460. doi: 10.1093/bib/bbad460 (PMC10783859; doi:10.1093/bib/bbad460)
Supplement: Suppmentary_Table_3_bbad460 [file suppmentary_table_3_bbad460.pdf]

Symbol	# of datasets
ATXN7L2	6
CCNB1	6
CENPF	6
DDIT3	6
ADNP-AS1	5
ALOX5AP	5
ARHGAP15	5
C20orf144	5
CARD8-AS1	5
CASS4	5
CCL5	5
CCNB2	5
CCR7	5
CD247	5
CD28	5
CD3D	5
CD40LG	5
CDK1	5
DDX39B-AS1	5
DEPDC1	5
DPEP2	5
EFCAB6	5
FAM151B	5
FGL2	5
GCSAM	5
GIMAP6	5
HMGB2	5
ICOS	5
IL16	5
IL32	5
ITK	5
KLHL17	5
KLHL31	5
KLHL6	5
LCP2	5
LILRB1	5
PAN3-AS1	5
PATL2	5
PIH1D2	5
PLEKHM3	5
PLK1	5
PPT2-EGFL8	5
PTPRC	5
PTTG1	5
RASAL2-AS1	5
SASH3	5
SCARF1	5
SEMA6A-AS1	5

SLA	5
SMIM11B	5
SPN	5
TAGAP	5
TCP11L2	5
TROAP	5
UBE2C	5
ZFAT	5
ZNF112	5
ZNF280C	5
ZNF674	5
ZSCAN20	5
ABI3	4
AP001437.1	4
ARHGAP9	4
ASPM	4
BBS12	4
BCDIN3D-AS1	4
BOLA2	4
BTK	4
C16orf95	4
C19orf38	4
C1orf162	4
C6orf163	4
C7orf25	4
C9orf147	4
CAPN11	4
CBLN3	4
CCL4	4
CD160	4
CD2	4
CD226	4
CD300LF	4
CD33	4
CD37	4
CD3E	4
CD48	4
CD5	4
CD53	4
CD7	4
CD84	4
CD86	4
CD96	4
CDAN1	4
CDC20	4
CDCA3	4
CDCA7	4
CDCA8	4
CDKL3	4

CDKN3	4
CDNF	4
CFAP54	4
CHRNA10	4
CKAP2	4
CLEC4A	4
CLECL1	4
CORT	4
CRTAM	4
CTBP1-AS	4
CXorf21	4
CYTH4	4
DDX47	4
DNAJC28	4
DNAJC5B	4
DNASE1L2	4
DSCR9	4
EFCAB13	4
EFCAB5	4
EFCAB7	4
EVI2B	4
FALEC	4
FAM78A	4
FCGR1A	4
FGR	4
FLI1	4
FRS3	4
GARNL3	4
GIMAP4	4
GIMAP5	4
GNG12-AS1	4
GPR171	4
GPR19	4
GTF3C2-AS1	4
GZMH	4
HAVCR2	4
HMMR	4
IFI30	4
IKZF1	4
IL21R	4
IL2RA	4
ITGA4	4
KCNE1	4
KCTD21-AS1	4
KDM4A-AS1	4
KDM4D	4
KIF14	4
KIF24	4
KIF9-AS1	4

KLHDC1	4
KLLN	4
KLRB1	4
KLRC1	4
KPNA2	4
LAIR1	4
LILRB2	4
LILRB4	4
LINC00235	4
LINC00266-1	4
LINC00426	4
LINC00630	4
LINC00996	4
LINC01094	4
LINC01572	4
LRRC70	4
LY86	4
MCMDC2	4
MIAT	4
MIR155HG	4
MIR181A2HG	4
MNDA	4
MT2A	4
MYLK4	4
NDOR1	4
NIPSNAP3B	4
NLRC3	4
NLRP3	4
NPR2	4
OASL	4
OSGEPL1-AS1	4
P2RY14	4
PARP15	4
PDCD1LG2	4
PIK3R5	4
PILRA	4
PINK1-AS	4
PKD2L2	4
PLA2G4E-AS1	4
PLVAP	4
PRAM1	4
PRKAR2A-AS1	4
PROB1	4
PTGDR	4
PTPN22	4
QPCTL	4
RAB33A	4
RAD21-AS1	4
RAG1	4

RASAL3	4
RASGRP4	4
RBM44	4
RBPMS-AS1	4
RGPD8	4
RNASE6	4
RNF122	4
RORA-AS1	4
RPA4	4
RUSC2	4
SAXO1	4
SDCBP2-AS1	4
SH2D1A	4
SH2D1B	4
SIGLEC10	4
SIRPG	4
SIT1	4
SLA2	4
SLAMF1	4
SLAMF6	4
SLC25A34	4
SLX4	4
SMC5-AS1	4
SNX15	4
SPAG8	4
SPI1	4
SRCAP	4
ST8SIA4	4
STAM-AS1	4
STAT4	4
STAU2-AS1	4
TAF1A-AS1	4
TAPT1-AS1	4
TAS2R4	4
TBX21	4
TFEC	4
TIGD7	4
TNF	4
TOP2A	4
TRAT1	4
TRBC2	4
TRG-AS1	4
TRPV1	4
TTC25	4
TUBA1B	4
TUBB4B	4
UBASH3A	4
UBE2F-SCLY	4
VN1R1	4

WAS	4
WDR19	4
WEE2-AS1	4
WNT2B	4
ZACN	4
ZBTB3	4
ZBTB39	4
ZCWPW2	4
ZGRF1	4
ZKSCAN2	4
ZMIZ1-AS1	4
ZMYND10	4
ZNF100	4
ZNF154	4
ZNF155	4
ZNF221	4
ZNF230	4
ZNF235	4
ZNF449	4
ZNF527	4
ZNF564	4
ZNF607	4
ZNF619	4
ZNF624	4
ZNF775	4
ZNF805	4
ZNF845	4
ZSCAN22	4
ZXDA	4
ABCD2	3
AC007128.1	3
AC008522.1	3
AC012314.8	3
AC083843.2	3
AC093382.1	3
AC107081.5	3
AC133644.2	3
ACER2	3
ACVR2B-AS1	3
ADAM20	3
ADGRA2	3
ADGRG3	3
ADHFE1	3
AGBL2	3
AGBL3	3
AIF1	3
AIRN	3
AL358852.1	3
ALG10	3

ALG10B	3
ALG9	3
ALKBH8	3
ANGPT2	3
ANKDD1B	3
ANKRD23	3
ANKRD31	3
AOC2	3
AP000442.1	3
AP001412.1	3
APBB1IP	3
AREG	3
ARF4-AS1	3
ARHGAP19	3
ARHGAP25	3
ARHGAP31-AS1	3
ARHGEF15	3
ARMC2	3
ARMCX4	3
ARRDC3-AS1	3
ASAH2	3
ASB16	3
ASMTL-AS1	3
ATF3	3
AXDND1	3
B4GALT1-AS1	3
BACH1-AS1	3
BACH1-IT2	3
BBS10	3
BGLAP	3
BIRC5	3
BRCA2	3
BTBD19	3
BUB1	3
C11orf21	3
C11orf65	3
C11orf72	3
C11orf91	3
C12orf50	3
C12orf60	3
C15orf48	3
C1orf54	3
C1RL-AS1	3
C2-AS1	3
C2orf42	3
C3AR1	3
C5AR1	3
C8orf37	3
C9orf43	3

CALCRL	3
CAPN3	3
CAPS2	3
CARNS1	3
CCDC138	3
CCDC17	3
CCDC183-AS1	3
CCDC30	3
CCDC62	3
CCDC7	3
CCDC73	3
CCDC81	3
CCNT2-AS1	3
CCR1	3
CCR4	3
CCR5	3
CD180	3
CD200R1	3
CD274	3
CD300A	3
CD3G	3
CD4	3
CD6	3
CD69	3
CD72	3
CD80	3
CD8A	3
CD93	3
CDCA2	3
CDRT4	3
CEBPB-AS1	3
CENPA	3
CENPE	3
CEP192	3
CEP55	3
CFAP43	3
CFAP69	3
CFB	3
CHKB	3
CHST11	3
CIT	3
CKAP2L	3
CKS2	3
CLEC4E	3
CMC4	3
CMKLR1	3
COLQ	3
CPT1B	3
CRYGS	3

CSF2RB	3
CSF3R	3
CSNK2A3	3
CTB-39G8.2	3
CTD-2213F21.2	3
CTH	3
CTLA4	3
CTNS	3
CTRL	3
CTSK	3
CXCL2	3
CXCL8	3
CXCR5	3
CXCR6	3
CYBB	3
CYP51A1-AS1	3
CYSLTR2	3
CYTIP	3
DACT3	3
DDX11-AS1	3
DDX60	3
DEPDC4	3
DFFB	3
DLEU7	3
DNAH7	3
DNAJB1	3
DNAJB13	3
DNM1P35	3
DOCK2	3
DOCK8	3
DOK2	3
DUSP2	3
E2F7	3
ECT2L	3
EFHB	3
EGFL8	3
EGR2	3
EGR4	3
ELF3	3
ENO3	3
ENO4	3
ENTPD1	3
ERICD	3
ERICH6	3
ERICH6-AS1	3
ERMARD	3
ESPL1	3
EVC2	3
EVI2A	3

EYS	3
FAM102B	3
FAM122C	3
FAM182B	3
FAM209A	3
FANCC	3
FASLG	3
FBXO10	3
FBXO36	3
FBXO43	3
FCAR	3
FCGR2B	3
FCGR3B	3
FERMT3	3
FITM1	3
FOCAD-AS1	3
FOS	3
FOSL2	3
FOXD4L1	3
FOXP3	3
FRS2	3
FSCN3	3
FUT8-AS1	3
FYB	3
GAB3	3
GADD45B	3
GAPT	3
GAS5-AS1	3
GEN1	3
GHRLOS	3
GIMAP1	3
GIMAP7	3
GLB1L	3
GNG2	3
GNGT2	3
GNRH1	3
GOLGA6L9	3
GPR135	3
GPR183	3
GPR65	3
GRAP	3
GRAP2	3
GRASP	3
GTF2H2C	3
GTF2H4	3
GYS1	3
GZMA	3
GZMK	3
GZMM	3

HCG14	3
HCG20	3
HCG25	3
HCLS1	3
HEXA-AS1	3
HIGD1B	3
HIGD2B	3
HIPK1-AS1	3
HK3	3
HMGN2	3
HMGN3-AS1	3
HSD17B13	3
HSPA1A	3
HSPA1B	3
IDI2-AS1	3
IFFO1	3
IFI6	3
IFIT2	3
IFNG	3
IGSF6	3
IKZF4	3
IL10RA	3
IL18R1	3
IL18RAP	3
IL2RB	3
IL5	3
IL7R	3
INCA1	3
INSIG1	3
INTS9	3
IPO9-AS1	3
IRF1	3
IRF4	3
ISG15	3
ITGA8	3
ITGAX	3
ITGB7	3
JADRR	3
JAK2	3
JAML	3
KCNA3	3
KCNAB1	3
KCNAB3	3
KCNJ2-AS1	3
KCNMB1	3
KCTD21	3
KDM8	3
KIF18A	3
KIF20A	3

KIF20B	3
KIF23	3
KIFC1	3
KLRD1	3
KLRG1	3
KRT17	3
KY	3
LAG3	3
LAT2	3
LCA5	3
LCA5L	3
LCMT1-AS1	3
LCN2	3
LEKR1	3
LGALS1	3
LILRA1	3
LILRA2	3
LILRA5	3
LIN9	3
LINC00115	3
LINC00449	3
LINC00471	3
LINC00861	3
LINC00887	3
LINC00891	3
LINC00921	3
LINC01063	3
LINC01126	3
LINC01136	3
LINC01270	3
LINC01273	3
LINC01277	3
LINC01311	3
LINC01353	3
LINC01460	3
LINC01480	3
LINC01515	3
LMCD1-AS1	3
LOH12CR2	3
LRGUK	3
LRMP	3
LRP2BP	3
LRRC37A	3
LRRC37A2	3
LRRC46	3
LRRC56	3
LRRD1	3
LRSAM1	3
LST1	3

LTB	3
LY9	3
MAD2L1	3
MAGI2	3
MAMDC4	3
MAMSTR	3
MAS1	3
MATK	3
MATN1-AS1	3
MATR3	3
MCF2L2	3
MCM10	3
MCM3AP-AS1	3
MED14OS	3
MEFV	3
MIP	3
MIR34AHG	3
MIS18BP1	3
MKI67	3
MMP25	3
MMRN2	3
MORF4L2-AS1	3
MPEG1	3
MRPL53	3
MYO1F	3
MYT1L	3
NAPA-AS1	3
NAPSA	3
NCAPD2	3
NCBP2-AS1	3
NCDN	3
NCF2	3
NCKAP1L	3
NCR3	3
NDUFC2-KCTD14	3
NEK2	3
NEMP1	3
NHLH1	3
NHLRC4	3
NKG7	3
NLRC4	3
NPIPB11	3
NPIPB6	3
NR4A3	3
NRIP2	3
NRROS	3
NRTN	3
NUSAP1	3
OSCAR	3

OSM	3
OXCT2	3
P2RX5-TAX1BP3	3
P2RY10	3
P2RY13	3
PARD3-AS1	3
PARVG	3
PAXBP1-AS1	3
PCDHGA2	3
PCED1B	3
PDCD1	3
PDE1B	3
PDSS1	3
PFKFB1	3
PHLDB3	3
PHLPP1	3
PIK3CD	3
PIWIL2	3
PKMYT1	3
PLEK	3
PLK4	3
PPP1R16B	3
PRR11	3
PRR34	3
PSTPIP1	3
PTBP2	3
PTCH2	3
PTGIR	3
PTPN7	3
PYHIN1	3
RAB40AL	3
RACGAP1	3
RAD9B	3
RANBP6	3
RAPSN	3
RASL11B	3
REC8	3
RGS1	3
RHD	3
RHEBL1	3
RHOH	3
RIBC1	3
RNASEK-C17orf49	3
RNF144A	3
RNF31	3
RP11-16C1.1	3
RP11-244M2.1	3
RP11-268J15.5	3
RP11-580I16.2	3

RP5-1021I20.1	3
RPL17-C18orf32	3
RRM2	3
RSPH4A	3
RSPH9	3
RTCA-AS1	3
SAMD3	3
SAMSN1	3
SAPCD1-AS1	3
SEC24B-AS1	3
SEC31B	3
SELL	3
SELPLG	3
SERPINB9P1	3
SERPINE1	3
SGMS1-AS1	3
SH2D6	3
SH3BP5-AS1	3
SIGLEC9	3
SIRT4	3
SLAMF8	3
SLC12A6	3
SLC19A2	3
SLC22A1	3
SLC24A1	3
SLC26A4	3
SLC2A4	3
SLC4A9	3
SMIM11A	3
SNAI3-AS1	3
SPATA17	3
SPRED3	3
SPRY3	3
SRRM5	3
STAB1	3
STAC3	3
STK36	3
STMN1	3
STX18-AS1	3
STX1B	3
SUMO4	3
SYCP3	3
SYT11	3
SZT2-AS1	3
TANGO6	3
TAS2R14	3
TAS2R31	3
TBC1D10C	3
TBX6	3

TCP11L1	3
TCTN2	3
TEN1-CDK3	3
TESPA1	3
TEX14	3
TEX29	3
THAP9	3
THEMIS	3
TIE1	3
TIGD1	3
TIGIT	3
TLR7	3
TM6SF1	3
TMCO6	3
TMEM147-AS1	3
TMEM17	3
TMEM182	3
TMEM236	3
TMEM249	3
TMEM81	3
TMEM9B-AS1	3
TMIGD2	3
TNFAIP8L2	3
TNFRSF4	3
TNFSF13B	3
TNFSF8	3
TPX2	3
TRAC	3
TRAF1	3
TRAF3IP3	3
TRDC	3
TRIM21	3
TRIM35	3
TRIM62	3
TSNAX-DISC1	3
TSNAXIP1	3
TSSK6	3
TTC26	3
TTLL11	3
TVP23C	3
TYROBP	3
TYW1B	3
UBAP1L	3
UBE2E1-AS1	3
UBR5-AS1	3
UFSP1	3
USHBP1	3
USP20	3
VWA8-AS1	3

WDR47	3
WDR97	3
ZBED6CL	3
ZBTB32	3
ZBTB34	3
ZBTB49	3
ZC3H12D	3
ZC3HAV1	3
ZC3HC1	3
ZEB1	3
ZEB2	3
ZFP28	3
ZFP41	3
ZFP69	3
ZFP69B	3
ZFYVE1	3
ZKSCAN3	3
ZKSCAN5	3
ZMYM1	3
ZMYM6	3
ZNF141	3
ZNF169	3
ZNF17	3
ZNF180	3
ZNF182	3
ZNF19	3
ZNF2	3
ZNF222	3
ZNF225	3
ZNF23	3
ZNF25	3
ZNF252P-AS1	3
ZNF304	3
ZNF324B	3
ZNF329	3
ZNF341-AS1	3
ZNF343	3
ZNF354B	3
ZNF366	3
ZNF367	3
ZNF384	3
ZNF396	3
ZNF41	3
ZNF417	3
ZNF425	3
ZNF432	3
ZNF436	3
ZNF436-AS1	3
ZNF441	3

ZNF443	3
ZNF461	3
ZNF484	3
ZNF510	3
ZNF546	3
ZNF547	3
ZNF573	3
ZNF575	3
ZNF585A	3
ZNF585B	3
ZNF596	3
ZNF606	3
ZNF616	3
ZNF678	3
ZNF683	3
ZNF695	3
ZNF700	3
ZNF709	3
ZNF718	3
ZNF75D	3
ZNF763	3
ZNF772	3
ZNF776	3
ZNF782	3
ZNF783	3
ZNF79	3
ZNF8	3
ZNF841	3
ZNF852	3
ZRANB3	3
ZSCAN12	3
A2M	2
AARD	2
AARS2	2
AATBC	2
ABALON	2
ABCA2	2
ABCB5	2
ABCC11	2
AC002306.1	2
AC004490.1	2
AC004549.6	2
AC005363.11	2
AC005480.1	2
AC007391.2	2
AC007405.4	2
AC007620.3	2
AC007950.2	2
AC009065.4	2

AC012531.25	2
AC017002.1	2
AC019186.1	2
AC022182.3	2
AC025335.1	2
AC026202.3	2
AC062029.1	2
AC068196.1	2
AC068831.6	2
AC073657.1	2
AC079807.2	2
AC090498.1	2
AC092580.4	2
AC097381.1	2
AC098820.4	2
AC104653.1	2
AC105339.1	2
AC113404.1	2
AC114271.2	2
AC138035.2	2
AC139887.4	2
AC144449.1	2
AC145212.2	2
ACAP1	2
ACKR1	2
ACRV1	2
ACSM3	2
ACSM5	2
ACTA1	2
ACTL6A	2
ACTN2	2
ACTR8	2
ACVR1C	2
ACVRL1	2
ADAM20P1	2
ADAM32	2
ADAMTS13	2
ADAMTS4	2
ADAMTSL4	2
ADAMTSL5	2
ADARB1	2
ADAT2	2
ADCY10	2
ADCY4	2
ADGRD2	2
ADGRF3	2
ADGRF5	2
ADM5	2
ADORA3	2

ADPGK-AS1	2
ADPRM	2
AF131216.1	2
AFAP1L1	2
AGER	2
AGPAT5	2
AHSP	2
AK7	2
AKAP14	2
AKNA	2
AKNAD1	2
AL023806.1	2
ALDH1L2	2
ALMS1	2
ALPK1	2
AMER1	2
AMY2B	2
ANKHD1-EIF4EBP3	2
ANKK1	2
ANKLE2	2
ANKMY1	2
ANKRD24	2
ANKRD27	2
ANKRD34A	2
ANKRD37	2
ANKRD44	2
ANKUB1	2
ANLN	2
AOAH	2
AP000253.1	2
AP000692.10	2
AP000708.1	2
AP000866.1	2
AP006621.6	2
AP1AR	2
AP4B1	2
AP4B1-AS1	2
APBB3	2
APC2	2
APOA1	2
APOBEC3A	2
APOLD1	2
APOM	2
AQP11	2
AQP4	2
AQP4-AS1	2
ARAP1	2
ARAP2	2
ARAP3	2

ARHGAP11A	2
ARHGAP29	2
ARHGAP32	2
ARHGAP33	2
ARHGAP42	2
ARHGEF39	2
ARHGEF6	2
ARID5B	2
ARL4C	2
ARL6	2
ARL6IP1	2
ARMC12	2
ARNTL	2
ARSD-AS1	2
ART4	2
ASB2	2
ASB9	2
ASIP	2
ASPRV1	2
ASTE1	2
ATAD2B	2
ATAD3B	2
ATCAY	2
ATP10D	2
ATP9B	2
AURKA	2
AURKC	2
AVIL	2
BACE1-AS	2
BACH2	2
BATF	2
BBOF1	2
BBS1	2
BBS9	2
BCO2	2
BDNF-AS	2
BHLHB9	2
BHLHE40-AS1	2
BHMT2	2
BIN2	2
BLM	2
BMF	2
BNIPL	2
BOLA2B	2
BRAF	2
BRAP	2
BRIP1	2
BSN	2
BTG2	2

BTLA	2
BUB1B	2
C10orf113	2
C10orf12	2
C10orf25	2
C11orf45	2
C11orf52	2
C11orf94	2
C12orf43	2
C12orf66	2
C14orf178	2
C16orf54	2
C16orf71	2
C16orf86	2
C17orf100	2
C17orf50	2
C17orf77	2
C17orf99	2
C18orf54	2
C19orf44	2
C1GALT1C1L	2
C1orf194	2
C1orf220	2
C1orf74	2
C1QB	2
C21orf62-AS1	2
C2orf44	2
C2orf50	2
C3orf35	2
C4orf47	2
C5orf34	2
C5orf56	2
C6orf47-AS1	2
C7orf43	2
C8orf37-AS1	2
C8orf44	2
C8orf44-SGK3	2
C9orf153	2
C9orf163	2
C9orf64	2
C9orf72	2
CA5B	2
CACNA2D3	2
CACNA2D4	2
CACNB1	2
CACNB4	2
CAHM	2
CALCOCO1	2
CAMK4	2

CAMSAP1	2
CAP2	2
CAPN14	2
CARF	2
CASC1	2
CASC5	2
CASC8	2
CASK	2
CASP5	2
CATSPER3	2
CBARP	2
CBFA2T2	2
CCAR2	2
CCBL1	2
CCDC102B	2
CCDC114	2
CCDC116	2
CCDC120	2
CCDC134	2
CCDC141	2
CCDC150	2
CCDC18	2
CCDC185	2
CCDC38	2
CCDC39	2
CCDC65	2
CCDC69	2
CCER2	2
CCL14	2
CCL20	2
CCL23	2
CCL3	2
CCL3L3	2
CCL4L2	2
CCM2L	2
CCNA2	2
CCNE2	2
CCNJ	2
CCR2	2
CCR6	2
CCR8	2
CCT2	2
CD101	2
CD1C	2
CD207	2
CD244	2
CD27	2
CD300C	2
CD300E	2

CD34	2
CD52	2
CD70	2
CDC14A	2
CDC25A	2
CDC25C	2
CDC7	2
CDH24	2
CDK2	2
CDK20	2
CDK5R1	2
CDKL4	2
CDO1	2
CEACAM19	2
CECR1	2
CELF2	2
CELSR2	2
CENPI	2
CENPO	2
CENPQ	2
CENPW	2
CEP120	2
CEP128	2
CEP295NL	2
CEP72	2
CEP85	2
CERS1	2
CERS5	2
CFAP157	2
CFAP161	2
CFAP45	2
CFAP53	2
CFAP57	2
CFAP61	2
CFAP74	2
CGB7	2
CHCHD3	2
CHEK2	2
CIRBP-AS1	2
CKS1B	2
CLASP1	2
CLDN11	2
CLDN16	2
CLEC10A	2
CLEC12A	2
CLEC1A	2
CLEC2D	2
CLEC7A	2
CLHC1	2

CLIC3	2
CLIC5	2
CLPSL2	2
CLSPN	2
CLTCL1	2
CNIH4	2
CNPY4	2
CNR2	2
CNRIP1	2
CNTRL	2
COL8A2	2
CORO1A	2
CORO6	2
CPEB2	2
CPNE5	2
CPNE8	2
CPNE9	2
CPO	2
CPSF4L	2
CRTC3-AS1	2
CRX	2
CRYBB1	2
CRYBB3	2
CRYM-AS1	2
CRYZ	2
CSE1L-AS1	2
CSF1R	2
CST7	2
CTA-392E5.1	2
CTA-397H3.3	2
CTB-161M19.4	2
CTB-25B13.9	2
CTC-276P9.4	2
CTC-428H11.2	2
CTC-453G23.8	2
CTC-487M23.8	2
CTC-498J12.3	2
CTC1	2
CTD-2036P10.3	2
CTD-2231E14.8	2
CTD-2270P14.5	2
CTD-2369P2.5	2
CTD-2376I4.2	2
CTD-2410N18.5	2
CTD-2547L16.1	2
CTD-2630F21.1	2
CTD-3131K8.2	2
CTD-3193K9.4	2
CTGF	2

CUBN	2
CWF19L1	2
CXCL13	2
CXCR3	2
CXorf65	2
CYB561D1	2
CYP19A1	2
CYP1B1-AS1	2
CYP4F22	2
CYR61	2
CYSLTR1	2
DACT1	2
DACT3-AS1	2
DBF4B	2
DCAF4L1	2
DCDC2B	2
DCST2	2
DCUN1D3	2
DDN	2
DDX20	2
DDX31	2
DDX58	2
DENND1C	2
DENND5B-AS1	2
DENND6A	2
DEPDC1-AS1	2
DEPDC1B	2
DGCR5	2
DGKQ	2
DHH	2
DHX34	2
DHX35	2
DHX58	2
DICER1-AS1	2
DLC1	2
DLEC1	2
DLG1-AS1	2
DLG2	2
DLGAP1-AS2	2
DLGAP4-AS1	2
DLL4	2
DMGDH	2
DNA2	2
DNAH1	2
DNAH10OS	2
DNAH6	2
DNAJB14	2
DNAJB9	2
DNAJC25-GNG10	2

DNAJC27	2
DNAJC6	2
DNAJC9-AS1	2
DNHD1	2
DOCK5	2
DOK3	2
DPH6-AS1	2
DPT	2
DRD4	2
DRICH1	2
DTHD1	2
DTL	2
DUSP1	2
DUSP19	2
DYSF	2
DZANK1	2
DZIP1	2
EBI3	2
ECE2	2
ECM2	2
EDN1	2
EDRF1-AS1	2
EFHC2	2
EGOT	2
EGR1	2
EIF3CL	2
ELFN2	2
ELMO1	2
ELN	2
ELP3	2
EME1	2
EMP3	2
EMX2OS	2
ENTPD4	2
EPHB1	2
EPN1	2
EPT1	2
ERCC6L	2
EREG	2
ERI1	2
ERMAP	2
ERVW-1	2
ESR1	2
ETAA1	2
ETS1	2
ETS2	2
EVPL	2
EXO1	2
EXO5	2

EXOC6B	2
EXTL3-AS1	2
F8	2
FABP1	2
FABP5	2
FAM105A	2
FAM110D	2
FAM124B	2
FAM131C	2
FAM13A-AS1	2
FAM13C	2
FAM149B1	2
FAM157C	2
FAM159A	2
FAM166A	2
FAM166B	2
FAM183A	2
FAM184B	2
FAM185A	2
FAM214B	2
FAM227B	2
FAM234B	2
FAM53A	2
FAM64A	2
FAM66B	2
FAM72B	2
FAM73A	2
FAM73B	2
FAM83D	2
FAM85B	2
FAM86B2	2
FAM87B	2
FANCL	2
FAS	2
FBN1	2
FBXL13	2
FBXL14	2
FBXO16	2
FBXO30	2
FBXO39	2
FBXO5	2
FCER1G	2
FCGR3A	2
FCRL3	2
FCRLA	2
FDXACB1	2
FER	2
FGD2	2
FGD3	2

FKTN	2
FLG2	2
FLT4	2
FMO1	2
FMR1	2
FOLR2	2
FOXD4	2
FOXM1	2
FOXN3-AS1	2
FOXO6	2
FPGS	2
FPR1	2
FRMPD1	2
FSCN2	2
FSD2	2
FUT7	2
FXYD7	2
FYCO1	2
GABBR2	2
GABPB2	2
GAL3ST4	2
GAS2L3	2
GBP5	2
GDF15	2
GDF9	2
GDPD1	2
GFI1	2
GFPT2	2
GHET1	2
GHRL	2
GIMAP8	2
GINS3	2
GIPR	2
GJA4	2
GLIPR1L2	2
GLMN	2
GLO1	2
GLOD5	2
GLUD2	2
GMFG	2
GMIP	2
GNG3	2
GOLGA6L4	2
GOLGA8N	2
GPBAR1	2
GPR1	2
GPR132	2
GPR137C	2
GPR18	2

GPR34	2
GPR35	2
GPR61	2
GPR75	2
GPR82	2
GPRASP2	2
GPRIN1	2
GPSM2	2
GPT	2
GRIN2C	2
GRM6	2
GSG2	2
GSN-AS1	2
GSTA1	2
GSTM2	2
GTDC1	2
GTF2B	2
GTSE1	2
GZMB	2
H1FX-AS1	2
H2AFZ	2
HAL	2
HAND2-AS1	2
HARS	2
HAS3	2
HAT1	2
HAUS8	2
HCG15	2
HCG16	2
HCG17	2
HCST	2
HEATR5B	2
HERC6	2
HIC1	2
HIST1H1D	2
HIST1H4C	2
HIST2H4B	2
HJURP	2
HLA-DOB	2
HM13-AS1	2
HMGB1	2
HMX2	2
HNRNPA1	2
HPCA	2
HPD	2
HPGDS	2
HRC	2
HRH4	2
HSF2BP	2

HSPA13	2
HSPA1L	2
HSPA5	2
HSPA6	2
HSPA8	2
HSPB2	2
HSPB9	2
HTR2B	2
HUS1B	2
HVCN1	2
HYI-AS1	2
HYPK	2
ICAM5	2
ID2-AS1	2
IDH1-AS1	2
IER2	2
IER3	2
IFIH1	2
IFIT1	2
IFIT3	2
IFT140	2
IFT74-AS1	2
IGFBP3	2
IGFL4	2
IGFLR1	2
IGHV3-23	2
IL10	2
IL12A	2
IL12RB1	2
IL1RL1	2
IL21R-AS1	2
IL23R	2
IL2RG	2
IL34	2
IL5RA	2
INCENP	2
INHA	2
INHBA	2
INMT	2
INPP5D	2
INPP5E	2
INTS6-AS1	2
IPCEF1	2
IPMK	2
IQCH	2
IQCH-AS1	2
IQGAP3	2
IQUB	2
IRAK1BP1	2

ITGA2	2
ITGAL	2
ITGB8	2
ITPK1-AS1	2
ITPKB-AS1	2
ITPRIP	2
ITPRIPL1	2
IZUMO1	2
JAK3	2
JAKMIP2	2
JAM2	2
JMJD1C-AS1	2
JMJD7-PLA2G4B	2
JUN	2
JUNB	2
KCNA7	2
KCND1	2
KCNIP2	2
KCNIP2-AS1	2
KCNJ14	2
KCNJ5	2
KCNN3	2
KCTD18	2
KCTD7	2
KDM4C	2
KIAA0556	2
KIAA0753	2
KIAA0825	2
KIAA1324	2
KIAA2012	2
KIF11	2
KIF15	2
KIF17	2
KIF21B	2
KIR2DL3	2
KIR2DL4	2
KIR3DX1	2
KLHDC7A	2
KLHDC7B	2
KLHL10	2
KLHL11	2
KLHL32	2
KNTC1	2
KRBA2	2
KRT18	2
KRT80	2
KRT86	2
KRTAP5-10	2
KSR1	2

KTN1-AS1	2
LA16c-313D11.9	2
LACC1	2
LACTB2-AS1	2
LAMA2	2
LAMP3	2
LANCL1	2
LANCL3	2
LARP4	2
LARP7	2
LARS2	2
LAYN	2
LBR	2
LBX2-AS1	2
LCAT	2
LCK	2
LCMT1-AS2	2
LCN12	2
LCN6	2
LDB3	2
LENG8-AS1	2
LEXM	2
LGALS8-AS1	2
LIG4	2
LILRA4	2
LILRB3	2
LIMD1-AS1	2
LIMD2	2
LIME1	2
LINC00092	2
LINC00158	2
LINC00184	2
LINC00243	2
LINC00265	2
LINC00271	2
LINC00294	2
LINC00299	2
LINC00310	2
LINC00313	2
LINC00324	2
LINC00337	2
LINC00339	2
LINC00525	2
LINC00571	2
LINC00632	2
LINC00636	2
LINC00637	2
LINC00852	2
LINC00862	2

LINC00870	2
LINC00877	2
LINC00881	2
LINC00884	2
LINC00885	2
LINC00894	2
LINC00907	2
LINC00910	2
LINC00924	2
LINC00987	2
LINC00997	2
LINC01004	2
LINC01068	2
LINC01088	2
LINC01119	2
LINC01124	2
LINC01135	2
LINC01144	2
LINC01160	2
LINC01170	2
LINC01176	2
LINC01191	2
LINC01204	2
LINC01209	2
LINC01215	2
LINC01271	2
LINC01291	2
LINC01337	2
LINC01342	2
LINC01355	2
LINC01358	2
LINC01376	2
LINC01424	2
LINC01483	2
LINC01503	2
LINC01547	2
LINC01551	2
LINC01562	2
LIPC	2
LIPT2	2
LL09NC01-251B2.3	2
LMAN2L	2
LMNB1	2
LMO2	2
LNP1	2
LOXL3	2
LPAR4	2
LPXN	2
LRIT3	2

LRP4-AS1	2
LRRC17	2
LRRC25	2
LRRC27	2
LRRC29	2
LRRC37A3	2
LRRC39	2
LRRC4	2
LRRC69	2
LRRC74B	2
LRRC8C	2
LRRIQ3	2
LRRN3	2
LRRTM2	2
LSINCT5	2
LSM11	2
LSMEM2	2
LTA	2
LTB4R2	2
LTF	2
LY6E	2
LY6G5B	2
LYG1	2
LYPD3	2
LYPD5	2
MAB21L3	2
MACROD2	2
MADCAM1	2
MAFA	2
MAFF	2
MAFTRR	2
MAGOH	2
MANBA	2
MAP1LC3B2	2
MAP3K14-AS1	2
MAP3K15	2
MAP3K2	2
MAP3K7CL	2
MAP4K1	2
MAPKBP1	2
MAST1	2
MASTL	2
MBD6	2
MBOAT1	2
MCM2	2
MCM7	2
MCM9	2
MCPH1-AS1	2
MDS2	2

ME1	2
MED22	2
MEF2B	2
MEF2C	2
MEI1	2
MEIG1	2
MEIOC	2
MELK	2
METTL14	2
METTL25	2
MFAP3	2
MFHAS1	2
MFNG	2
MGST1	2
MIATNB	2
MIR193BHG	2
MIR3142HG	2
MIR503HG	2
MIS18A-AS1	2
MKS1	2
MMAA	2
MMACHC	2
MMP24	2
MMP7	2
MOB3C	2
MORN3	2
MROH8	2
MS4A4A	2
MS4A6A	2
MSANTD1	2
MSH5	2
MSMO1	2
MSR1	2
MSS51	2
MT1HL1	2
MTCP1	2
MTFP1	2
MTRNR2L4	2
MTUS2	2
MX1	2
MYBL1	2
MYH11	2
MYH3	2
MYL9	2
MYO15A	2
MYO18A	2
MYO9A	2
MYOM1	2
MZF1-AS1	2

NAA40	2
NAALADL1	2
NAIF1	2
NANOG	2
NANP	2
NAT16	2
NATD1	2
NAV3	2
NBPF12	2
NBPF20	2
NBPF9	2
NCAPD3	2
NCAPG	2
NCF1	2
NCR1	2
NDC80	2
NDUFAF3	2
NDUFB2-AS1	2
NEIL3	2
NEK10	2
NEU3	2
NEURL4	2
NEXN-AS1	2
NFKB1	2
NFYC-AS1	2
NIM1K	2
NKPD1	2
NME1-NME2	2
NMNAT1	2
NMT2	2
NOMO2	2
NOP14-AS1	2
NOTCH4	2
NOX4	2
NPHP3-ACAD11	2
NPHS1	2
NPIPA1	2
NPIPA3	2
NPIPA5	2
NPIPB4	2
NPIPB8	2
NPL	2
NR4A2	2
NR6A1	2
NRL	2
NT5DC3	2
NT5M	2
NTN5	2
NUF2	2

NXPE3	2
OAS1	2
OAT	2
OCM	2
ODF2	2
OGFR-AS1	2
OGN	2
OLAH	2
OLMALINC	2
OLR1	2
OMD	2
OMG	2
ONECUT2	2
OR2A7	2
OR2B6	2
ORC1	2
ORM1	2
ORM2	2
OSGEPL1	2
OTOA	2
OTUD3	2
OVOL1-AS1	2
P2RX7	2
PACRG	2
PAK1IP1	2
PANK4	2
PAQR8	2
PARPBP	2
PBK	2
PCDH11X	2
PCDHB15	2
PCDHGA1	2
PCDHGA12	2
PCDHGA5	2
PCDHGA8	2
PCDHGB7	2
PCNA	2
PCOLCE-AS1	2
PDE1A	2
PDE6G	2
PDGFRL	2
PDK4	2
PDXP	2
PDZD2	2
PDZD3	2
PDZD7	2
PEX12	2
PFAS	2
PFN4	2

PGF	2
PHEX	2
PHLDB1	2
PI16	2
PIF1	2
PIGL	2
PIGR	2
PIK3R4	2
PINLYP	2
PIP	2
PIP5KL1	2
PITPNM2	2
PKD1L3	2
PKD2L1	2
PKN2	2
PLA2G2D	2
PLA2G4C	2
PLCB2	2
PLCD3	2
PLCG1-AS1	2
PLD6	2
PLEKHA4	2
PLEKHG2	2
PLEKHG5	2
PLGLB1	2
PLIN4	2
PLSCR2	2
PLXND1	2
PM20D1	2
PMEL	2
PMF1-BGLAP	2
PNPLA7	2
POC1A	2
POC1B	2
POLH	2
POLN	2
POLQ	2
POLR3B	2
POPDC2	2
POT1-AS1	2
POTEG	2
POU5F2	2
POU6F1	2
PPIP5K1	2
PPM1M	2
PPP1R14A	2
PPP1R26-AS1	2
PPP1R3G	2
PPP3R1	2

PPP5D1	2
PRC1	2
PRC1-AS1	2
PRDM10	2
PRDM15	2
PRDX2	2
PRELP	2
PREX2	2
PRIM1	2
PRKAB1	2
PRKCB	2
PRKCE	2
PRMT5-AS1	2
PRMT9	2
PRNCR1	2
PROCA1	2
PROK2	2
PRPF40B	2
PRPSAP2	2
PRR19	2
PRR29-AS1	2
PRR3	2
PRSS53	2
PRX	2
PSMD6-AS2	2
PSPN	2
PSRC1	2
PTAFR	2
PTCRA	2
PTGES2	2
PTGES3L-AARSD1	2
PTOV1-AS1	2
PTPN6	2
PTPRO	2
PVRIG	2
PYCARD	2
RAB37	2
RAB40A	2
RABL2A	2
RAD51	2
RAD52	2
RAD54B	2
RAI1	2
RAN	2
RAP2C-AS1	2
RAPGEF3	2
RASA4B	2
RAVER1	2
RBBP8	2

RBM14-RBM4	2
RBM26-AS1	2
RBM34	2
RBM48	2
RBM5-AS1	2
RBMS2	2
RBP5	2
RC3H2	2
RCBTB2	2
RCN3	2
RCSD1	2
RDM1	2
REEP4	2
REM2	2
RFC5	2
RFESD	2
RFX3-AS1	2
RGL1	2
RGL4	2
RGPD1	2
RGS9BP	2
RGSL1	2
RHBDD1	2
RHPN1-AS1	2
RIBC2	2
RILPL1	2
RIMS3	2
RLN1	2
RMDN2	2
RMRP	2
RN7SL832P	2
RNASE1	2
RNASE4	2
RNASEL	2
RNF103-CHMP3	2
RNF139-AS1	2
RNF180	2
RNF215	2
RNF222	2
RNF25	2
RNU12	2
RP1-234P15.4	2
RP1-278C19.8	2
RP11-1000B6.8	2
RP11-1007O24.3	2
RP11-109N23.4	2
RP11-10A14.4	2
RP11-120D5.1	2
RP11-1212A22.4	2

RP11-121C2.2	2
RP11-121M22.1	2
RP11-123K3.9	2
RP11-126K1.2	2
RP11-126K1.6	2
RP11-1348G14.4	2
RP11-134L10.1	2
RP11-135A24.4	2
RP11-152N13.5	2
RP11-158K1.3	2
RP11-160H22.3	2
RP11-178L8.7	2
RP11-182N22.8	2
RP11-206L10.9	2
RP11-216B9.6	2
RP11-243J16.7	2
RP11-264B17.4	2
RP11-267M23.4	2
RP11-277B15.3	2
RP11-299G20.2	2
RP11-304M2.5	2
RP11-305L7.7	2
RP11-307C12.13	2
RP11-312B8.1	2
RP11-317P15.4	2
RP11-321N4.5	2
RP11-325F22.2	2
RP11-327F22.1	2
RP11-341N2.1	2
RP11-348N5.7	2
RP11-348P10.2	2
RP11-34P13.7	2
RP11-356I2.4	2
RP11-35G9.5	2
RP11-378J18.9	2
RP11-386G11.10	2
RP11-396C23.4	2
RP11-421M1.8	2
RP11-429J17.7	2
RP11-432J22.2	2
RP11-443B20.1	2
RP11-465N4.4	2
RP11-46A10.5	2
RP11-486O12.2	2
RP11-48B3.5	2
RP11-510M2.2	2
RP11-517B11.4	2
RP11-532F6.4	2
RP11-539I5.1	2

RP11-53I6.3	2
RP11-544D21.2	2
RP11-545M17.1	2
RP11-559M23.1	2
RP11-576I22.2	2
RP11-589C21.5	2
RP11-626G11.5	2
RP11-646I6.5	2
RP11-649E7.5	2
RP11-650K20.3	2
RP11-687F6.5	2
RP11-692D12.1	2
RP11-697E2.12	2
RP11-697E2.6	2
RP11-6N17.2	2
RP11-722E23.2	2
RP11-725P16.2	2
RP11-74J13.8	2
RP11-762I7.5	2
RP11-77P6.2	2
RP11-796E2.4	2
RP11-849F2.7	2
RP11-84G21.1	2
RP11-87C12.5	2
RP11-977G19.11	2
RP11-983P16.4	2
RP11-98I9.4	2
RP11-996F15.2	2
RP2	2
RP3-329A5.8	2
RP4-536B24.4	2
RP4-564F22.5	2
RP4-669L17.10	2
RP4-758J18.13	2
RP5-1021I20.4	2
RP5-1057I20.4	2
RP5-1074L1.1	2
RP5-1074L1.4	2
RP5-1157M23.2	2
RP5-1172N10.2	2
RP5-1184F4.7	2
RP5-901A4.1	2
RP5-956O18.3	2
RP5-991G20.1	2
RP6-109B7.2	2
RPL17	2
RPL34-AS1	2
RPPH1	2
RRH	2

RRM1	2
RRS1-AS1	2
RSAD2	2
RSG1	2
RSPH10B	2
RTTN	2
RUFY4	2
RUNX3	2
RWDD2A	2
S100B	2
S1PR1	2
S1PR4	2
SAG	2
SALRNA2	2
SAMD9L	2
SAP130	2
SAP30L-AS1	2
SARNP	2
SCAANT1	2
SCHIP1	2
SCIMP	2
SCML4	2
SCN11A	2
SCN4B	2
SCOC-AS1	2
SCRG1	2
SCRN3	2
SDK2	2
SDR42E2	2
SDS	2
SEC62-AS1	2
SECTM1	2
SELE	2
SEMA3F-AS1	2
SEMA4C	2
SENP1	2
SENP8	2
SERAC1	2
SERPINA1	2
SESN1	2
SEZ6	2
SH2B3	2
SH3D21	2
SH3TC1	2
SHANK2-AS3	2
SHB	2
SHC3	2
SHMT2	2
SIGLEC7	2

SIGLEC8	2
SIPA1L1	2
SIRPB2	2
SIRT5	2
SKIDA1	2
SKOR1	2
SLAMF7	2
SLAMF9	2
SLC14A2	2
SLC15A2	2
SLC15A3	2
SLC15A4	2
SLC17A7	2
SLC18B1	2
SLC1A1	2
SLC20A1	2
SLC22A4	2
SLC25A20	2
SLC25A35	2
SLC26A1	2
SLC26A7	2
SLC26A8	2
SLC28A1	2
SLC28A2	2
SLC2A1	2
SLC2A3	2
SLC35G5	2
SLC37A2	2
SLC38A9	2
SLC3A2	2
SLC40A1	2
SLC45A1	2
SLC5A2	2
SLC6A16	2
SLC7A7	2
SLC8A1	2
SLC9A8	2
SLC9A9	2
SLC9B1	2
SLCO4A1	2
SLFN12L	2
SLX1B	2
SMAD5-AS1	2
SMARCAD1	2
SMIM17	2
SNAI3	2
SNAP25-AS1	2
SNHG12	2
SNHG22	2

SNRK-AS1	2
SNURF	2
SNX1	2
SNX20	2
SNX22	2
SNX25	2
SNX30	2
SOCS1	2
SOD2	2
SOS1	2
SOX30	2
SOX4	2
SP2-AS1	2
SP6	2
SPAG5	2
SPATA9	2
SPC25	2
SPDYA	2
SPDYE16	2
SPDYE3	2
SPDYE5	2
SPIN2A	2
SPINK1	2
SPOCK2	2
SPON1	2
SRBD1	2
SRCIN1	2
SRF	2
SRGN	2
SRPX	2
SRPX2	2
SSBP1	2
ST3GAL3	2
ST3GAL6-AS1	2
ST7-AS1	2
STAC2	2
STAMBPL1	2
STAP1	2
STARD13	2
STARD4	2
STARD8	2
STARD9	2
STAT2	2
STIL	2
STK38L	2
STKLD1	2
STPG2	2
STX11	2
SUCLA2-AS1	2

SUCLG2-AS1	2
SUFU	2
SWT1	2
SYNGAP1	2
SYNGR3	2
SYNJ2BP-COX16	2
SYT15	2
TAF1A	2
TAF1C	2
TAS2R10	2
TAS2R30	2
TBC1D13	2
TBC1D32	2
TBC1D3B	2
TBC1D3L	2
TBC1D4	2
TBX19	2
TCEAL6	2
TCEANC	2
TCEANC2	2
TCF23	2
TCF7L1	2
TCTE3	2
TCTEX1D1	2
TDRD7	2
TEC	2
TEFM	2
TEKT3	2
TEP1	2
TET2	2
TET3	2
TEX12	2
TEX38	2
TFAP2A-AS1	2
TFB1M	2
TFRC	2
TG	2
TGFB3	2
TGIF1	2
TGM1	2
THCAT158	2
THEMIS2	2
TIAF1	2
TICAM2	2
TICRR	2
TIFAB	2
TIGD6	2
TIMELESS	2
TIRAP	2

TK1	2
TLR10	2
TLR6	2
TM4SF19-TCTEX1D2	2
TMC3-AS1	2
TMEM133	2
TMEM150B	2
TMEM156	2
TMEM169	2
TMEM170B	2
TMEM178A	2
TMEM189-UBE2V1	2
TMEM200B	2
TMEM204	2
TMEM216	2
TMEM220-AS1	2
TMEM232	2
TMEM240	2
TMEM253	2
TMEM254-AS1	2
TMEM262	2
TMEM71	2
TMEM72-AS1	2
TMEM79	2
TMEM86A	2
TMEM86B	2
TMPPE	2
TMTC2	2
TNFAIP8	2
TNFAIP8L1	2
TNFRSF10A	2
TNFRSF10B	2
TNFRSF17	2
TNFRSF25	2
TNFRSF9	2
TNFSF10	2
TNFSF14	2
TNFSF15	2
TNFSF4	2
TNK1	2
TNKS2-AS1	2
TNNI2	2
TOLLIP-AS1	2
TOMM20L	2
TOR4A	2
TPH1	2
TPH2	2
TPI1	2
TRAF3IP2-AS1	2

TRAFD1	2
TRAIP	2
TRAV13-2	2
TRAV4	2
TRBC1	2
TREM2	2
TRGC2	2
TRIM60	2
TRIM66	2
TRMT61B	2
TRPC4	2
TRPV2	2
TRPV3	2
TSACC	2
TSC22D1-AS1	2
TSHR	2
TSLP	2
TSNARE1	2
TSPYL4	2
TSSK3	2
TSSK4	2
TTC16	2
TTC21A	2
TTC31	2
TTC34	2
TTC38	2
TTC39A-AS1	2
TTC4	2
TTC6	2
TTC7A	2
TTLL9	2
TTN	2
TUBA8	2
TUBGCP6	2
TXK	2
TXNIP	2
U2AF1L5	2
UBALD1	2
UBE2L6	2
UBE2T	2
UCKL1-AS1	2
UCN	2
UNC119B	2
UNC79	2
URB2	2
USH2A	2
USP25	2
USP30	2
USP35	2

USP40	2
USP45	2
USP46-AS1	2
UTP15	2
UTS2B	2
VASH1	2
VENTX	2
VIM	2
VPREB3	2
VSIG1	2
VWF	2
WBP2NL	2
WDFY4	2
WDPCP	2
WDR37	2
WDR64	2
WDR78	2
WDR91	2
WNT3	2
WRAP53	2
WTIP	2
WWTR1-AS1	2
XCL2	2
XKR4	2
XPO7	2
XRCC2	2
XXbac-BPG252P9.10	2
XXyac-YRM2039.3	2
YPEL4	2
ZAP70	2
ZBED3-AS1	2
ZBED8	2
ZBP1	2
ZBTB14	2
ZBTB2	2
ZBTB20-AS2	2
ZBTB20-AS4	2
ZBTB21	2
ZBTB26	2
ZBTB47	2
ZBTB6	2
ZC3H10	2
ZCCHC8	2
ZFP1	2
ZFP2	2
ZFP30	2
ZFP36L2	2
ZFP82	2
ZFPM1	2

ZGLP1	2
ZKSCAN4	2
ZNF136	2
ZNF14	2
ZNF140	2
ZNF142	2
ZNF143	2
ZNF175	2
ZNF20	2
ZNF202	2
ZNF212	2
ZNF214	2
ZNF223	2
ZNF229	2
ZNF233	2
ZNF234	2
ZNF251	2
ZNF260	2
ZNF268	2
ZNF282	2
ZNF283	2
ZNF284	2
ZNF286A	2
ZNF30	2
ZNF30-AS1	2
ZNF319	2
ZNF32-AS2	2
ZNF331	2
ZNF341	2
ZNF345	2
ZNF346	2
ZNF382	2
ZNF383	2
ZNF404	2
ZNF407	2
ZNF410	2
ZNF416	2
ZNF419	2
ZNF420	2
ZNF43	2
ZNF45	2
ZNF460	2
ZNF468	2
ZNF483	2
ZNF485	2
ZNF488	2
ZNF490	2
ZNF497	2
ZNF517	2

ZNF519	2
ZNF530	2
ZNF540	2
ZNF543	2
ZNF548	2
ZNF549	2
ZNF550	2
ZNF555	2
ZNF556	2
ZNF557	2
ZNF558	2
ZNF559-ZNF177	2
ZNF565	2
ZNF569	2
ZNF571	2
ZNF572	2
ZNF577	2
ZNF583	2
ZNF586	2
ZNF589	2
ZNF594	2
ZNF597	2
ZNF605	2
ZNF613	2
ZNF615	2
ZNF627	2
ZNF629	2
ZNF630	2
ZNF646	2
ZNF649	2
ZNF665	2
ZNF670	2
ZNF670-ZNF695	2
ZNF671	2
ZNF674-AS1	2
ZNF684	2
ZNF69	2
ZNF691	2
ZNF699	2
ZNF70	2
ZNF708	2
ZNF721	2
ZNF736	2
ZNF737	2
ZNF740	2
ZNF761	2
ZNF764	2
ZNF77	2
ZNF777	2

ZNF780B	2
ZNF784	2
ZNF786	2
ZNF787	2
ZNF792	2
ZNF831	2
ZNF836	2
ZNF84	2
ZNF843	2
ZNF850	2
ZSCAN2	2
ZSCAN26	2
ZSCAN30	2
ZSCAN5A	2
ZSWIM1	2
ZYG11A	2
